# Prevalence of child undernutrition measures and their spatio-demographic inequalities in Bangladesh: an application of multilevel Bayesian modelling

**DOI:** 10.1186/s12889-022-13170-4

**Published:** 2022-05-18

**Authors:** Sumonkanti Das, Bernard Baffour, Alice Richardson

**Affiliations:** 1 School of Demography, Australian National University, Ellery Crescent, Canberra, 2601 ACT Australia; 2 Statistical Support Network, Australian National University, Science Road, Canberra, 2601 ACT Australia

**Keywords:** Rural-urban disparities, Stunting, Small area estimation, Spatial and cross-sectional correlations, Underweight, Wasting

## Abstract

Micro-level statistics on child undernutrition are highly prioritized by stakeholders for measuring and monitoring progress on the sustainable development goals. In this regard district-representative data were collected in the Bangladesh Multiple Indicator Cluster Survey 2019 for identifying localised disparities. However, district-level estimates of undernutrition indicators - stunting, wasting and underweight - remain largely unexplored. This study aims to estimate district-level prevalence of these indicators as well as to explore their disparities at sub-national (division) and district level spatio-demographic domains cross-classified by children sex, age-groups, and place of residence. Bayesian multilevel models are developed at the sex-age-residence-district level, accounting for cross-sectional, spatial and spatio-demographic variations. The detailed domain-level predictions are aggregated to higher aggregation levels, which results in numerically consistent and reasonable estimates when compared to the design-based direct estimates. Spatio-demographic distributions of undernutrition indicators indicate south-western districts have lower vulnerability to undernutrition than north-eastern districts, and indicate significant inequalities within and between administrative hierarchies, attributable to child age and place of residence. These disparities in undernutrition at both aggregated and disaggregated spatio-demographic domains can aid policymakers in the social inclusion of the most vulnerable to meet the sustainable development goals by 2030.

**Supplementary Information:**

The online version contains supplementary material available at (10.1186/s12889-022-13170-4).

## Introduction

Malnutrition remains a critical public health problem among children under the age of five years worldwide. Malnutrition is caused by multifarious interlinked factors and has both short and long term detrimental effects [1, 2]. It affects the cognitive and physical development of children, increases the risk of infections and significantly contributes adversely to mortality and morbidity [3, 4]. There are three internationally recommended indicators to measure malnutrition and monitor child growth: stunting (low height-for-age), underweight (low weight-for-age), and wasting (low weight-for-height) [5–7].

Malnutrition significantly contributes to the global burden of several diseases [8, 9]. From the most recent global figures, undernutrition accounts for at least half of all the deaths annually in children under five [10]. The vast majority of these deaths occur in low- and middle-income countries, particularly in Africa and Asia. In the Asian region, Bangladesh has one of highest prevalence of malnutrition over the last two-decades. The recent nationwide surveys indicate that near about one-third of preschool-age children are stunted, more than one-fifth are underweight and around one-tenth are wasted [11, 12]. Though the stunting level has declined considerably over the last two-decades (from 51% in 2004 to 31% in 2017) in Bangladesh [12], the average annual rate of reduction is still below the global recommended rate of 3.9% for stunting [13]. Consequently, the country is still behind the progress to achieving the Sustainable Development Goal (SDG) target 2.2 of reducing the stunting level below 25% by 2022 [12] as well as the wasting level below 5% by 2025 [11].

The achievement in the reduction of child undernutrition significantly vary at the sub-national level [14] as well as at various socio-economic and demographic levels [15–17]. Though undernutrition levels do not significantly vary by sex at the national level, female children are more prone to undernutrition [18, 19]. A considerable amount of spatial variation in the prevalence of undernutrition indicators at the lower administrative units such as districts and sub-districts has also been observed in several studies [20–22]. However, a proper understanding of the spatio-demographic variation (say, the interaction of administrative units, place of residence, children sex and age) in the prevalence of undernutrition has remained unknown. An expansive range of literature has been devoted to understanding malnutrition and the socio-demographic and spatial determinants through multilevel modelling (for example, [23–27]).

To eradicate all forms of malnutrition in line with the SDG targets, the inequalities in the undernutrition vulnerability at the spatial, demographic and spatio-demographic level should be reduced as per the SDG 10 goal of reducing inequalities within country. Proper allocation of resources and policy-making by focusing on the disaggregated administrative units can only help to reduce this inequality. In this regards, reliable statistical information at detailed administrative units as well as their cross-classified demographic domains are required. Since nationally representative surveys fail to provide such precise disaggregated level statistics, small area estimation [28] has been widely used in poverty estimation [29–31], labour force [32], food insecurity [33, 34], and child undernutrition [20, 21, 35]. In this work, we use a multilevel framework to incorporate contextual factors and increase the precision of estimates under small area estimation for enhanced inference of malnutrition indicators.

In this study, we use a multilevel modelling extension of the small area estimation method to estimate stunting, wasting and underweight for spatio-demographic domains of division and districts using the recent Multiple Indicator Cluster Survey (MICS) 2019 and Bangladesh Population and Housing census 2011 data. The considered multilevel models, expressed within a hierarchical Bayesian framework, are developed at the detailed level cross-classified domains of sixty-four districts by children’s sex (female and male), five age-groups (0-11, 12-23, 24-35, 36-47, and 48-59 months), place of residence (rural and urban). These multilevel models are known to reduce uncertainty by including variability in the outcome variables at various aggregation levels and hence provide consistent estimates for the domains with very small samples in the survey data [36]. This is accomplished through borrowing strength by accounting for cross-sectional correlation from similar domains, and spatial correlation from the neighbouring domains [37, 38]. Spatio-demographic district level estimates are obtained directly from the developed multilevel models and then these disaggregated estimates are aggregated to obtain district, division and national level estimates, which provides numerically consistent estimates for the disaggregated level domains. Finally, the spatial distribution of children undernutrition indicators as well as their consistency are plotted in bivariate interactive maps.

## Materials and methods

### Data source and input estimates

The microdata of children nutrition status have been extracted from the Bangladesh MICS 2019. The sampling plan of MICS 2019 was designed to produce consistent estimates of population, health and nutrition indicators at division (first administrative geography) and district (second administrative geography) levels. In this regard, rural and urban parts of each district are considered as sampling strata. Then a two-stage stratified sampling procedure is followed: clusters, which are census enumeration areas in the 2011 Census of Bangladesh, are selected from each stratum systematically with probability proportional to size. Subsequently, 20 households were selected from each of the selected cluster. Finally, a total of 64,400 households (in 3,220 clusters) were enumerated in the MICS 2019 sample.

Weights and heights for a total of 24,686 children under 5 years of age were measured in MICS 2019 to calculate their anthropometric indices for height-for-age, weight-for-age and weight-for-height expressed in standard deviation units (z-scores) from the median of the reference population. Finally, anthropometric information of 22,106, 22,484, and 22,063 children were eligible to calculate height-for-age z-scores, weight-for-age z-scores and weight-for-height z-score respectively. A child is considered as stunted, underweight and wasted when his/her z-scores are below -2.0 standard deviation units respectively [6]. 

**Fig. 1:**
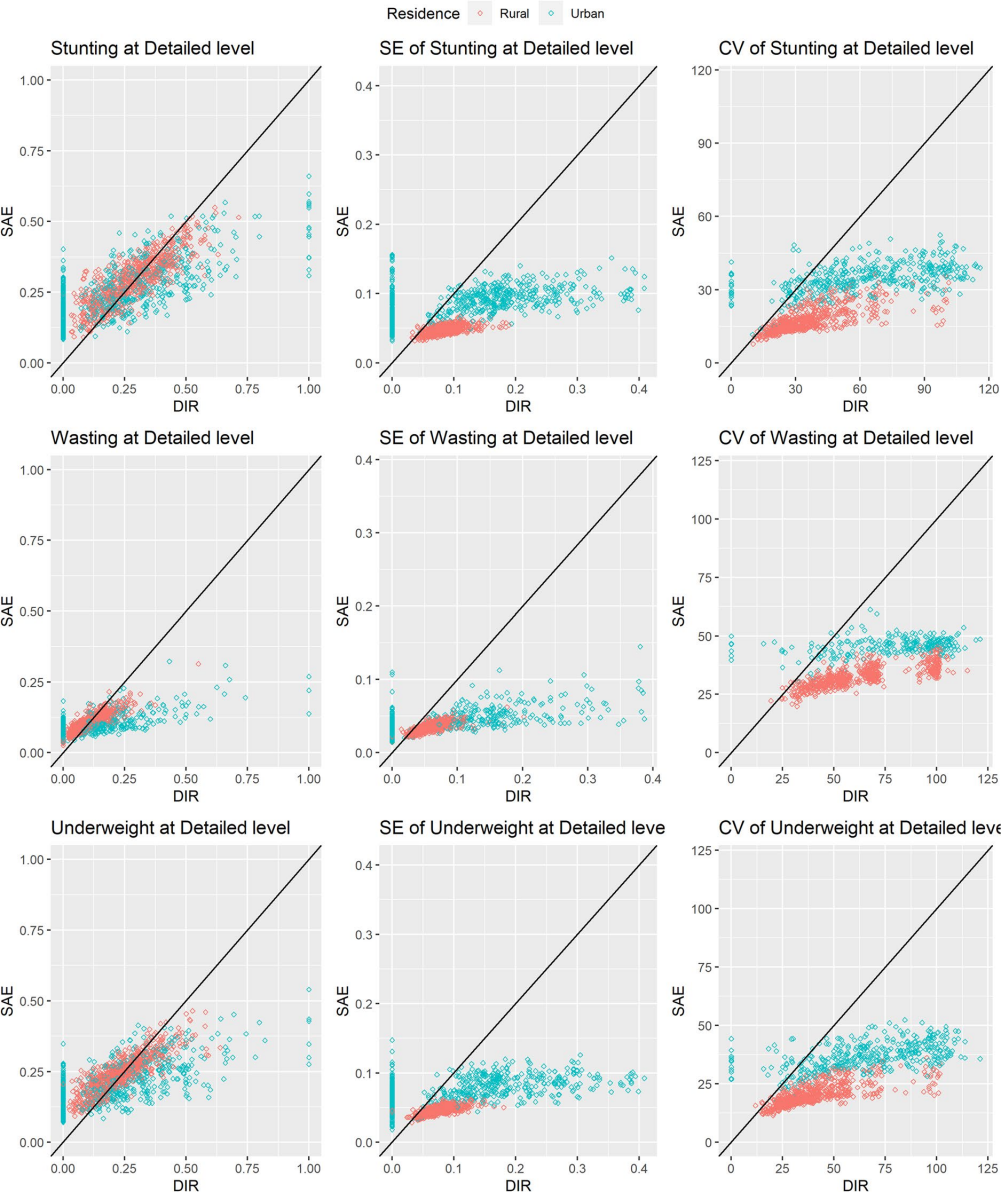
Standard errors (SE) and generalized variance function (GVF) model-based SEs are plotted against the corresponding estimates under original and square-root scales for stunting, wasting and underweight. Empty diamonds and filled circles refer to direct and GVF model-based SEs. Orange and sky-blue colours refer to rural and urban domains, respectively

Standard errors of the estimated prevalence of stunting, wasting and underweight for each of the target domains are calculated by accounting for the sampling design and sampling weights through the Taylor series linearisation method [11]. The standard errors (SEs) for the urban domains are comparatively very higher and noisy than those of rural domains due to very small sample in urban domains (see Supplementary Fig. S.1). Since these variance estimates are treated as fixed and known in the hierarchical Bayesian model as the Fay-Herriot area-level model [39], they are smoothed using Generalized Variance Functions (GVF) method ([40], Chapter 7) following the approach described in [38]. Figure 1 shows that the direct SEs are positively correlated with the estimates for all indicators and also the direct input estimates are very noisy for urban domains. As such, some transformations were examined to reduce the high dependencies among the estimates and their SEs. We observe that the square-root (*sqrt*) transformation reduces this correlations and also stabilizes the variance estimates. For the *sqrt* transformation case, the GVF model is developed under *sqrt* scale instead of original scale following [38]. The GVF models are given in Supplementary Tables S.1-S.3. Therefore, the *sqrt* transformed estimates and their GVF model-based smoothed SEs are used for subsequent model development.

### Multilevel Bayesian model

To define the multilevel Bayesian model, let $\hat {Y} = (\hat {Y}_{1}, \hat {Y}_{1}, \ldots \hat {Y}_{D})$ denote vector of dimension *D*=64×2×5×2=1280, where $\hat {Y}_{d}, d=1,2,\ldots D$ denotes the direct estimate of any childhood undernutrition indicator for domain *d*. Following [38], a multilevel Bayesian model for $\hat {Y}$ can be written as

1 $$\hat{Y} = X\beta + \sum_{i} Z^{(i)} v^{(i)} + e,$$

where *X* is a design matrix of *D*×*p*, *β* is a *p*-vector of fixed effects, *Z*^(*i*)^ are *D*×*q*^(*i*)^ design matrices for *q*^(*i*)^-dimensional random effect vectors *v*^(*i*)^, and *e*=(*e*_1_,…,*e*_*D*_)^′^ is the vector of sampling errors. The first part of the right hand side refers to fixed effects and the second part refers to inclusion of several random effects terms defined at different levels (e.g., district and spatio-demographic domains) simultaneously. The sampling errors are assumed normally distributed as $e \sim {\mathcal {N}}(0, \Sigma)$, where the covariance matrix *Σ* can be a full or diagonal matrix of dimension *D*×*D*. In this study, *Σ* is considered as a diagonal matrix with the above mentioned smoothed error variances. Assuming normality of the sampling errors *e*, the likelihood function conditional to the fixed and random effects parameters can be defined as

2 $$p \left(\hat{Y} \mid \eta,\Sigma \right) = N \left(\hat{Y} \mid \eta, \Sigma \right),$$

where $\eta = X\beta + \sum _{\alpha } Z^{(i)} v^{(i)}$ is the linear predictor.

Each of the random effect vectors are formed based on two factor variables. For example, five levels of the *Age* variable are assumed to vary over 64 levels of the *District* variable. As such, the former factor variable (*Age*) with *d*=5 levels and the later variable (*District*) with *l*=64 levels constitute a random effect vector of length *q*=*d*×*l*=5×64 with variance covariance matrix *A*⊗*V* where *V* and *A* are respectively *d*×*d* and *l*×*l* covariance matrices. The covariance matrix *A* is assumed known and its precision matrix *Q*_*A*_=*A*^−1^ (instead of *A*) is used because of computational efficiency [41]. The covariance matrix *V* for the *d* varying effects can be parameterized as a full covariance matrix (or diagonal matrix) with unequal (or equal) diagonal elements.

A number of models structured as (1) were fitted using Markov Chain Monte Carlo simulation method for each of the undernutrition indicators in R [42] using package mcmcsae [43]. The details of model formulation are given in the Supplementary sub-section S.3 based on [37, 38].

## Model development

For each undernutrition indicator, initially the main effects of children demographic variables (*Age*, *Sex*, *Residence*) along with the regional variable *Division* are used as fixed effect components. As a random effect (RE) component (denoted by *WN*_*DIST*), domain specific random effects are assumed to follow the normal distribution with a common variance. This initial model with *WN*_*DIST* component is a perfect example of the Fay-Herriot model [39]. Since this model failed to account for variation by place of residence, interaction effects of *Residence* with *Age*, *Sex* and *Division* are examined as fixed effects, initially. However, it was found that inclusion of these interaction effects as random effects improved the model further in terms of the model selection criteria, i.e., the Widely Applicable Information Criterion or Watanabe-Akaike Information Criterion (WAIC) [44, 45] and the Deviance Information Criterion (DIC) [46]. In this regard, a number of random effect components defined at various aggregation levels shown in Table 1 were included by specifying separate variance parameters for rural and urban domains. Three residence specific random effects components are defined at *District* (denoted by *RES*_*DIST*), *District*×*Age* (denoted by *RES*_*DIST*_*AGE*) and *District*×*Sex* (denoted by *RES*_*DIST*_*SEX*) level. The random effects under *RES*_*DIST* component can be interpreted as random intercepts for the domains cross-classified by *District* and *Residence* following normal distributions with different variance components for rural and urban domains.

**Table 1:** Summary of the random effect components for the multilevel models for stunting, wasting and underweight. The second and third columns refer to the varying effects with covariance matrix *V*, whereas the fourth column refers to the factor variable associated with *A*. The last two columns contain the total number of parameters and random effects for each random effects component

Model Component	Factor V	Variance Structure	Factor A	Parameters	Number of Effects
RES_DIST	*Residence*	diagonal	*District*	2	128
RES_DIST_AGE	*Residence*	diagonal	*District*×*Age*	2	640
RES_DIST_SEX	*Residence*	diagonal	*District*×*Sex*	2	640
AGE_DIST	*Age*	diagonal	*District*	5	320
SP_DIST	1	scalar	Spatial(*District*)	1	64
WN_DIV	1	scalar	*Division*×*Residence*×*Age*×*Sex*	1	160
WN_DIST	1	scalar	*District*×*Residence*×*Age*×*Sex*	1	1280

In a similar way, age-specific differentials at district level are accounted for by an age-specific random effect component (denoted by *AGE*_*DIST*) with diagonal variance structure (See Table 1). The full covariance structure of *V* for the *AGE*_*DIST* component has an additional 10 correlation parameters. However, inclusion of a spatial component (denoted by *SP*_*DIST*) in the model to include spatial correlations among district through a conditional autoregressive (CAR) model [47] reduced the importance of these additional correlation parameters of *AGE*_*DIST* component. So, the diagonal variance structure is used for *AGE*_*DIST* to borrow strength over space by children age-groups. The component *SP_DIST* indicates spatial random effects for each of 64 districts conditional on the others, and are assumed to vary with equal variance. This spatial component mainly accounts for the spatial variation of undernutrition vulnerability over sub-regions. A similar approach was taken in the specification of the spatial random effect terms in [47] and [41]. Finally, like the *WN*_*DIST* component, a division level random effects component (denoted by *WN*_*DIV*) is added to specify random intercept for division level cross-classified domains of *Age*, *Sex* and *Residence*. The notation of the variance parameters related to the random effect components are shown in Table 2.

**Table 2:** Posterior mean of random effect parameters and their t-values in the multilevel models of stunting, wasting and underweight

	Indicator	Stunting		Wasting		Underweight	
RE Component	Parameter	Mean	t-value	Mean	t-value	Mean	t-value
RES_DIST	$\sigma _{1R}^{2}$	-	-	0.018	1.517	0.029	2.591^**^
	$\sigma _{1U}^{2}$	-	-	0.062	3.136^*^	0.047	1.982^**^
RES_DIST_AGE	$\sigma _{2R}^{2}$	0.011	1.394	0.018	1.517	0.011	1.396
	$\sigma _{2U}^{2}$	0.118	9.693^*^	0.062	3.136^*^	0.084	5.371^*^
RES_DIST_SEX	$\sigma _{3R}^{2}$	0.008	1.381	0.007	1.314	0.010	1.431
	$\sigma _{3U}^{2}$	0.055	3.585^*^	0.070	4.222^*^	0.082	5.173^*^
AGE_DIST	$\sigma _{41}^{2}$	0.067	4.809^*^	0.057	3.557^*^	0.062	4.437^*^
	$\sigma _{42}^{2}$	0.042	2.628^**^	0.059	3.961^*^	0.046	3.012^*^
	$\sigma _{43}^{2}$	0.018	1.501	0.027	1.717	0.017	1.468
	$\sigma _{44}^{2}$	0.013	1.367	0.023	1.587	0.018	1.463
	$\sigma _{45}^{2}$	0.036	2.254^**^	0.051	3.368^*^	0.019	1.548
SP_DIST	$\sigma _{5}^{2}$	0.073	5.429^*^	-	-	0.036	1.958^**^
WN_Division	$\sigma _{6}^{2}$	0.022	3.151^*^	0.021	2.618^**^	0.018	2.363^**^
WN_District	$\sigma _{7}^{2}$	0.026	2.327^**^	0.044	4.235^**^	0.036	3.208^*^

*p-value < 0.01, **p-value < 0.05

After extensive examination, the main effects of the cross-classification and division variables are considered as fixed effects in the models of all three indicators. The posterior mean and the corresponding t-value for the fixed effects parameters are shown in Table 3. Table 3 shows that the cross-classified domains of children aged 12-23 months have higher stunting and underweight levels but lower wasting level compared to the cross-classified domains of infant children (0-11 months). However, stunting and underweight levels are found to be highest among 24-35 months old children. Domains belong to the Sylhet division also experience higher levels of stunting and underweight. Though at the national level there was no significant difference by residence and sex, urban domains seem to experience lower stunting and underweight levels, while male children were observed to have higher wasting levels compared to females.

**Table 3:** Posterior mean of fixed effect parameters and their t-values in the multilevel models of stunting, wasting and underweight

	Indicator	Stunting		Wasting		Underweight	
Variables	values	Mean	t-value	Mean	t-value	Mean	t-value
Intercept	-	0.396	14.989^*^	0.294	12.800^*^	0.412	15.800^*^
Age	0-11	-	-	-	-	-	-
	12-23	0.127	7.606^*^	-0.111	-8.320^*^	0.064	3.880^*^
	24-35	0.169	10.428^*^	0.018	1.050	0.107	6.979^*^
	36-47	0.147	9.331^*^	0.000	0.026	0.100	6.479^*^
	48-59	0.088	5.262^*^	-0.008	-0.530	0.102	6.637^*^
Division	Barishal	-	-	-	-	-	-
	Chattogram	0.030	1.091	0.015	0.899	-0.009	-0.318
	Dhaka	0.015	0.500	-0.007	-0.307	-0.039	-1.386
	Khulna	-0.040	-1.392	-0.038	-1.580	-0.045	-1.577
	Mymensingh	0.089	2.480^**^	-0.017	-0.709	0.043	1.242
	Rajshahi	0.017	0.593	0.014	0.451	0.001	0.044
	Rangpur	0.019	0.608	-0.024	-0.913	-0.009	-0.298
	Sylhet	0.110	2.992^*^	0.006	0.242	0.100	2.800^*^
Residence	Rural	-	-	-	-	-	-
	Urban	-0.091	-7.276^*^	0.020	0.665	-0.107	-7.182^*^
Sex	Female	-	-	-	-	-	-
	Male	0.005	0.564	0.027	3.014^*^	-0.004	-0.463

*p-value < 0.01, **p-value < 0.05

All the random effects specified in Table 1 had a significant contribution only in the selected underweight model (see Table 2). However, the variance parameters of *RES*_*DIST* component in stunting model and the variance parameter of *SP*_*DIST* in wasting model were not significant and so they are removed from the final models. According to the t-statistics, the *SP*_*DIST* component seems to have higher contribution in the stunting model than the underweight model. The variance parameters of *RES*_*DIST*_*AGE* and *RES*_*DIST*_*SEX* components indicate that random effects of urban domains have higher variance than those of rural domains for all the three indicators. The *AGE*_*DIST* component indicates random effects for the domains relating to infant (0-11) and toddler (12-23) children have comparatively higher variances. Variance estimates of *WN*_*DIV* and *WN*_*DIST* components reveal that variations at the disaggregated level domains are still significant for all the three indicators after accounting for the variations at the district level cross-classified domains, which are two- and three-order interactions of districts with children age, sex and residence.

Information criteria WAIC and DIC were utilized to find the best model among the models developed with same input estimates for each of the three undernutrition indicators. However, final models are assessed based on some model diagnostics such as relative bias (RB), absolute relative bias (ARB), relative reduction of the standard errors (RRSE), and the ratio of coefficients of variation (CV) following [48]. The detail of these diagnostics are given in the Supplementary sub-section S.4 along with the relevant results. The summary of the model assessment are: for all the indicators the values of RB, ARB and RRSE increase with disaggregation level, while the CV ratios decrease. These are expected since the direct estimates are more reliable and consistent at the higher aggregation level than the disaggregation level. On the other hand, the SAE estimates are as consistent as the DIR estimates at the higher aggregation level, while at the disaggregated level SAE estimates are more precise than the DIR estimates [38]. The SAE estimates appear to provide more gains for the cross-classified domains of district. Interestingly the highest gains, in terms of RRSE and ratio of CVs, are observed at the most detailed level (*District*×*Residence*×*Age*×*Sex*) at which the multilevel models are developed.

## Results

Multilevel models are developed on square-root scale transformation, and so model predictions are back-transformed to original scale with a small bias correction following [38] to obtain the prevalence of stunting, wasting and underweight along with their SEs. The prevalence of stunting, wasting and underweight at the national level are estimated as about 29%, 10% and 23% based on the developed multilevel models (Table 4). These estimates are very close to the direct estimates (28.0%, 9.8% and 22.6% respectively) documented in the Bangladesh MICS 2019 report [11]. The prevalence of these undernutrition indicators does not vary much by sex, however it varies considerably by children age-groups. The levels of wasting (10.6% in rural and 8.5% in urban) and underweight (24.7% in rural and 19.7% in urban) vary by place of residence compared to their magnitude at the national level. The levels of both stunting and underweight follow an increasing trend with the children age-groups up to 24-35 months (35.9% for stunting and 26.4% for underweight) and then reduce, but the reduction rate is higher for stunting than that of underweight. Though the children belonging to 24-35 months age-group are highly vulnerable to both stunting and underweight, they seem less vulnerable to wasting. Children belonging to 0-11, 12-23 and 48-59 months age-groups are more vulnerable to wasting. At division level, the prevalence of stunting and underweight are higher in Sylhet (38.5% and 33.1% respectively) and Mymensingh (34.4% and 26.3% respectively) divisions and lower in Khulna division (22.3% and 20.2% respectively). Though the prevalence of underweight (20.2%) and wasting (9.0%) are lower in Dhaka division, stunting is considerably higher (29.1%) than in Khulna division.

**Table 4:** Estimated prevalence (standard errors) of stunting, wasting and underweight at children sex, age-group, residence, and division level as well as for cross-classified domains of division

Variable	Category	Barishal	Chattogram	Dhaka	Mymensingh	Khulna	Rajshahi	Rangpur	Sylhet	Total
Stunting										
Sex	Female	31.9 (1.3)	27.7 (0.9)	29.0 (1.0)	34.1 (1.6)	22.1 (1.0)	28.2 (1.2)	27.9 (1.2)	38.7 (1.5)	29.1 (0.5)
	Male	32.1 (1.3)	29.1 (0.9)	29.3 (0.9)	34.6 (1.6)	22.4 (0.9)	27.5 (1.1)	27.6 (1.1)	38.4 (1.5)	29.4 (0.4)
Age	0-11	16.6 (1.8)	17.4 (1.3)	20.4 (1.4)	25.9 (2.3)	14.6 (1.3)	18.6 (1.5)	21.1 (2.1)	24.6 (2.1)	19.6 (0.6)
	12-23	33.5 (2.0)	29.5 (1.5)	33.0 (1.7)	38.6 (2.7)	23.1 (1.5)	29.3 (1.8)	29.7 (1.8)	40.4 (2.5)	31.4 (0.8)
	24-35	42.4 (2.1)	34.1 (1.5)	37.0 (1.6)	39.5 (2.6)	27.9 (1.6)	35.0 (1.9)	32.9 (1.8)	45.2 (2.4)	35.9 (0.8)
	36-47	38.4 (2.0)	32.4 (1.4)	31.5 (1.4)	37.6 (2.5)	25.1 (1.4)	31.0 (1.7)	31.1 (1.7)	44.7 (2.4)	32.8 (0.7)
	48-59	27.1 (2.0)	28.2 (1.4)	23.9 (1.4)	31.8 (2.4)	20.3 (1.4)	25.7 (1.7)	24.3 (1.6)	38.5 (2.4)	26.6 (0.7)
Residence	Rural	32.6 (1.1)	29.3 (0.8)	28.2 (0.8)	35.3 (1.4)	23.0 (0.9)	28.6 (1.0)	28.4 (1.0)	39.2 (1.3)	29.7 (0.4)
	Urban	28.5 (2.2)	25.2 (1.4)	31.1 (1.4)	28.8 (2.9)	18.6 (1.3)	24.4 (1.7)	23.4 (1.5)	34.5 (2.9)	27.4 (0.7)
Total		32.0 (1.1)	28.4 (0.7)	29.1 (0.8)	34.4 (1.3)	22.3 (0.8)	27.8 (0.9)	27.7 (0.9)	38.5 (1.2)	29.3 (0.3)
Underweight										
Sex	Female	25.8 (1.2)	23.9 (0.8)	20.0 (0.8)	25.9 (1.5)	20.5 (0.9)	25.0 (1.1)	24.3 (1.1)	34.3 (1.4)	23.9 (0.4)
	Male	25.9 (1.2)	23.6 (0.8)	20.4 (0.8)	26.6 (1.5)	20.0 (0.9)	23.9 (1.1)	23.0 (1.0)	32.0 (1.4)	23.5 (0.4)
Age	0-11	18.4 (1.7)	15.8 (1.2)	17.6 (1.2)	18.4 (2.0)	14.2 (1.3)	18.7 (1.5)	17.1 (1.4)	25.8 (1.9)	17.7 (0.6)
	12-23	24.0 (1.8)	23.4 (1.3)	20.0 (1.3)	24.7 (2.3)	18.3 (1.3)	23.0 (1.7)	23.6 (1.6)	32.5 (2.2)	22.8 (0.7)
	24-35	30.4 (1.9)	26.2 (1.3)	22.5 (1.2)	28.4 (2.3)	23.0 (1.4)	26.9 (1.7)	26.4 (1.6)	38.2 (2.2)	26.4 (0.7)
	36-47	28.3 (1.8)	25.5 (1.3)	20.5 (1.2)	30.8 (2.3)	23.0 (1.4)	26.5 (1.6)	25.4 (1.5)	35.3 (2.1)	25.5 (0.6)
	48-59	27.3 (1.9)	27.7 (1.4)	20.5 (1.2)	29.6 (2.2)	22.4 (1.4)	26.6 (1.6)	25.8 (1.6)	34.4 (2.1)	25.8 (0.7)
Residence	Rural	26.2 (1.1)	24.3 (0.7)	21.7 (0.7)	27.2 (1.3)	20.9 (0.8)	25.4 (1.0)	24.3 (0.9)	33.7 (1.2)	24.7 (0.3)
	Urban	23.8 (2.2)	21.7 (1.3)	17.3 (1.1)	20.3 (2.4)	16.9 (1.3)	19.8 (1.6)	19.0 (1.5)	29.8 (2.9)	19.7 (0.6)
Total		25.8 (1.0)	23.8 (0.6)	20.2 (0.6)	26.3 (1.2)	20.2 (0.7)	24.4 (0.9)	23.6 (0.8)	33.1 (1.2)	23.7 (0.3)
Wasting										
Sex	Female	10.5 (0.8)	10.1 (0.6)	8.4 (0.5)	9.5 (0.9)	9.0 (0.6)	8.8 (0.7)	10.5 (0.7)	10.9 (0.9)	9.5 (0.3)
	Male	11.7 (0.8)	11.0 (0.6)	9.5 (0.6)	11.1 (0.9)	10.7 (0.7)	10.7 (0.7)	11.4 (0.7)	12.1 (0.9)	10.8 (0.3)
Age	0-11	11.6 (1.4)	11.0 (0.9)	10.4 (1.0)	10.3 (1.4)	9.3 (1.0)	8.7 (1.0)	8.4 (1.0)	12.0 (1.4)	10.2 (0.5)
	12-23	12.7 (1.3)	12.8 (1.0)	9.4 (0.9)	8.3 (1.3)	10.1 (1.0)	10.5 (1.2)	12.6 (1.3)	13.8 (1.6)	11.1 (0.5)
	24-35	10.5 (1.2)	8.7 (0.8)	8.3 (0.8)	10.9 (1.5)	9.5 (1.0)	9.3 (1.0)	11.7 (1.1)	11.1 (1.4)	9.5 (0.4)
	36-47	9.2 (1.1)	8.6 (0.8)	7.2 (0.7)	10.9 (1.4)	9.6 (0.9)	9.2 (1.0)	10.5 (1.0)	9.7 (1.2)	9.0 (0.4)
	48-59	11.7 (1.4)	11.8 (1.0)	9.7 (1.0)	11.1 (1.5)	10.8 (1.1)	11.3 (1.2)	12.1 (1.2)	11.5 (1.5)	11.1 (0.5)
Residence	Rural	11.2 (0.7)	11.0 (0.5)	9.4 (0.5)	10.6 (0.8)	10.2 (0.6)	10.2 (0.6)	11.5 (0.7)	11.9 (0.8)	10.6 (0.2)
	Urban	10.5 (1.2)	8.8 (0.8)	8.1 (0.8)	8.2 (1.0)	8.4 (0.8)	8.5 (0.9)	7.9 (0.8)	9.8 (1.4)	8.5 (0.4)
Total		11.1 (0.7)	10.6 (0.5)	9.0 (0.4)	10.3 (0.8)	9.8 (0.5)	9.9 (0.6)	11.0 (0.6)	11.6 (0.8)	10.2 (0.2)

The variations in the prevalence of stunting, wasting and underweight by children age, sex, and place of residence are expected to increase for their cross-classified domains with division and district. These spatio-demographic variations are explored in the following two sub-sections, along with the spatial distribution of child undernurition vulnerability at the district level through mapping the prevalence levels.

### Spatio-demographic variation at division level

The prevalence levels of stunting, wasting and underweight for the cross-classified domains of division with children sex, age and place of residence are shown in Table 4. Like the national level, there is no sex difference in the division level prevalence of child undernutrition. However, rural-urban difference increases at the division level for all the indicators. The prevalence of stunting is found higher in rural parts of all divisions except that it is reversed in Dhaka division, with 31.1% of urban children are stunted compared to 28.2% of rural children. The rural-urban difference has doubled in Khulna division (23.0% vs 18.6%) compared to the difference at national level (29.7% vs 27.4% for stunting). The highest rural-urban difference is observed in Mymensingh division for both stunting (35.3% vs 28.8%) and underweight (27.2% vs 20.3%).

The increasing trends of stunting and underweight with children age-groups are also visible for all the divisions. The stunting and underweight levels have picked up to 45.2% and 38.2% respectively in Sylhet division followed by 42.4% and 30.4% in Barishal division among the 24-35 months old children (Table 4). The concerning issue is that the highest level of stunting and underweight for all age-groups are both found in Sylhet division. However, infants (0 - 11 months) of Khulna division have experienced lowest level of stunting (14.6%) and underweight (14.2%). For the most vulnerable 24-35 months children, the level of stunting and underweight increased to only 27.9% and 23.0% respectively in Khulna division. In the case of wasting, no age-specific pattern is obvious by division (Table 4). However, the wasting level in Sylhet and Barishal divisions are more than 12% for the younger two age-groups 0-11 and 12-23 months.

**Fig. 2:**
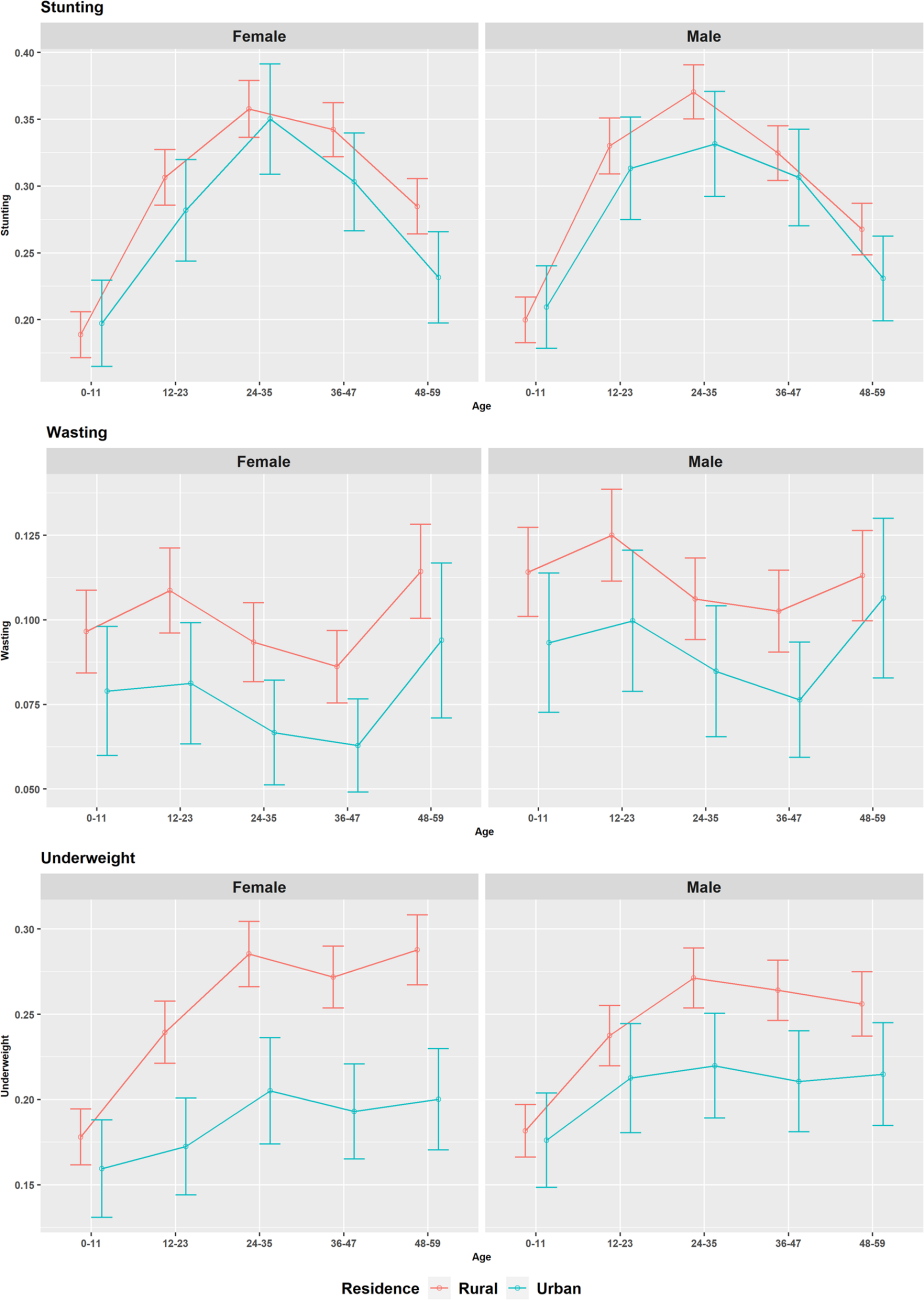
Prevalence of stunting, wasting and underweight for the cross-classified domains of children age-group, sex and place of residence

Undernutrition vulnerability does not vary considerably by sex and residence, however it varies considerably for the cross-classified domains of children age-group, sex and place of residence shown in Fig. 2. Undernutrition levels are expected to be lower in urban domains, however for the infants, stunting level is found to be slightly higher for the urban domains than the rural domains. Though rural-urban difference is not obvious for stunting, the difference is significant for both wasting and underweight. For underweight, rural-urban differences have increased with the child age-groups and this difference is higher for the female children. The prevalence of stunting and underweight has increased until 24-35 months and then they decline for stunting but remain relatively stable for underweight with different slope by sex. As for example, the underweight levels sharply increased with children age-groups for female children compared to the male children. For wasting, there are no specific trends but the prevalence remains high for the 0-11, 12-23 and 48-59 months age-groups for both male and female children. Figure 2 also indicates that the confidence bands for urban domains are wider than the rural domains even at this higher aggregation levels.

**Fig. 3:**
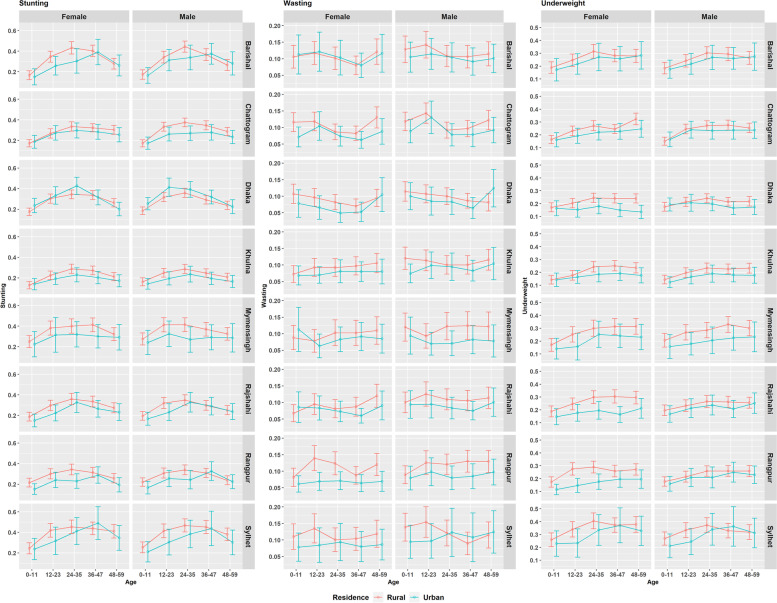
Prevalence of stunting, wasting and underweight for the cross-classified domains of children age-group, sex, place of residence and division

Figure 3 shows age-sex-residence level differences by division for all the undernutrition indicators. At a glance, Fig. 3 shows considerable rural-urban differences at different degrees by children age-groups and division. For example, the difference is considerably higher for the female children belonging to 12-23 and 24-35 months age-groups of Rangpur division for all indicators. For underweight, the rural-urban differences seem higher among the female older age-groups (more specifically 24+ months age-groups) in Dhaka and Rajshahi divisions. More interestingly, this rural-urban difference in the prevalence of underweight increases with the age-groups (which pattern is also observed in Fig. 2). This difference occurs due to stable estimates for rural domains compared to the declining trends in urban domains. In Sylhet and Barishal divisions, the prevalence of stunting increases steadily up to age-group 36-47 months in the urban domains, while the trends of rural domains follow the overall trend of increasing up to 24-35 months and then declining.

### Spatio-demographic variation at district level

**Fig. 4:**
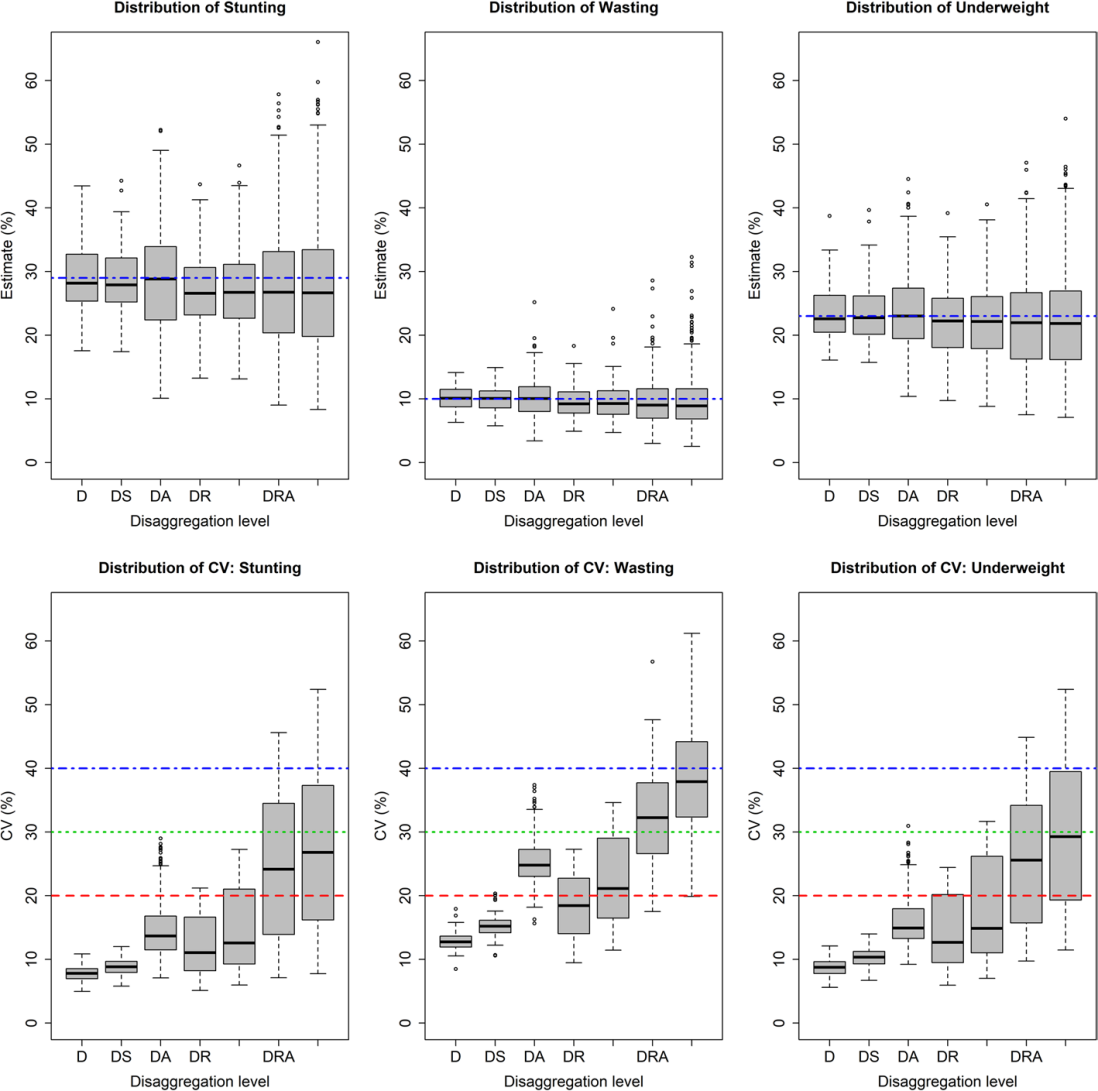
Distribution of estimated stunting, wasting and underweight (in %) with their coefficient of variation (CV in %) for the disaggregation level of district (D), district-sex (DS), district-age (DA), district-residence (DR), district-residence-sex (DRS), district-residence-age (DRA), and district-residence-age-sex (DRAS)

Since the MICS 2019 considered district in the sampling design, it is expected that the direct estimates of district level stunting, wasting and underweight are relatively consistent. However, the model-based estimates along with their accuracy for the cross-classified domains constructed by interaction of district, residence, children sex, and children age are expected to vary with the order of interaction. The distribution of estimated stunting, wasting and underweight based on the developed multilevel models are shown in Fig. 4 (box-plots of top row) along their CVs (box-plots of bottom row) for districts (denoted by **D**) and their cross-classified domains with place of residence (**R**), children sex (**S**), and age-group (**A**). The spread of the estimates (which also represents inequality in the domains) increase with the order of interactions as expected, while spread of CVs (which represents relative accuracy of the estimates) increase exponentially. The distribution of CVs indicates that the estimates are more accurate for stunting and underweight than those for wasting. For stunting and underweight, the values of CV are below 20% for most of the domains constructed from second-order interactions of districts, while the range increase to 30% for third-order interactions and to 40% for the detailed level domains. Figure 4 shows that the values of CV are comparatively higher for wasting than those for stunting and underweight even at the district level. At the detailed level, the values of CV considerably vary over the ranges of 8-53%, 20-61% and 11-52% for stunting, wasting and underweight respectively (please see Supplementary Table S.5). These differences among CVs are expected according to the mean level of stunting (29%), wasting (10%) and underweight (23%) as the denominator of CV.

**Fig. 5:**
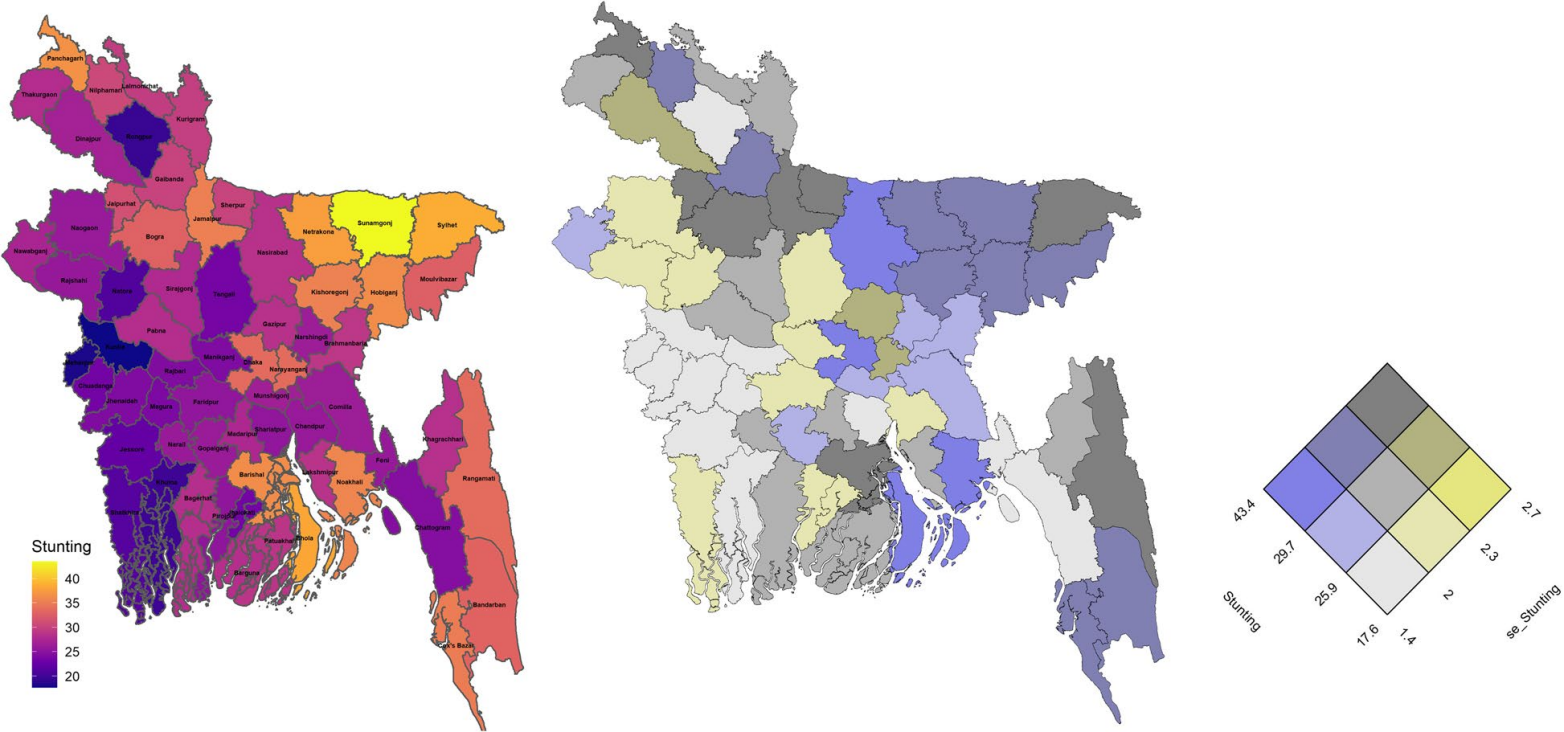
Spatial distribution of district level stunting through univariate map (left), bivariate map (middle) and bivariate key for the prevalence estimates and their standard errors (right)

**Fig. 6:**
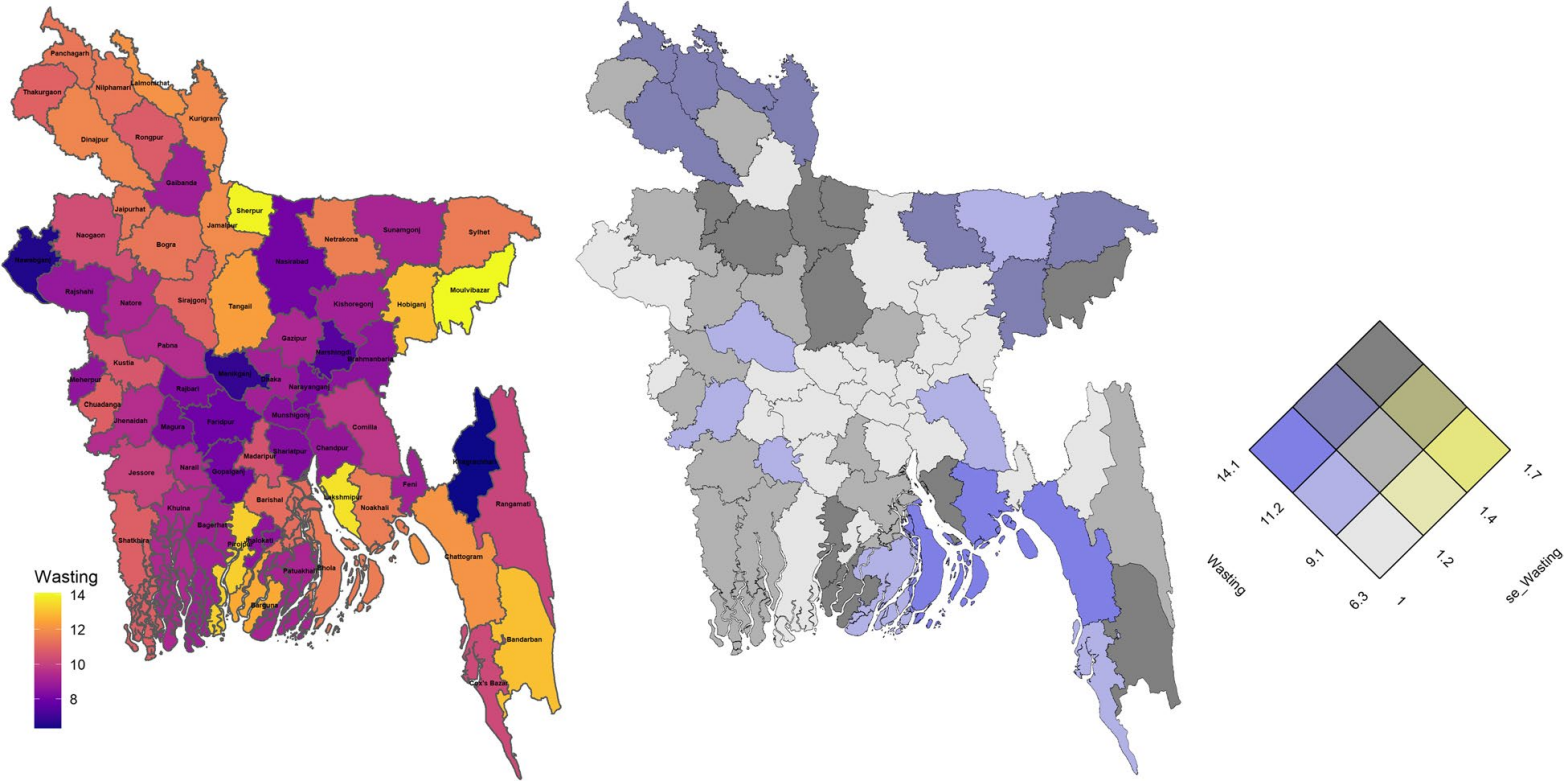
Spatial distribution of district level wasting through univariate map (left), bivariate map (middle) and bivariate key for the prevalence estimates and their standard errors (right)

**Fig. 7:**
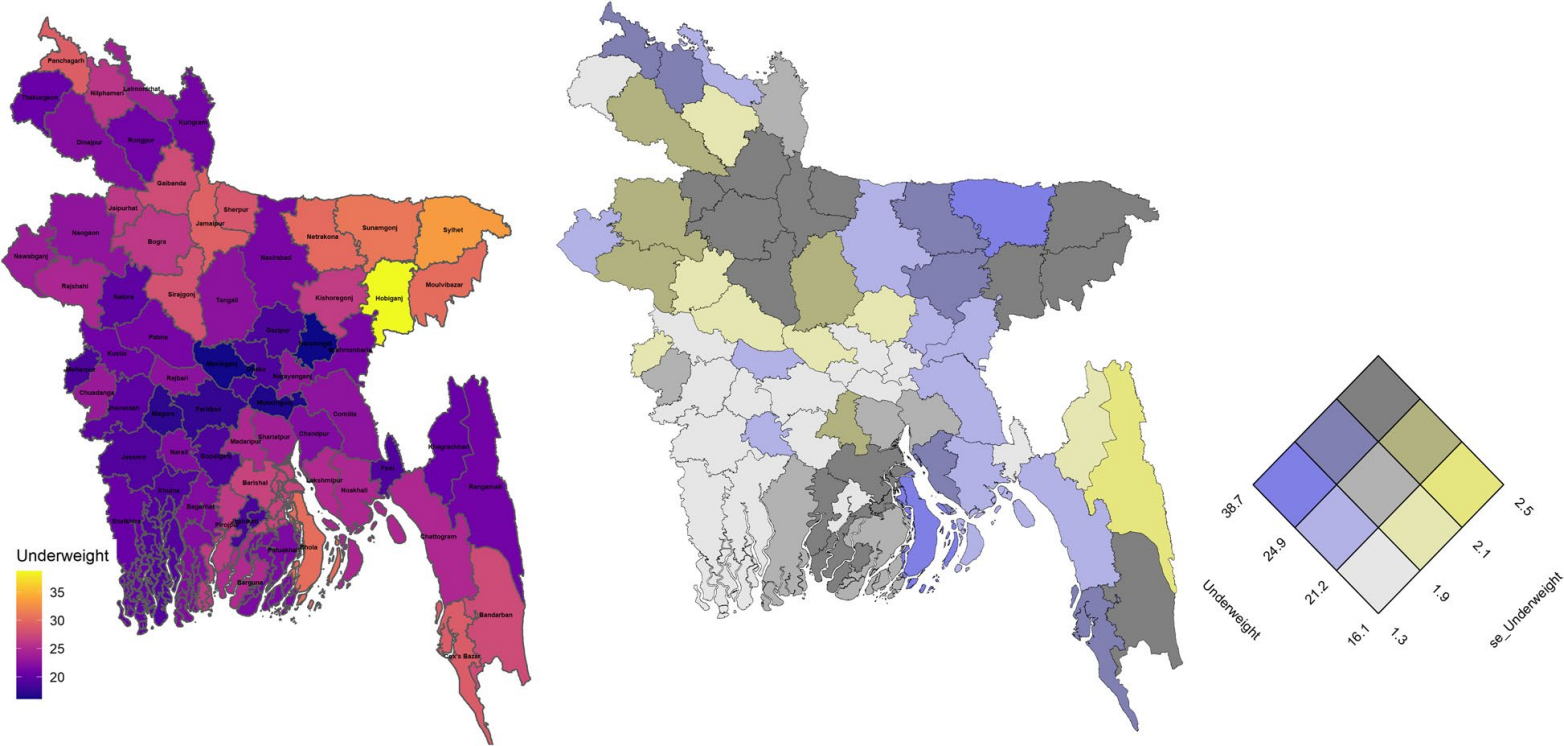
Spatial distribution of district level underweight through univariate map (left), bivariate map (middle) and bivariate key for the prevalence estimates and their standard errors (right)

District level prevalence of stunting, wasting and underweight are mapped in Figs. 5, 6 and 7 as univariate and bivariate choropleth maps. The bivariate choropleth maps are created through the “Vizumap” R package [49], where the estimates and their accuracy in terms of SEs are shown simultaneously with single colour by encoding them in a bivariate grid of terciles. For example, the grey color of the first diagonal grid of Fig. 5 indicates lower estimates of stunting with higher accuracy (grid of 1st tercile). The constructed bivariate choropleth maps help local decision makers to ensure that their decisions are made based on the statistical estimates with their uncertainty.

The maps in Figs. 5 and 7 indicate children living in north (particularly Netrokona and Kishoregonj districts) and north-eastern (particularly Sunamganj and Habiganj districts) regions are more vulnerable to stunting and underweight than the other regions of the country. For wasting, the spatial distribution shows that districts surrounding the capital city Dhaka have lower prevalence of wasting (Fig. 6). A higher wasting level represented by yellowish colour is scattered around the country. For example, Maulvibazar in Sylhet division, Sherpur in Mymensingh division, Lakxmipur in Chittagong division and Pirojpur in Barishal division. Spatial maps of stunting and underweight indicates high positive correlation among the north-eastern (higher prevalence) and the south-western (lower prevalence) districts, however the correlation seems moderate in the districts situated in central and south-eastern regions where vulnerability due to underweight is less compared to that for stunting. These estimates are also more precise, under model-based small area estimation.

The bivariate map in Fig. 5 shows a higher prevalence of stunting (≥30*%*) with higher SEs (≥2*%*) in north-eastern region, hilly districts of south-eastern region and in some southern districts. For underweight, there is higher prevalence of underweight (≥24.9*%*) with corresponding higher SEs (≥2*%*) in a number of districts belonging to the north, north-eastern and southern regions (Fig. 7). For both stunting and underweight, lower prevalence (≤25.9*%* for stunting and ≤21.2*%* for underweight) with higher accuracy is observed in most of the districts of south-western districts (Khulna division). The capital city Dhaka has higher stunting but lower underweight levels with higher accuracy and the port city of Chittagong (now known as Chattogram) has lower stunting and underweight level with higher accuracy, since both the districts have considerably larger sample sizes in the survey data (being large populous regions in Bangladesh). In case of wasting, Fig. 6 shows that many districts in the central region have lower prevalence (≤7*%*) with higher accuracy.

**Fig. 8:**
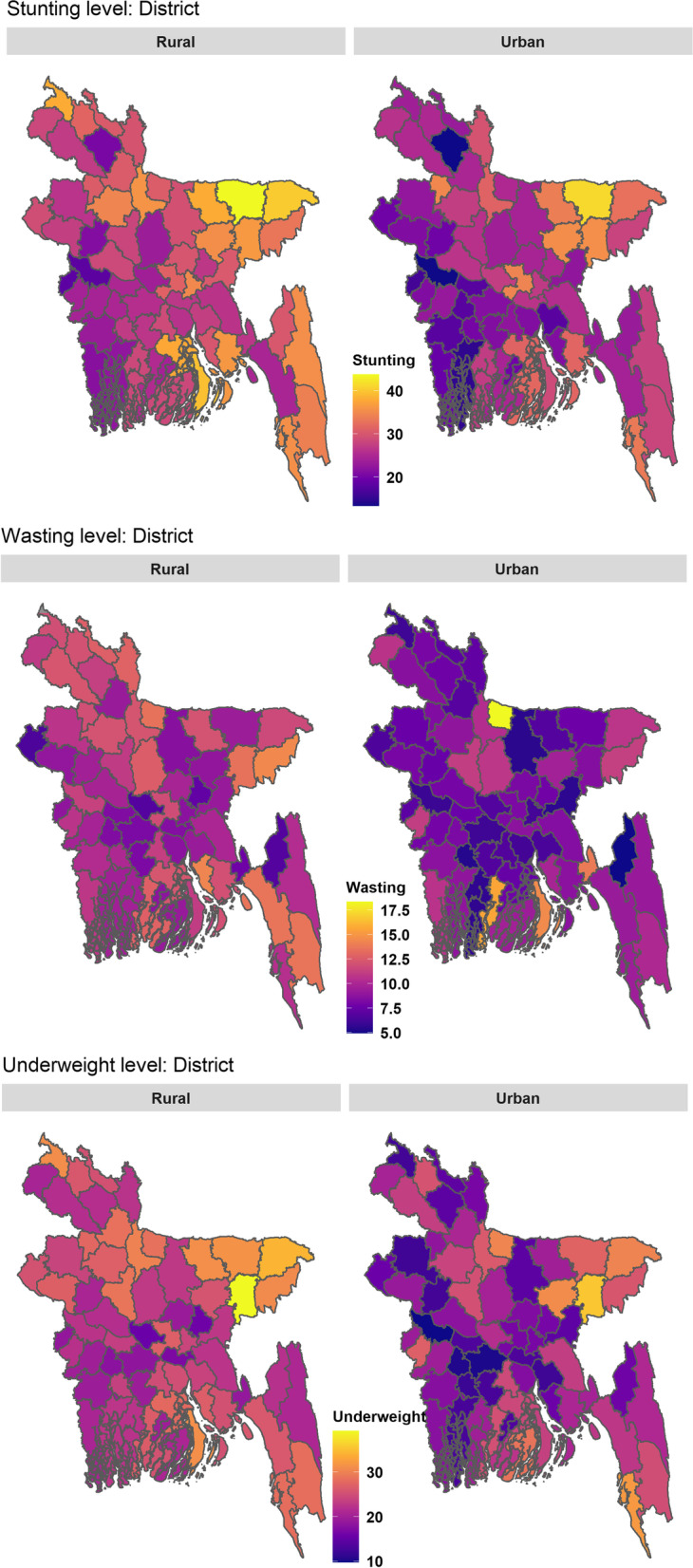
District level map for the prevalence of stunting, wasting and underweight by the place of residence

**Fig. 9:**
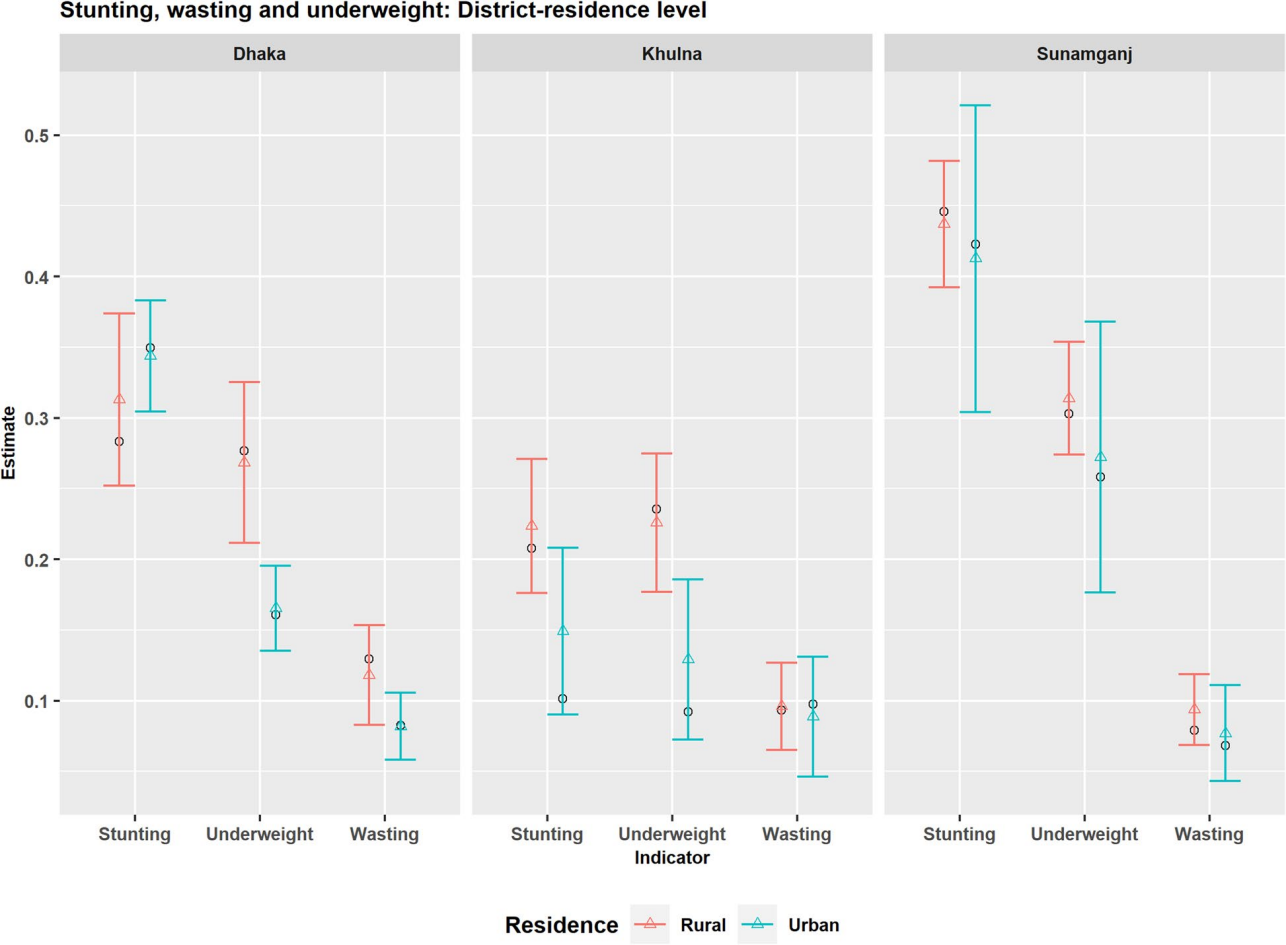
District level prevalence of stunting, wasting and underweight by the place of residence for Dhaka, Khulna and Sunamganj districts estimated by design-based (black circle dot) and model-based (coloured triangle dots) estimators (with 95% CI)

District level prevalence of children undernutrition indicators by place of residence are mapped in Fig. 8. The maps explicitly reveal higher rural-urban difference in wasting and underweight compared to stunting and higher heterogeneity in the spatial distribution among the urban domains than the rural domains as expected according to the estimated random effects variance parameters shown in Table 2. For all the indicators, urban domains of south-western districts seem the least vulnerable groups. To investigate in detail, the estimated prevalence by place of residence are plotted in Fig. 9 for Khulna, Dhaka, and Sunamganj districts, which belong to south-western, central and north-eastern regions and have different degrees of child undernutrition vulnerability. Figure 9 shows that prevalence of stunting is higher in the urban parts of Dhaka district (highly dense urban areas) though the prevalence is higher in most of the rural parts of Bangladesh. Interestingly, the precision for rural parts of Dhaka district is comparatively lower than that of urban parts only in Dhaka district. These patterns of estimate and precision are reversed in case of Khulna and Sunamganj districts, who have the lowest and highest prevalence of stunting respectively. In highly populated districts, the rural-urban difference in either stunting or underweight is found minimal. Some examples are the second highest populated district (Chittagong) and two other densely populated districts (Gazipur and Narayanganj) adjacent to Dhaka district (please see Figs. S.4 and S.5). The comparison of DIR and SAE estimates in Fig. 9 also shows that stunting and underweight levels are highly underestimated by the direct estimator for Khulna district. Model-based estimators overcome this underestimation which, in turn, reduces the rural-urban differences. Rural-urban difference in the prevalence of child undernutrition for other districts are shown in Supplementary Figs. S.3-S.9.

**Fig. 10:**
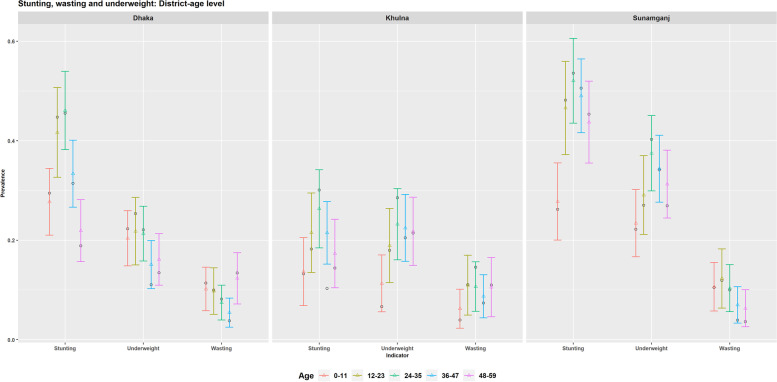
District level prevalence of stunting, wasting and underweight by the children age-groups for Dhaka, Khulna and Sunamganj districts estimated by design-based (black circle dot) and model-based (coloured triangle dots) estimators (with 95% CI)

Figure 10 shows that the trend of prevalence with the increase of children age remain same in the district level with significant differences by districts. Stunting level is found lowest in the oldest age-group instead of the infant age-group in Dhaka district, While stunting level for the older age-groups (more than 40%) is significantly far way from the level of infants (about 25%) in Sunamganj district. The underweight level decreases with the age-groups in Dhaka while the levels in Khulna and Sunamganj district follow the usual trend of increasing up to 24-35 months age group. In case of wasting, no significant difference is observed among the districts. Age-group specific prevalence level of stunting, wasting and underweight for other districts are shown in Supplementary Figs. S.11-S.17.

District level prevalence of undernutrition indicators by children age-groups are also mapped in Supplementary Fig. S.10. The maps show that the spatial pattern varies with the increase of children’s age for both stunting and underweight. On the one hand, for stunting, the map’s colour tends to move from deep purple for younger children to orange and light yellow colours for older children (specifically those aged 24-35 months). For underweight, this pattern change from purple to orange-yellow colours remain the same with increasing children age. On the other hand, for wasting, no spatial pattern is visible except some differences for the 12-24 age-group.

**Fig. 11:**
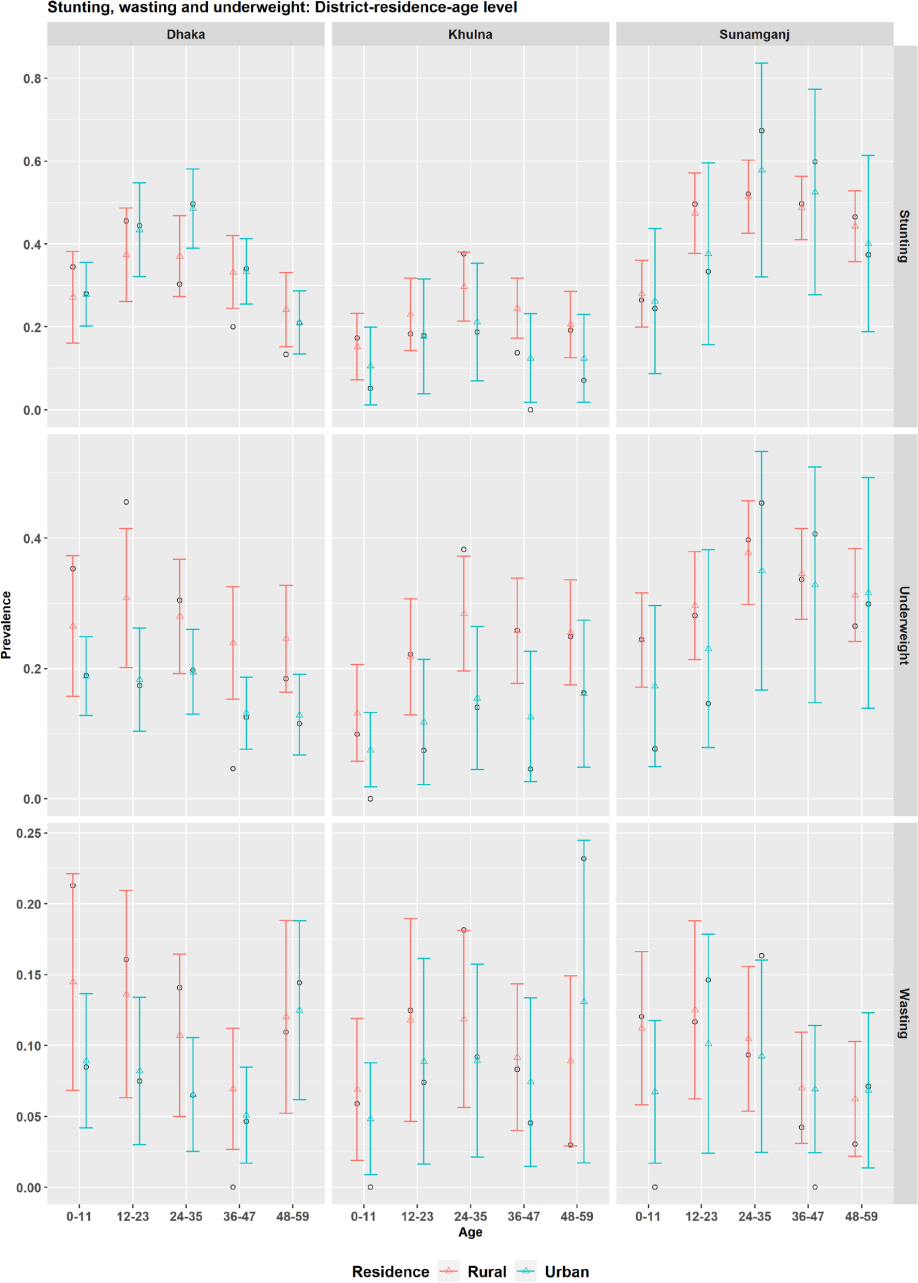
Prevalence of stunting, wasting and underweight for cross-classified domains of children age-groups and place of residence for Dhaka, Khulna and Sunamganj districts estimated by design-based (black circle dot) and model-based (coloured triangle dots) estimators (with 95% CI)

Instead of mapping the district level estimates of stunting, wasting and underweight for the corresponding cross-classified domains of children age-groups and place of residence, prevalence levels for three districts Dhaka, Khulna and Sunamganj are plotted in Fig. 11, to demonstrate the benefits of multilevel modelling. This figure reveals higher stunting levels for the urban children aged 12-23 and 24-35 months and negligible rural-urban difference for other age-groups lead to higher prevalence of stunting in urban parts of Dhaka district. However, considerable rural-urban differences are observed in case of underweight and wasting for all age-groups in Dhaka district. In Khulna district, considerable rural-urban differences exist for most of the age-groups (except 48-59 group for wasting), however these difference seem negligible in the case of Sunamganj district. Confidence bands of the estimates indicate that estimates for rural domains of Dhaka district and urban domains of Khulna and Sunamganj districts are less precise. These findings are also supported by the fact that the direct estimates (black circle), particularly for many rural domains of Dhaka district and urban domains of Khulna and Sunamganj districts, are unreasonably very high or low compared to the expected level with respect to the corresponding age-group and residence (lying outside the SAE confidence bands). The differences in the prevalence level of stunting, wasting and underweight by children age-group and place of residence for other districts are shown in Supplementary Figs. S.18-S.25.

**Fig. 12:**
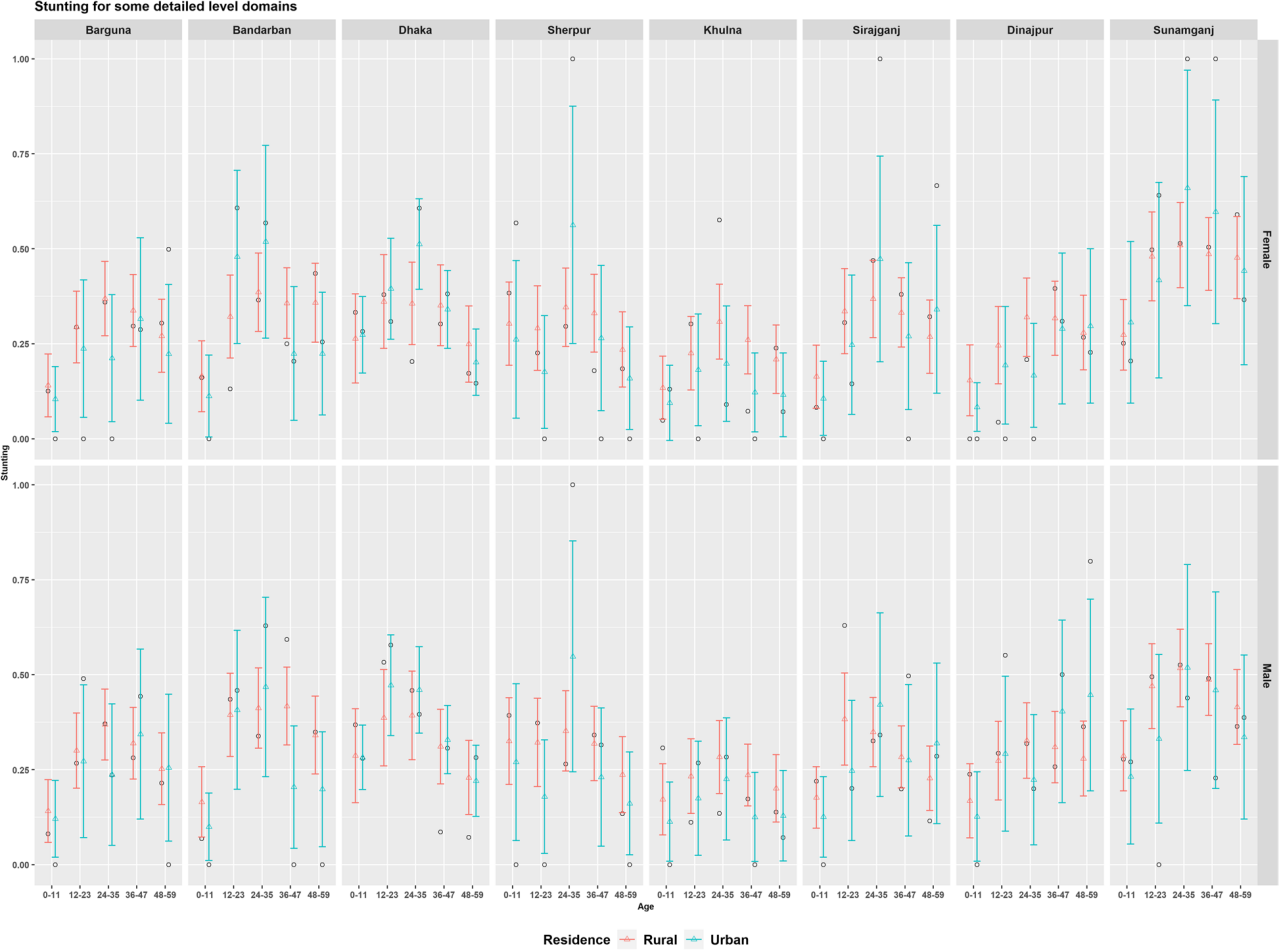
Prevalence of stunting for the detailed level domains of 8 districts as representative of 8 divisions estimated by design-based (black circle dot) and model-based (triangle coloured dots) estimators (with 95% CI)

The estimated stunting level for the detailed level domains of district-residence-age-sex shown in Fig. 12 shows some sex differences particularly for the 12-23 and 24-35 months age-groups. These differences mainly arise in the direct estimates for sample size differences particularly for the urban domains, and SAE estimators overcome these differences. Figure 12 shows considerable gender-inequality in the prevalence of stunting for the domains of Sunamganj district. These rural-urban differences also show by gender in some districts of Rajshahi (see Joypurhat and Naogaon in Supplementary Fig. S.30 and Rangpur (see Lalmonirhat and Panchagarh in Supplementary Fig. S.31) divisions. Such gender inequality is also observed in the case of wasting (for example, Sherpur district in the north) and underweight (for example, Dinajpur district in the north-west) shown in Supplementary Figs. S.34 and S.43 respectively. The prevalence of stunting, wasting and underweight at the most detailed level for all districts are shown by division in the Supplementary file from Figs. S.26 to S.42.

## Discussion

In this study, multilevel models expressed in a Bayesian framework have been developed for child malnutrition indicators of stunting, wasting and underweight in Bangladesh for spatio-demographic cross-classified small domains of sixty-four districts, two places of residence (urban and rural), children sex and their five age-groups. The developed multilevel models have allowed for cross-sectional and spatial correlations that increase the effective sample size of the detailed level domains through specification of various fixed effects and random effects of the cross-classified variables of district (*district*), place of residence (*residence*), sex of child (*sex*) and age-group (*age*) along with the sub-national variable (*division*). All the three finally selected multilevel models have common fixed effects components, which consist of only the main effects of *sex*, *age*, *residence* and *division*. The interaction effects of the demographic variables with administrative units are allocated as random effects in the model (to account for the unobserved differences that exist at the district and division levels).

After accounting for the district and division level random effects, the fixed effects components indicate that children aged 24-35 months, living in Sylhet division and rural domains have higher possibility of being stunted and underweight. These patterns are expected, based on existing studies which have explored risk factors of children undernutrition in Bangladesh [16, 24, 50]. For wasting, most of the fixed effects are found insignificant except for sex (male domains have slightly higher prevalence) and children age-group (12-23 months age-groups has lower prevalence than infants). In some studies [51, 52], male children were found to have higher risk of being wasted. However, infants were found to have lower risk of wasting than other children in older age-groups. This is reflected in findings in most studies. Due to having inconsistent direct estimates for the urban domains compared to rural domains, the rural-urban variations are found significant at various disaggregation levels, such as district-residence, district-residence-sex, and district-residence-age. These variations are accounted for by specifying random effects for the respective detailed level domains with unequal variance components for rural and urban domains. In a similar way, to account for age-specific variation at the district level, random regression coefficients for children age-groups are assumed to vary over districts with diagonal variance structure (i.e. with zero correlation between age-groups). The correlation between the districts are mainly captured by the spatial random effects component, which borrow spatial strength in the model from the neighbouring districts.

District level variation for 0-11 and 12-23 months age-groups are found significant for all models, while the variation for 48-59 months age-group is found significant for stunting and underweight. The spatial component had less contribution for wasting and so it was removed from the finally selected model of wasting. The spatial distribution of stunting, wasting and underweight at the district (Figs. 5, 6, 7) and district-residence (Fig. 8) levels also suggest that spatial correlation among the districts are stronger for stunting and underweight compared to wasting. In addition to this random effects components, two white noise components are added finally to account for unexplained (residual) variation at the detailed level domains of district (district-residence-age-sex) and division (division-residence-age-sex).

The direct estimates at the detailed level were too volatile and inconsistent, and particularly for wasting there were many domains with zero (and also one) estimates as well as zero standard errors. The developed multilevel models provide reasonable estimates with better accuracy for these domains with zero and one estimates. Model assessment through four model discrepancy measures (relative bias, absolute relative bias, relative reduction of standard errors and ratio of coefficient of variations) confirms that multilevel model-based estimator provides approximately unbiased and more consistent estimate for most of the cross-classified domains of division and district. The aggregation from the very detailed level to the national level also confirms that the model-based estimates are consistent to the direct estimates at the various higher aggregation levels such as national, division, residence, children sex and age-class.

The national-level prevalence of stunting and wasting have been declined in 2019, however these levels are still within the label of “high” prevalence (20– <30% for stunting and 10– <15% for wasting) according to the recent WHO threshold of stunting and wasting [6]. The level of underweight is also high (20– <30%) according to old referenced WHO threshold of underweight [53]. Division level prevalence indicate that only the Khulna division reached to the SDG target of around 20% stunting level, while wasting level is almost double of 5% for all the divisions. Children of Sylhet division are highly vulnerable to all the undernutrition indicators, followed by the children of Mymensingh and Barishal divisions. Vulnerability scenarios differ only in Dhaka division, where children are more stunted compared to the underweight and wasting levels. This different scenario is attributed to the densely populated capital district Dhaka where stunting level is higher among the urban children than the rural children. Though the rural-urban difference at the national level is lower for all the indicators, the difference increases with the disaggregation for many domains.

Children aged more than one year particularly 12-23 and 24-35 months old are highly vulnerable to stunting and underweight compared to infants. However, more than one-quarter infants of Sylhet and Mymensingh divisions are stunted. On the other hand, infants are prone to loss their weight very quickly, however the wasting level does not vary too much by children age-groups, but their cross-classification with sex and place of residence indicate consistent rural-urban difference over all age-group and male children have higher wasting level. For underweight, the rural-urban difference increase with children age-groups and rural females are seemed to be more vulnerable than rural males. These age-sex-residence specific differences vary considerably with different degrees at the division level. As for example, urban children of Dhaka division have slightly higher stunting level for most of the age-groups, which is opposite to other divisions. On the other hand, rural-urban differences in underweight and wasting are considerably higher in less populated divisions, particularly for the female children.

Spatial distributions of district level stunting and underweight indicate that children in north and north-eastern districts are highly vulnerable to stunting and underweight compared to those districts in south-western districts, while children in the central region are more vulnerable to stunting than underweight and wasting. The spatial patterns of stunting also reveal that the level started to increase with the move from the south-western region to the north-eastern region through the central and north regions of the country. According to the bivariate choropleth maps, the estimates of stunting and underweight are more accurate in the south-western and central regions. It may happen due to two reasons, either the sample size or the magnitude of the estimates. The estimates for the districts under Sylhet division (smaller sample size with higher estimates) are found with higher standard errors compared to those of in Khulna (smaller sample size with lower estimate) and Dhaka (bigger sample size with moderate estimate) divisions. The choropleth map of wasting suggests districts with higher wasting level with higher standard errors are sparsely distributed across the country.

District level spatial distributions by place of residence are expected to have higher heterogeneity among the urban parts of districts compared to their rural counterparts, since urban-specific variance components were considerably higher than those for rural parts in the model development. All the undernutrition indicators are higher in most of rural parts of districts with higher precision except in the Dhaka district. Only in Dhaka district, stunting level for urban part is slightly higher with higher precision than rural part. The rural-urban difference become wider in the district level (Kushtia, Narail, Khulna, Netrokona, Nawabganj, and Panchagarh are some example) compared to the division level as expected, however when urban domains have higher prevalence, then the rural-urban difference is minimal particularly in case of stunting and underweight (Comilla, Dhaka, Patuakhali, Kishoreganj, Manikganj, Sherpur, and Joypurhat are some example). In the highly urbanized districts (like Dhaka, Chittagong, Gazipur, and Narayanganj), the rural-urban difference are lower, which may be due to having many slums in the densely populated city areas [54, 55].

District level spatial distributions by children age indicate stunting and underweight levels are about 10-15% higher among the older age-groups (particularly 12-23 and 24-35 months) than the infants, and these scenario is more prevalent in most of the districts belonging to vulnerable Sylhet, Mymensingh and Barishal divisions. In the Khulna division, the differences in the prevalence of stunting and underweight between infants and younger age-groups are also persistent but the levels for the infants are around 15% for most of the districts. So significant inequalities in the prevalence of stunting and underweight are mainly due to the infants and the other older age-groups. Since age-class has lower influence in the prevalence of wasting, modeling wasting is highly complex than the stunting and underweight.

In case of cross-classified domains of districts with children age-group and residence, the rural-urban difference has increased as expected but the patterns of rural-urban differences are very similar to those at the district-by-residence level for each of the age-groups. These patterns remain similar when disaggregated down to district-residence-age-sex. However, the direct estimates at these detailed cross-classified domains are too volatile and model-based estimators have reduced this volatility.

Our study has some limitations. Concurrence of stunting, wasting and underweight may lead to correlation in between their prevalence level at various aggregation levels [56]. Developing univariate models for each of the undernutrition indicators by ignoring these correlations is one of the limitations of this study. A multivariate small area model would bring additional strength from the cross-correlation among the three indicators [22, 57]. However, the multivariate multilevel model at the considered detailed level would require survey-weighted co-variance matrix of dimension 3×3 for each of the detailed level 1280 domains. Due to having small sample size, estimation of consistent covariances would be more complex for many domains. In addition, the unavailability of Z-scores for the three indicators for all children would lead to loss of information for a significant proportion of domains. Most importantly, the estimation of a multivariate multilevel model would be more complex, and time and computationally intensive.

Nonetheless, the main contribution of this study is the prediction of child undernutriton indicators at various disaggregation levels of division and district by developing models at the detailed cross-classified domains of children sex, age, place of residence and district by accounting variation in the target outcomes at the detailed as well as higher aggregation levels. The considered multilevel modelling approach helped to provide reliable and consistent official statistics as well as to explore the administrative hierarchies and demographic factors for which inequalities in the undernutrition vulnerability are still persistent in Bangladesh. Additionally, the bivariate choropleth map might help the policymakers to take their decisions based on the estimates as well as their quality.

## Supplementary Information

**Additional file 1.** We added a Supplementary file where detailed level results in terms of figures and tables are given for review purpose.

## Data Availability

The microdata of 2019 Bangladesh MICS are publicly available from the MICS website. And the micro-data of census 2011 is also available from the IPUMS International website. The extracted input variables from survey and census data have been submitted to this journal as additional supporting file.
