# Supplementary material for: Prevalence of child undernutrition measures and their spatio-demographic inequalities in Bangladesh: an application of multilevel Bayesian modelling
Source: BMC Public Health. 2022 May 18;22:1008. doi: 10.1186/s12889-022-13170-4 (PMC9118603; doi:10.1186/s12889-022-13170-4)
Supplement: Supplementary file 1 — Additional file 1. We added a Supplementary file where detailed level results in terms of figures and tables are given for review purpose. [file 12889_2022_13170_MOESM1_ESM.pdf]

Supplementary Materials

Title: Prevalence of child undernutrition measures and their spatio-demographic inequalities in Bangladesh: An application of multilevel Bayesian modelling

Authors: Sumonkanti Das[1*], Bernard Baffour[1], Alice Richardson[2]
[1] School of Demography, Australian National University, Canberra, ACT, Australia
[2] Statistical Support Network, Australian National University, Canberra, ACT, Australia
* Corresponding Author E-mail: sumonaknti.das@anu.edu.au

The recent MICS conducted in 2019 covered all 64 districts in the survey design for identifying localised disparities as well as the most vulnerable for social inclusion [1]. However, district level estimates of three undernutrition indicators have remained largely unexplored and widely unpublished. Availability of the large dataset has given scope to investigate the disparities in the prevalence of children undernutrition at district level as well as at the corresponding spatio-demographic detailed level domains. So this study aims to estimate the prevalence of stunting, wasting and underweight for 1280 small domains which are cross-classified by children's sex (female and male), five age-groups (0-11, 12-23, 24-35, 36-47, and 48-59 months), place of residence (rural and urban) and 64 districts by applying an extension of small area estimation method using multilevel modelling. This approach has explored spatio-demographic variation at both division and district levels (first and second administrative units respectively). These disaggregated level prevalence of children undernutrition indicators as well as the observed spatio-demographic inequalities might help policy makers to understand the spatial disparity in the undernutrition vulnerability and how the vulnerability is distributed unevenly throughout Bangladesh. To the best of our knowledge, such investigation on the dynamics of spatio-demographic disparities in child undernutriton has not been yet done in Bangladesh. So we expect this understanding will provide immense benefit for policy-making in the race of meeting SDG goals by 2030.

S.1 Data inputs

In the Bangladesh Multiple Indicator Cluster Survey (MICS) 2019, a total of 24,686 children under 5 years of age were recorded for measuring weights and heights to calculate their anthropometric indices for height-for-age, weight-for-age and weight-for-height expressed in standard deviation units (z-scores) from the median of the reference population. According to MICS 2019 report, about 2.8%, 4.5%, and 4.7% children were excluded from calculations of the weight-for-age, height-for-age, and weight-for-height indicators, respectively due to either missing weight and height measurements or measurements were out of normal range [1].

Though the MICS 2019 provides a large data set representative at the district level, the sample size of the targeted cross-classified domains are comparatively very small to estimate undernutrition indicators with enough precision. The sample size for the rural sampled domains varies over about 11-60 with a mean of about 28, while for the urban domains it ranges between 1-66 with a mean of 6.5. Notably, there are about 14 domains with no observation for all the three indicators. The comparison of domain-specific sample size with their respective population size obtained from Census 2011 (shown in Figure S.1 in Supplementary file) also indicates sample size for most of the urban domains are comparatively very small than those of rural domains having under five children below 10,000. Consequently, it is expected the estimates of child undernutrition will be comparatively highly inconsistent for the urban domains.

Distribution of the number of children in the census population and the survey data set for the 1280 cross-classified domains of 64 districts, rural-urban place of residence, children's sex and 5 age-groups (0-11, 12-23, 24-35, 36-47, and 48-59 months) are plotted in Figure S.1 in case of stunting only. These distributions for wasting and underweight are very similar.

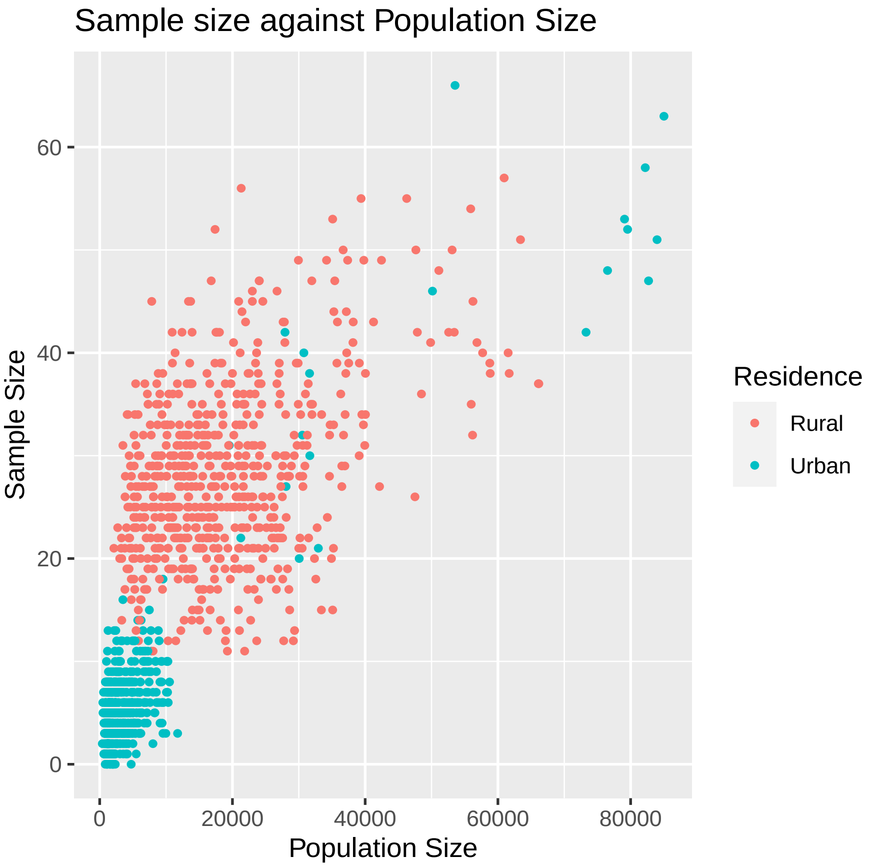

Fig. S.1 Sample size against population size by place of residence for the 1280 cross-classified domains of district, residence, children age and sex.

S.2 Generalized variance function (GVF) model

The generalized variance function (GVF) model developed under square-root scale of standard errors are shown in Table S.1, Table S.2, and S.3 for stunting, wasting and underweight respectively. The GVF smoothed standard errors are obtained with small bias correction by adding σ^2 with the predicted smoothed sampling error variances.

Variable	Estimate	Std. Error	t value	Pr(> $\|t\|$)
(Intercept)	0.580	0.018	32.149	0.000
$\sqrt{(\hat{y_d})}$	-0.146	0.018	-8.038	0.000
$\sqrt{(n+1)}$	-0.036	0.002	-18.771	0.000
Chattogram	0.011	0.008	1.354	0.176
Dhaka	0.001	0.008	0.088	0.930
Khulna	-0.001	0.008	-0.085	0.932
Mymensingh	0.000	0.010	0.007	0.994
Rajshahi	-0.003	0.008	-0.348	0.728
Rangpur	0.004	0.008	0.467	0.641
Sylhet	0.019	0.010	1.898	0.058
Urban	0.032	0.011	2.808	0.005
Chattogram:Urban	-0.026	0.012	-2.133	0.033
Dhaka:Urban	-0.007	0.012	-0.564	0.573
Khulna:Urban	-0.006	0.013	-0.441	0.660
Mymensingh:Urban	0.017	0.016	1.062	0.289
Rajshahi:Urban	0.007	0.013	0.552	0.581
Rangpur:Urban	-0.025	0.013	-1.913	0.056
Sylhet:Urban	-0.014	0.015	-0.930	0.353
Adjusted $R^2 = 62.58\%$; $\sigma^2 = 0.0023$				

Table S.1 Regression coefficients of the generalized variance function (GVF) model for the standard errors (SE) of stunting. The GVF model is fitted on square-root scale of the SEs.

S.3 Multilevel Bayesian model formation

Under the considered multilevel Bayesian model, the fixed effects parameters are assumed to follow a weakly informative prior distributions as $p(\beta) = \mathcal{N}(0, 100I)$. The scaled-inverse Wishart and half-Cauchy priors are used for the standard deviation parameters when unstructured [2] and diagonal [3] covariance structure are assumed respectively.

A number of multilevel models structured as (1) in the main text were fitted using Markov Chain Monte Carlo simulation method for each of the undernutrition indicators in R [4] using package `mcmcsae` [5]. Two model performance measures, namely the Widely Applicable Information Criterion or Watanabe-Akaike Information Criterion (WAIC) [6, 7] and the Deviance Information Criterion (DIC) [8] are used to compare models developed with same input estimates. The selected best multilevel model for each of the indicators

Variable	Estimate	Std. Error	t value	Pr(> $\|t\|$)
(Intercept)	0.497	0.013	38.905	0.000
$\sqrt(\hat{y_d})$	-0.031	0.015	-2.099	0.036
$\sqrt(n+1)$	-0.035	0.002	-19.920	0.000
Chattogram	0.002	0.007	0.372	0.710
Dhaka	0.000	0.006	0.033	0.974
Khulna	-0.001	0.007	-0.103	0.918
Mymensingh	-0.001	0.008	-0.151	0.880
Rajshahi	-0.003	0.007	-0.362	0.718
Rangpur	0.001	0.007	0.127	0.899
Sylhet	0.018	0.008	2.117	0.035
Urban	0.043	0.011	3.967	0.000
Chattogram:Urban	-0.017	0.011	-1.510	0.131
Dhaka:Urban	-0.001	0.012	-0.062	0.951
Khulna:Urban	-0.009	0.012	-0.707	0.480
Mymensingh:Urban	0.001	0.016	0.058	0.954
Rajshahi:Urban	0.008	0.013	0.627	0.531
Rangpur:Urban	-0.028	0.013	-2.123	0.034
Sylhet:Urban	-0.020	0.015	-1.364	0.173
Adjusted $R^2 = 65.9\%$; $\sigma^2 = 0.0015$				

Table S.2 Regression coefficients of the generalized variance function (GVF) model for the standard errors (SE) of wasting. The GVF model is fitted on square-root scale of the SEs.

was run finally with 2000 iterations so that longer simulations provide the Gelman-Rubin potential scale reduction factor below the recommended 1.10 value for all model parameters and model predictions at convergence [9].

Variable	Estimate	Std. Error	t value	Pr(> $\|t\|$)
(Intercept)	0.563	0.018	31.779	0.000
$\sqrt(\hat{y_d})$	-0.120	0.018	-6.640	0.000
$\sqrt(n+1)$	-0.035	0.002	-19.616	0.000
Chattogram	0.006	0.007	0.789	0.430
Dhaka	-0.004	0.007	-0.592	0.554
Khulna	-0.004	0.008	-0.566	0.572
Mymensingh	0.002	0.009	0.160	0.873
Rajshahi	-0.002	0.008	-0.271	0.786
Rangpur	-0.000	0.008	-0.054	0.957
Sylhet	0.012	0.010	1.230	0.219
Urban	0.043	0.011	3.967	0.000
Chattogram:Urban	-0.034	0.012	-2.850	0.004
Dhaka:Urban	-0.015	0.012	-1.222	0.222
Khulna:Urban	-0.009	0.013	-0.698	0.485
Mymensingh:Urban	-0.006	0.015	-0.394	0.694
Rajshahi:Urban	-0.012	0.013	-0.939	0.348
Rangpur:Urban	-0.025	0.013	-1.944	0.052
Sylhet:Urban	-0.021	0.015	-1.446	0.149
Adjusted $R^2 = 65.62\%$; $\sigma^2 = 0.0021$				

Table S.3 Regression coefficients of the generalized variance function (GVF) model for the standard errors (SE) of underweight. The GVF model is fitted on square-root scale of the SEs.

S.4 Model assessment

Information criteria the Widely Applicable Information Criterion or Watanabe-Akaike Information Criterion (WAIC) [6, 7] and the Deviance Information Criterion (DIC) [8] were utilized to find the best model among the models developed with same input estimates for each of the three undernutrition indicators. As model diagnostics, however, four discrepancy measures (i) relative bias (RB), (ii) absolute relative bias (ARB), (iii) relative reduction of the standard errors (RRSE), and the ratio of coefficients of variation (CV) denoted by CVR are calculated by comparing the model-based estimates denoted by $\hat{\theta}_d$ with the corresponding direct estimates $\hat{Y}_d$ for domain d to evaluate how the multilevel models perform. Hereafter model-based and design-based estimates are denoted by SAE and DIR respectively. The RB and ARB defined by $RB_d = (\hat{\theta}_d - \hat{Y}_d)/\hat{Y}_d$ and $ARB_d =\mid \hat{\theta}_d - \hat{Y}_d \mid /\hat{Y}_d$ indicates how much SAE estimates deviates from DIR estimates relative to the later one. The third measure RRSE defined by $(se(\hat{Y}_d) - se(\hat{\theta}_d))/se(\hat{Y}_d)$ (where $se(\hat{\theta}_d)$ is SE of $\hat{\theta}_d$) indicates how much accuracy has gained by the SAE estimates compared to the DIR estimates relative to the SAE estimates. At the most disaggregated level, the values of RRSE are expected to be higher than those at the higher aggregation level due to a greater chance of small (and zero) samples. The fourth measure CVR helps to examine the relative SEs of SAE estimates, their CVs are compared to those of DIR estimates. The values of CVR less than 100% indicates that the SAE estimates provide better relative accuracy than the DIR estimates. All these measures are expressed in percentage in Table S.4. The measures are calculated at different (i.e., higher and lower) aggregation levels such as division, district, division×age, district×age, district×age×residence, and district×age×residence×sex.

$$\begin{aligned} RB_{it} &= \frac{(\hat{\theta}_{it} - \hat{Y}_{it})}{\hat{Y}_{it}} \times 100\% \\ ARB_{it} &= \frac{_{it} - \hat{Y}_{it}}{\hat{Y}_{it}} \times 100\% \\ RRSE_{it} &= 100\% \times (se(\hat{Y}_{it}) - se(\hat{\theta}_{it}))/se(\hat{Y}_{it})\,. \end{aligned} \tag{1}$$

For all the indicators, it is observed that, on one hand, the values of RB, ARB and RRSE increase with disaggregation levels, while the CV ratios decrease. These are expected since the direct estimates are more reliable and consistent at the higher aggregation level than the disaggregation level. On the other hand, the SAE estimates are as consistent as the DIR estimates at the higher aggregation level (there is little difference between the measures when compared to the direct estimates). More importantly, however, at the disaggregated level SAE estimates are more accurate than the DIR estimates. The SAE estimates appear to provide more gains for the cross-classified domains of district. The highest gains, in terms of RRSE and CVR, are observed at the most detailed level at which the multilevel models are developed. Figure S.2

in Supplementary file also supports that the developed multilevel models provide approximately unbiased and consistent estimates with reasonably better CVs at the most detailed level, particularly for the urban domains. This does make intuitive sense since it highlights the importance of the model estimation having more gains at the detailed disaggregated level.

At higher aggregation levels, the values of RB and ARB are found slightly higher for stunting and underweight compared to wasting, which indicates that the SAE estimates provide slightly higher estimates than the DIR estimates (only about 3% of the DIR estimates). The opposite pattern of ARB, at the detailed level, indicates that the SAE estimates are slightly overestimated for wasting than stunting and underweight. The purported reason for this is that the DIR estimates of wasting for many detailed level urban domains are too volatile shown in Figure S.2. Figure S.2 also shows how the developed multilevel models provide reasonable estimates for the domains with zero and one DIR estimates as well as zero sample size. For these domains, the huge uncertainty mean that the SAE estimates greatly improve upon the DIR estimates through using cross-sectional and spatial information from other domains.

At the detailed level, the multilevel model-based SAE estimator is expected to outperform the DIR estimator in terms of RRSE and CVR shown in Table S.4. To show how the models provide reliable estimates by bringing strength from similar domains and spatial location, the SAE estimates of stunting (with 95% CI) for the detailed level domains under eight districts (Barguna, Bandarban, Dhaka, Sherpur, Khulna, Sirajganj, Dinajpur, and Sunamganj) as the representatives of eight divisions as well as some data characteristics such as domains with zero (and one) estimates, and the degree of undernutrition vulnerability. Model-based estimates with their 95% CI (coloured dots and error-bar lines) are plotted along with the DIR estimates (black circular dots) in Figure **??**. All the considered districts except Dhaka have domains with zero estimates, where SAE estimators provide reasonable estimates. Some districts (Sherpur, Satkhira, Sirajganj, and Sunamganj) have one or more urban domains with the DIR estimates of one (sample size ranges 1-2 for most cases). The developed multilevel model improves on these inconsistent estimates and provided reasonable estimates by bringing strength from the relevant domains, particularly in the highly vulnerable age-groups 12-23 and 24-35 months. The SAE estimates follow the trend of comparatively more consistent DIR estimates, as for example most of the urban domains of Dhaka district. The performance of the developed multilevel models for wasting and underweight are very similar and so the similar comparisons for all the indicators are shown by division in Supplementary Figures from S.26 to S.51.

Indicator	Stunting				Wasting				Underweight			
Aggregation level	RB	ARB	RRSE	CVR	RB	ARB	RRSE	CVR	RB	ARB	RRSE	CVR
Nation	3.8	3.8	-1.6	97.8	1.6	1.6	4.4	94.1	3.5	3.5	0.4	96.2
Division	3.9	3.9	-0.7	96.9	2.1	2.3	6.9	91.2	3.6	3.6	0.2	96.3
District	5.3	6.3	9.4	86.2	4.6	9.4	19.5	77.4	5.4	6.4	10.3	85.4
Residence	3.9	3.9	-0.2	96.4	-0.3	3.0	7.1	93.2	3.6	3.6	1.2	95.3
Sex	3.8	3.8	1.7	94.7	1.6	1.6	7.9	90.6	3.5	3.5	2.4	94.3
Age	4.1	4.1	2.3	94.0	1.7	1.8	8.1	90.5	3.7	3.7	2.8	93.7
Division×Residence	5.5	5.5	7.0	88.3	-0.2	5.1	18.1	82.0	4.2	4.2	6.3	90.1
Division×Sex	4.0	4.1	12.5	84.2	2.3	3.0	18.9	79.4	3.6	3.8	12.8	84.2
Division×Age	4.9	5.6	14.6	81.6	3.1	5.7	19.9	78.0	4.2	5.1	17.3	79.4
District×Residence	8.6	11.0	16.1	78.4	13.1	23.7	29.8	67.4	10.8	14.0	15.1	79.3
District×Sex	6.0	8.8	26.6	69.7	6.3	13.5	32.0	65.0	6.3	9.6	24.7	71.4
District×Age	10.8	16.2	25.6	68.7	17.8	28.5	26.2	66.6	11.9	18.2	26.2	67.8
District×Residence×Sex	8.6	11.0	16.1	78.4	13.1	23.7	29.8	67.4	10.8	14.0	15.1	79.3
District×Residence×Age	11.9	21.8	27.2	67.7	7.2	31.8	36.1	63.6	11.7	24.7	31.3	64.7
District×Residence×Age×Sex	15.3	32.4	40.5	56.7	8.1	39.4	42.4	58.8	12.8	32.8	40.3	58.1

Table S.4 Mean of relative bias (MRB, in %), mean of relative absolute bias (MARB, in %), mean of relative reduction of the standard errors (MRRSE, in %), and mean of CV ratios (CVR, in %) at different aggregation levels for the multilevel models of stunting, wasting and underweight.

S.5 District level prevalence of child undernutrition

The performance of model-based estimators (SAE) at the detailed level has been examined by plotting the prevalence level, standard errors (SE) and coefficient of variation (CV%) against those estimated by the design-based direct estimators (DIR) in Figure S.2 for all the three indicators stunting, wasting and underweight. Summary statistics of model-based estimates of stunting, wasting and underweight at the district (D), district-sex (DS), district-age (DA), district-residence (DR), district-residence-sex (DRS), district-residence-age (DRA), and district-residence-age-sex (DRA) along with their CVs are shown in Table S.5.

District level prevalence of stunting, wasting and underweight estimated by design-based (DIR) and model-based (SAE) estimators along with the corresponding SEs and CVs expressed in percentage are shown in Table S.6, Table S.7, and Table S.8 respectively.

Level	Estimate (%)						CV (%)					
	Min	Q1	Median	Mean	Q3	Max	Min	Q1	Median	Mean	Q3	Max
Stunting												
D	17.6	25.4	28.2	28.5	32.7	43.4	4.9	7.0	7.8	7.8	8.6	10.8
DS	17.5	25.2	27.9	28.5	32.1	44.2	5.7	8.0	8.8	8.9	9.7	12.0
DA	10.1	22.4	28.7	28.5	33.9	52.1	7.2	11.5	13.7	14.7	16.7	28.8
DR	13.3	23.2	26.6	27.1	30.6	43.7	5.1	8.2	11.1	12.4	16.6	21.1
DRS	13.1	22.8	26.8	27.1	31.1	46.5	5.9	9.2	12.5	15.1	21.0	27.3
DRA	9.0	20.3	26.8	27.0	33.2	57.8	7.2	13.9	24.0	24.7	34.6	45.3
DRAS	8.4	19.9	26.7	27.1	33.4	65.9	8.0	16.2	26.7	27.2	37.2	53.2
Wasting												
D	6.3	8.8	10.1	10.2	11.5	14.1	8.5	11.9	12.7	12.8	13.6	17.9
DS	5.8	8.6	10.1	10.1	11.2	14.9	10.6	14.2	15.2	15.2	16.1	20.4
DA	3.4	8.0	10.1	10.2	11.9	25.2	15.7	23.0	24.8	25.4	27.3	37.4
DR	4.9	7.8	9.2	9.5	11.1	18.3	9.5	14.0	18.4	18.2	22.7	27.3
DRS	4.7	7.6	9.3	9.5	11.3	24.1	11.4	16.5	21.1	22.5	29.0	34.6
DRA	3.0	7.0	9.0	9.6	11.6	28.6	17.5	26.6	32.3	32.3	37.7	56.8
DRAS	2.5	6.9	8.9	9.6	11.6	32.3	19.9	32.3	37.9	38.2	44.2	61.2
Underweight												
D	16.1	20.5	22.6	23.5	26.2	38.7	5.6	7.8	8.8	8.7	9.6	12.1
DS	15.7	20.2	22.7	23.5	26.1	39.6	6.7	9.3	10.4	10.2	11.2	13.7
DA	10.4	19.5	23.0	23.5	27.4	44.6	9.3	13.3	14.9	16.0	17.9	30.5
DR	9.8	18.2	22.2	22.0	25.8	39.1	5.9	9.4	12.5	14.6	20.1	24.4
DRS	8.8	17.9	22.2	22.0	26.1	40.5	7.0	11.0	14.9	18.4	26.1	31.7
DRA	7.5	16.3	22.0	22.0	26.7	47.1	9.7	15.7	25.6	25.4	34.2	44.9
DRAS	7.1	16.2	21.8	22.0	26.9	54.0	11.5	19.3	29.3	29.5	39.5	52.4

Table S.5 Summary statistics of estimated stunting, wasting and underweight (in %) with their coefficient of variation CV (in %) for disaggregation level of district (**D**), district-sex (**DS**), district-age (**DA**), district-residence (**DR**), district-residence-sex (**DRS**), district-residence-age (**DRA**), and district-residence-age-sex (**DRAS**).

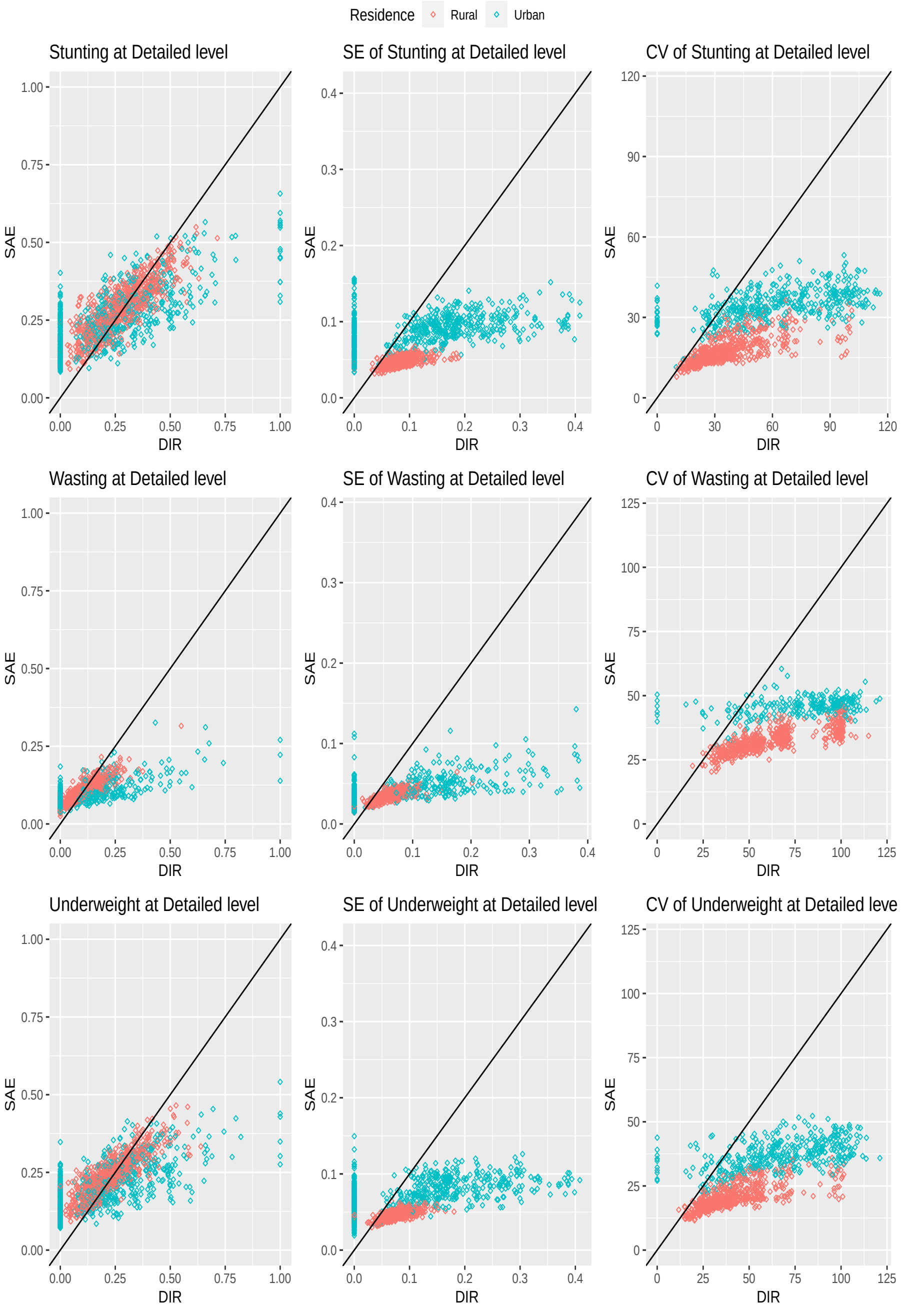

Fig. S.2 Comparison of estimates, standard errors (SE) and coefficient of variation (CV) obtained by the direct (DIR) and model-based (SAE) estimators for stunting, wasting and underweight at the detailed level 1280 domains.

No.	District	DIR			SAE		
		stunting	se	CV	stunting	se	CV
1	Barguna	26.6	2.4	8.9	28.4	2.3	8.0
2	Barisal	36.2	2.7	7.5	36.2	2.7	7.3
3	Bhola	38.5	2.2	5.7	38.3	2.0	5.3
4	Jhalokati	20.6	2.3	11.1	23.4	2.1	8.8
5	Patuakhali	28.4	2.3	8.1	28.9	2.1	7.4
6	Pirojpur	21.8	2.2	10.1	25.3	2.2	8.7
7	Bandarban	31.5	2.6	8.3	33.1	2.3	6.8
8	Brahmanbaria	27.4	2.1	7.6	28.9	2.0	6.9
9	Chandpur	23.4	2.4	10.4	25.8	2.2	8.4
10	Chittagong	23.4	1.5	6.6	24.5	1.4	5.9
11	Comilla	25.0	2.0	7.8	26.3	1.7	6.6
12	Cox's Bazar	34.6	2.3	6.8	35.0	2.3	6.7
13	Feni	24.0	2.0	8.4	25.8	2.0	7.7
14	Khagrachhari	27.4	3.0	10.8	28.4	2.3	8.0
15	Lakshmipur	26.8	2.3	8.7	28.6	2.1	7.2
16	Noakhali	36.6	2.2	6.1	35.6	2.1	5.8
17	Rangamati	36.0	3.5	9.6	33.8	2.7	8.0
18	Dhaka	33.4	1.7	5.2	33.7	1.7	5.1
19	Faridpur	24.9	2.4	9.8	25.4	2.2	8.5
20	Gazipur	26.5	3.1	11.5	28.4	2.4	8.4
21	Gopalganj	25.1	2.4	9.5	26.1	2.0	7.5
22	Jamalpur	33.2	2.8	8.4	35.2	2.7	7.7
23	Kishoreganj	37.0	2.6	7.0	35.3	2.3	6.5
24	Madaripur	27.3	2.5	9.1	27.6	2.3	8.4
25	Manikganj	19.7	2.7	13.8	24.1	2.4	9.8
26	Munshiganj	25.4	2.4	9.4	26.6	2.0	7.6
27	Mymensingh	33.2	2.2	6.8	33.8	2.0	6.0
28	Narayanganj	24.9	2.5	9.9	26.3	2.3	8.8
29	Narsingdi	28.6	2.4	8.4	28.6	2.0	7.2
30	Netrokona	37.3	2.5	6.7	37.5	2.3	6.1
31	Rajbari	22.1	2.4	11.0	24.2	2.1	8.5
32	Shariatpur	24.7	2.2	9.0	25.4	2.0	7.9
33	Sherpur	27.9	2.6	9.2	30.1	2.5	8.4
34	Tangail	19.6	2.3	11.8	23.5	2.2	9.5
35	Bagerhat	26.8	2.4	9.0	28.3	2.2	7.8
36	Chuadanga	21.3	2.2	10.5	23.3	2.0	8.7
37	Jessore	21.4	2.3	10.8	22.4	1.9	8.6
38	Jhenaidaha	22.6	2.4	10.5	24.1	2.0	8.2
39	Khulna	17.2	2.3	13.5	19.9	2.0	10.1
40	Kushtia	15.3	2.0	13.2	17.6	1.9	10.7
41	Magura	24.3	2.4	10.0	24.0	2.0	8.3
42	Meherpur	15.6	2.0	13.1	18.0	2.0	10.9
43	Narail	27.7	2.5	9.0	26.1	2.0	7.8
44	Satkhira	20.4	2.3	11.2	21.3	2.0	9.5
45	Bogra	31.8	2.7	8.6	32.9	2.4	7.4
46	Joypurhat	29.8	2.7	9.0	31.3	2.5	7.8
47	Naogaon	24.1	2.8	11.6	25.9	2.3	8.7
48	Natore	18.6	2.4	12.7	21.0	2.2	10.4
49	Chapai Nababganj	25.7	2.3	9.0	27.2	2.0	7.4
50	Pabna	26.6	2.4	8.9	28.2	2.3	8.0
51	Rajshahi	24.4	2.4	9.6	25.7	2.2	8.7
52	Sirajganj	27.2	2.3	8.6	27.8	2.1	7.4
53	Dinajpur	24.7	2.5	10.0	26.9	2.4	9.1
54	Gaibandha	29.9	2.5	8.2	30.1	2.3	7.5
55	Kurigram	29.3	2.5	8.5	29.7	2.3	7.8
56	Lalmonirhat	29.7	2.5	8.5	29.6	2.1	7.3
57	Nilphamari	29.5	2.4	8.1	30.6	2.1	6.7
58	Panchagarh	39.8	3.0	7.6	36.7	2.5	6.8
59	Rangpur	16.3	2.1	12.9	19.4	2.0	10.5
60	Thakurgaon	25.9	2.2	8.7	28.1	2.2	7.6
61	Habiganj	34.9	2.3	6.6	36.3	2.1	5.9
62	Maulvi Bazar	30.3	2.6	8.5	32.6	2.3	7.1
63	Sunamganj	44.3	2.3	5.3	43.4	2.2	5.0
64	Sylhet	37.7	2.2	5.8	38.6	2.4	6.1

Table S.6 District level stunting with their standard errors (se) and coefficient of variation (CV) expressed in percentage estimated by the design-based (DIR) and model-based (SAE) estimators.

No.	District	DIR			SAE		
		wasting	se	CV	wasting	se	CV
1	Barguna	13.4	1.7	13.1	12.6	1.5	11.9
2	Barisal	11.0	1.8	16.8	11.2	1.4	12.7
3	Bhola	11.3	1.5	13.2	11.5	1.2	10.5
4	Jhalokati	7.1	1.5	20.4	8.6	1.2	14.5
5	Patuakhali	8.1	1.4	17.5	9.2	1.2	13.4
6	Pirojpur	13.6	1.9	13.8	13.3	1.6	11.9
7	Bandarban	14.6	2.7	18.5	13.0	1.5	11.4
8	Brahmanbaria	7.9	1.3	16.7	8.6	1.0	12.0
9	Chandpur	7.3	1.5	21.1	8.8	1.2	13.6
10	Chittagong	12.6	1.2	9.6	12.1	1.0	8.5
11	Comilla	9.3	1.3	13.5	9.8	1.1	10.8
12	Cox's Bazar	9.7	1.4	14.7	10.2	1.2	11.4
13	Feni	9.0	1.3	14.7	9.0	1.2	13.0
14	Khagrachhari	4.0	1.0	24.8	6.3	1.1	17.9
15	Lakshmipur	15.5	1.8	11.7	13.6	1.6	11.9
16	Noakhali	11.7	1.4	12.3	11.5	1.2	10.6
17	Rangamati	9.4	1.8	18.5	10.1	1.4	13.6
18	Dhaka	9.4	1.1	11.7	9.1	1.0	11.1
19	Faridpur	7.5	1.5	20.0	7.9	1.1	14.0
20	Gazipur	8.9	1.9	21.7	9.4	1.3	13.5
21	Gopalganj	7.2	1.5	21.5	8.1	1.1	13.9
22	Jamalpur	11.0	1.9	17.0	11.9	1.6	13.2
23	Kishoreganj	8.6	1.5	17.6	9.1	1.1	12.5
24	Madaripur	11.1	1.8	15.9	10.5	1.4	13.2
25	Manikganj	4.8	1.2	25.2	6.8	1.1	16.9
26	Munshiganj	8.0	1.4	18.1	8.5	1.1	13.1
27	Mymensingh	6.7	1.1	17.0	8.3	1.1	12.8
28	Narayanganj	6.6	1.3	19.7	7.5	1.1	14.3
29	Narsingdi	6.8	1.4	20.5	8.0	1.1	13.2
30	Netrokona	11.4	1.7	14.7	11.6	1.4	11.8
31	Rajbari	8.0	1.5	19.1	8.3	1.1	13.8
32	Shariatpur	8.3	1.5	17.9	8.4	1.1	13.2
33	Sherpur	15.2	2.0	13.3	14.0	1.7	12.1
34	Tangail	14.1	2.1	14.8	12.4	1.7	14.0
35	Bagerhat	7.7	1.5	18.8	9.0	1.2	13.1
36	Chuadanga	11.8	1.8	15.6	10.8	1.3	12.4
37	Jessore	9.9	1.6	16.5	10.1	1.3	12.6
38	Jhenaidaha	8.7	1.6	18.1	9.5	1.2	12.7
39	Khulna	9.5	1.9	20.2	9.4	1.3	13.6
40	Kushtia	11.2	1.7	15.3	10.7	1.4	12.7
41	Magura	7.4	1.4	19.3	8.3	1.1	13.7
42	Meherpur	7.7	1.5	19.0	8.6	1.2	13.9
43	Narail	8.6	1.6	18.2	9.4	1.2	13.0
44	Satkhira	11.9	1.7	14.2	10.8	1.3	12.3
45	Bogra	12.1	1.9	15.9	11.4	1.5	12.8
46	Joypurhat	12.3	2.1	16.8	11.4	1.6	13.7
47	Naogaon	11.1	2.1	18.9	10.4	1.4	13.8
48	Natore	8.5	1.7	20.0	9.4	1.3	13.7
49	Chapai Nababganj	4.8	1.0	21.2	6.5	1.0	15.8
50	Pabna	9.1	1.5	16.5	9.5	1.2	13.0
51	Rajshahi	8.3	1.6	19.3	8.7	1.2	13.9
52	Sirajganj	11.8	1.8	15.3	11.0	1.3	11.8
53	Dinajpur	11.2	1.8	16.2	11.7	1.4	12.2
54	Gaibandha	7.7	1.4	18.4	9.0	1.2	13.5
55	Kurigram	12.7	1.9	15.1	11.8	1.4	12.0
56	Lalmonirhat	12.5	1.8	14.1	12.0	1.4	11.7
57	Nilphamari	12.3	1.7	13.8	11.4	1.3	11.3
58	Panchagarh	11.6	1.9	16.5	11.4	1.4	12.3
59	Rangpur	10.8	1.7	15.4	10.7	1.4	12.6
60	Thakurgaon	12.0	1.6	13.4	10.9	1.3	11.6
61	Habiganj	12.7	1.6	12.3	12.9	1.4	10.8
62	Maulvi Bazar	15.2	2.3	14.8	14.1	1.6	11.1
63	Sunamganj	7.8	1.2	15.1	9.2	1.1	12.5
64	Sylhet	11.4	1.4	12.3	11.5	1.3	11.5

Table S.7 District level wasting with their standard errors (se) and coefficient of variation (CV) expressed in percentage estimated by the design-based (DIR) and model-based (SAE) estimators.

No.	District	DIR			SAE		
		underweight	se	CV	underweight	se	CV
1	Barguna	23.6	2.2	9.4	25.2	2.2	8.6
2	Barisal	26.3	2.7	10.2	27.1	2.3	8.6
3	Bhola	30.5	2.2	7.2	30.3	1.9	6.4
4	Jhalokati	15.4	2.1	13.4	18.7	1.9	10.1
5	Patuakhali	20.3	2.1	10.1	21.7	2.0	9.1
6	Pirojpur	26.1	2.5	9.7	26.3	2.2	8.4
7	Bandarban	27.8	2.9	10.3	27.7	2.2	7.9
8	Brahmanbaria	19.6	1.8	9.0	21.4	1.7	8.2
9	Chandpur	20.8	2.2	10.8	22.0	2.0	9.1
10	Chittagong	25.1	1.5	6.1	24.9	1.4	5.6
11	Comilla	22.1	1.8	8.0	22.6	1.6	7.0
12	Cox's Bazar	29.4	2.2	7.5	29.1	2.1	7.2
13	Feni	16.0	1.8	11.4	19.1	1.8	9.3
14	Khagrachhari	17.2	2.3	13.5	20.6	2.0	9.9
15	Lakshmipur	25.9	2.3	8.9	25.1	2.0	7.8
16	Noakhali	24.0	2.0	8.5	24.7	1.8	7.1
17	Rangamati	17.9	2.4	13.6	21.1	2.2	10.5
18	Dhaka	18.8	1.4	7.5	18.9	1.3	7.1
19	Faridpur	15.9	2.1	12.9	17.5	1.8	10.5
20	Gazipur	15.7	2.4	15.4	18.8	2.0	10.6
21	Gopalganj	17.9	2.2	12.2	18.9	1.8	9.6
22	Jamalpur	29.1	3.0	10.4	29.4	2.5	8.5
23	Kishoreganj	27.3	2.3	8.3	26.5	2.0	7.4
24	Madaripur	24.8	2.4	9.9	24.8	2.2	8.7
25	Manikganj	12.5	2.0	16.3	16.2	2.0	12.1
26	Munshiganj	14.2	1.8	12.6	16.6	1.7	10.2
27	Mymensingh	21.2	1.9	8.9	23.0	1.8	7.8
28	Narayanganj	13.6	1.8	13.6	16.1	1.8	11.4
29	Narsingdi	21.6	2.2	10.4	21.4	1.8	8.6
30	Netrokona	30.6	2.3	7.6	30.1	2.1	7.1
31	Rajbari	22.3	2.5	11.1	22.6	1.9	8.6
32	Shariatpur	24.5	2.2	9.2	24.0	2.0	8.2
33	Sherpur	27.4	2.6	9.4	28.7	2.5	8.8
34	Tangail	22.8	2.4	10.6	22.6	2.2	9.6
35	Bagerhat	21.2	2.3	10.7	21.9	2.0	9.0
36	Chuadanga	22.4	2.3	10.3	23.4	2.1	8.8
37	Jessore	17.5	2.2	12.3	19.2	1.9	9.8
38	Jhenaidaha	18.2	2.3	12.6	19.8	1.9	9.4
39	Khulna	18.8	2.3	12.1	19.4	1.9	9.9
40	Kushtia	20.2	2.2	10.7	20.9	1.9	9.2
41	Magura	15.1	1.9	12.5	17.1	1.7	10.1
42	Meherpur	18.5	2.2	11.8	18.8	2.0	10.4
43	Narail	22.6	2.1	9.3	21.8	1.9	8.8
44	Satkhira	19.0	2.2	11.5	20.4	1.9	9.5
45	Bogra	26.3	2.5	9.5	26.2	2.2	8.4
46	Joypurhat	25.4	2.8	10.9	26.2	2.3	8.9
47	Naogaon	22.6	2.8	12.3	22.8	2.2	9.7
48	Natore	18.6	2.5	13.2	19.9	2.1	10.3
49	Chapai Nababganj	22.3	2.2	9.7	23.5	1.9	8.1
50	Pabna	18.8	2.0	10.6	21.2	2.0	9.3
51	Rajshahi	24.1	2.5	10.5	24.7	2.2	9.0
52	Sirajganj	28.3	2.5	8.9	28.2	2.2	7.7
53	Dinajpur	19.7	2.2	11.0	22.1	2.2	9.9
54	Gaibandha	29.0	2.3	7.8	27.8	2.2	7.9
55	Kurigram	18.5	2.1	11.4	21.3	2.0	9.3
56	Lalmonirhat	24.4	2.2	8.9	24.0	1.9	7.9
57	Nilphamari	25.7	2.4	9.4	26.0	2.0	7.6
58	Panchagarh	31.0	2.7	8.7	29.3	2.1	7.3
59	Rangpur	18.7	2.1	11.3	20.9	2.0	9.6
60	Thakurgaon	19.1	1.8	9.6	20.5	1.9	9.2
61	Habiganj	39.1	2.3	5.8	38.7	2.2	5.6
62	Maulvi Bazar	28.6	2.3	8.0	30.2	2.2	7.1
63	Sunamganj	29.8	2.0	6.9	31.0	1.9	6.1
64	Sylhet	32.8	2.2	6.8	33.4	2.2	6.5

Table S.8 District level underweight with their standard errors (se) and coefficient of variation (CV) expressed in percentage estimated by the design-based (DIR) and model-based (SAE) estimators.

S.6 District level prevalence of child undernutrition by place of residence

District level estimates of stunting, wasting and underweight by place of residence are plotted in Figures S.3 to S.9 by their respective divisions. Model-based estimates with their approximate 95% confidence intervals (orange for rural and sky-blue for urban) are plotted along with the corresponding design-based point estimates (black empty circle).

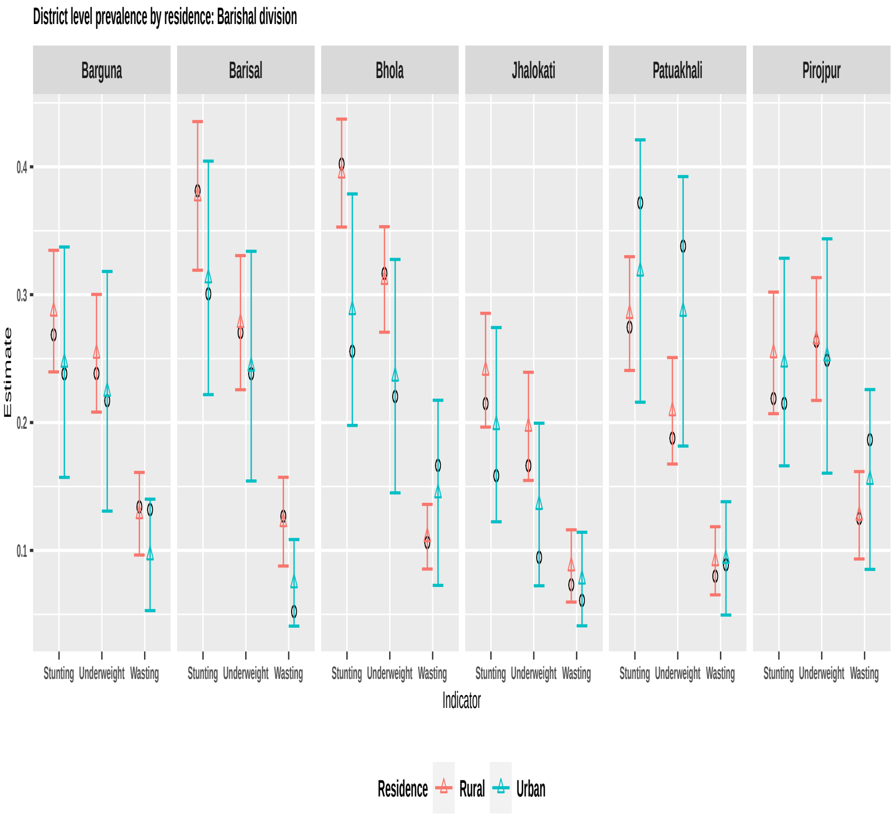

Fig. S.3 District level prevalence of stunting, wasting and underweight by place of residence estimated by design-based (circle dot) and model-based (triangle dot) estimators (with 95% CI) for the districts under Barishal division.

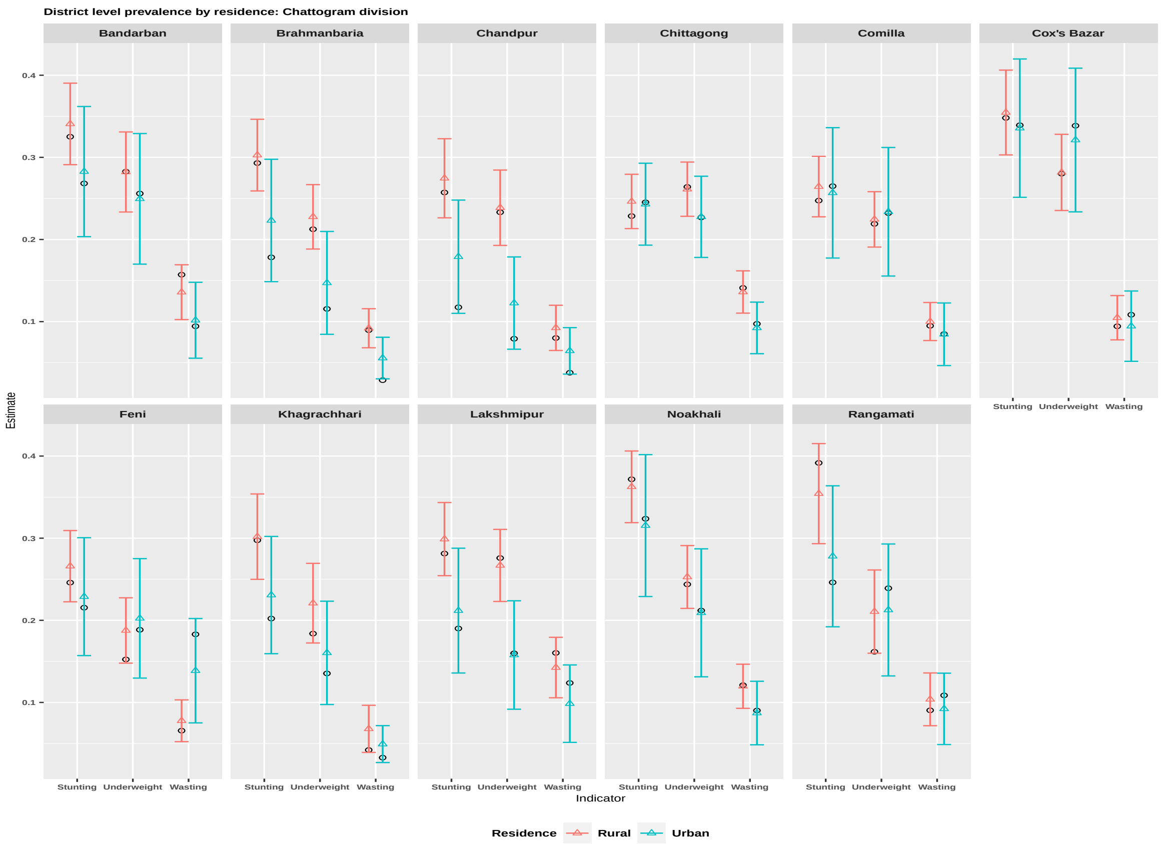

Fig. S.4 District level prevalence of stunting, wasting and underweight by place of residence estimated by design-based (circle dot) and model-based (triangle dot) estimators (with 95% CI) for the districts under Chattogram division.

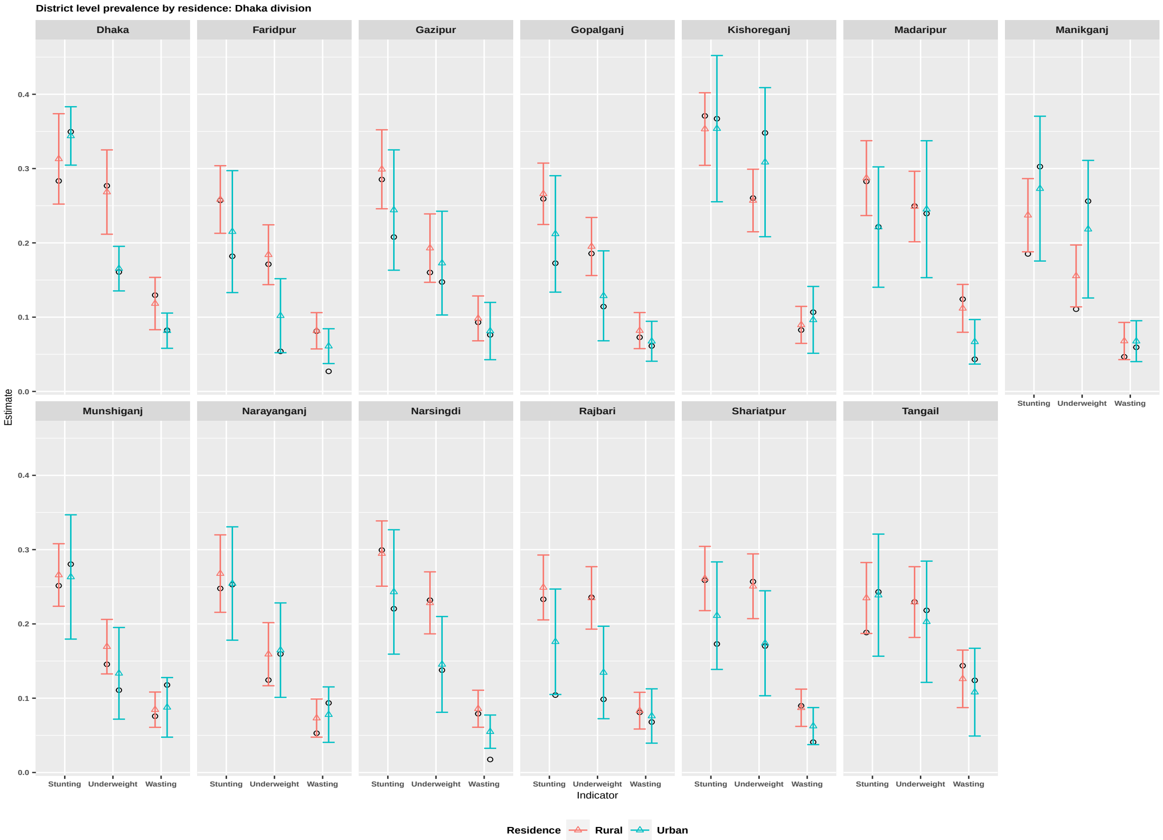

Fig. S.5 District level prevalence of stunting, wasting and underweight by place of residence estimated by design-based (circle dot) and model-based (triangle dot) estimators (with 95% CI) for the districts under Dhaka division.

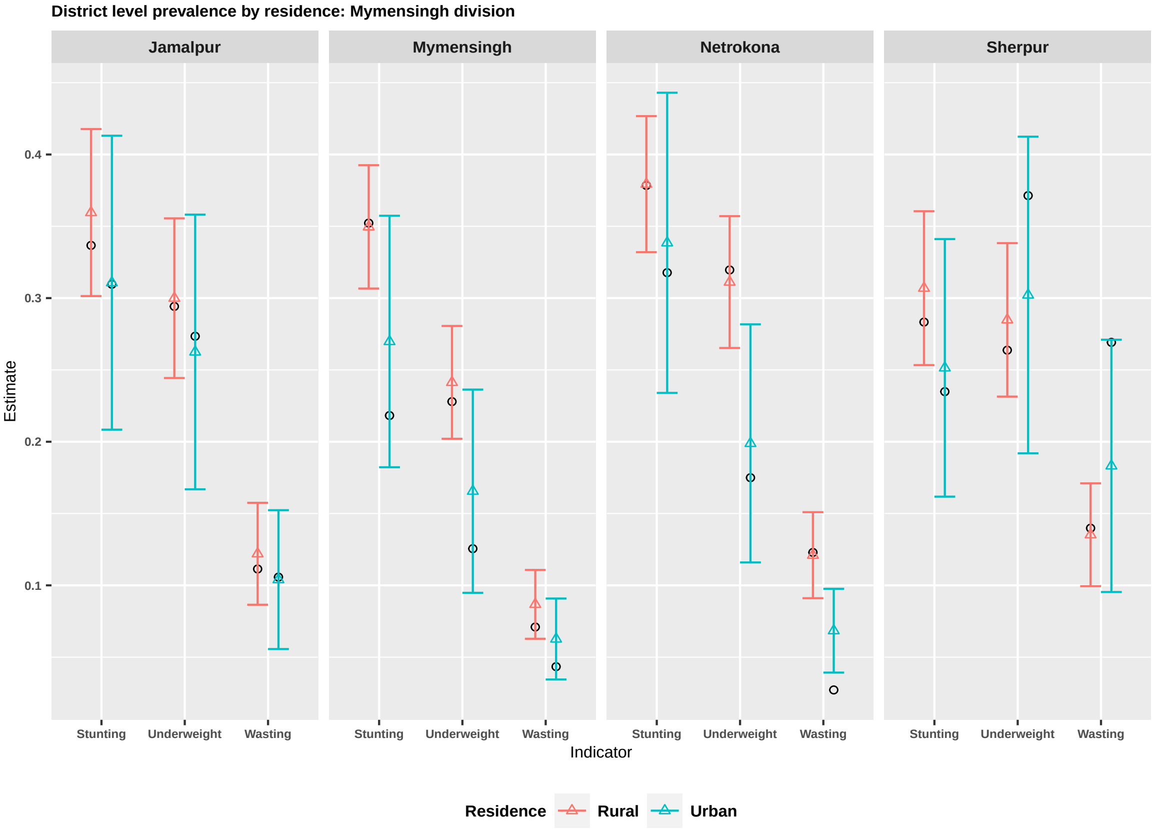

Fig. S.6 District level prevalence of stunting, wasting and underweight by place of residence estimated by design-based (circle dot) and model-based (triangle dot) estimators (with 95% CI) for the districts under Mymensingh division.

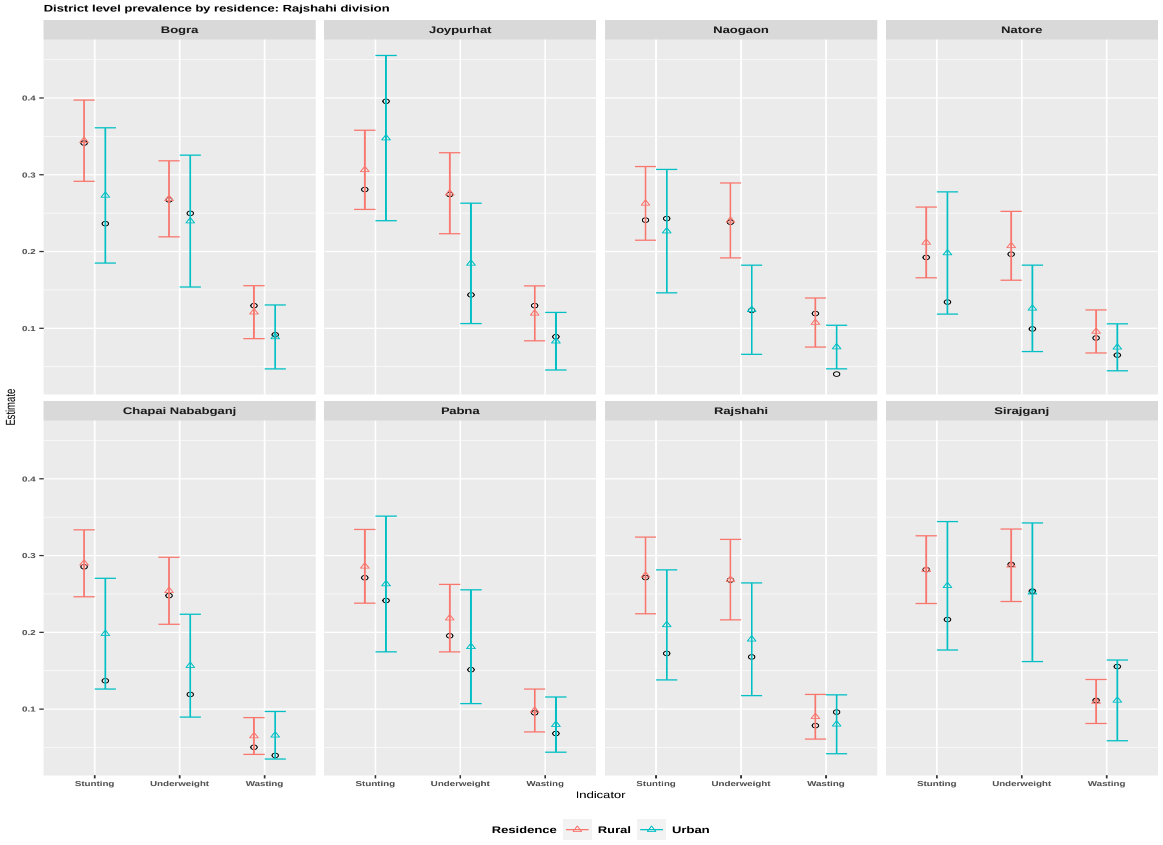

Fig. S.7 District level prevalence of stunting, wasting and underweight by place of residence estimated by design-based (circle dot) and model-based (triangle dot) estimators (with 95% CI) for the districts under Rajshahi division.

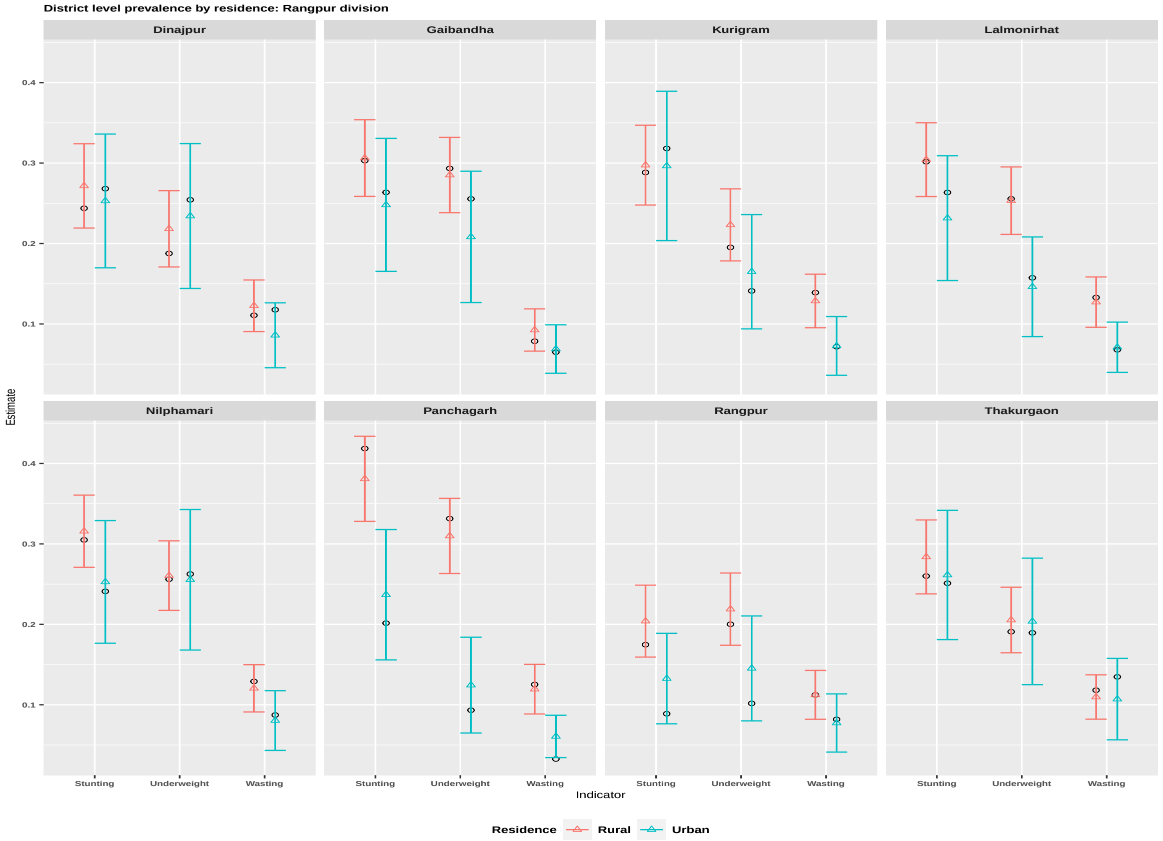

Fig. S.8 District level prevalence of stunting, wasting and underweight by place of residence estimated by design-based (circle dot) and model-based (triangle dot) estimators (with 95% CI) for the districts under Rangpur division.

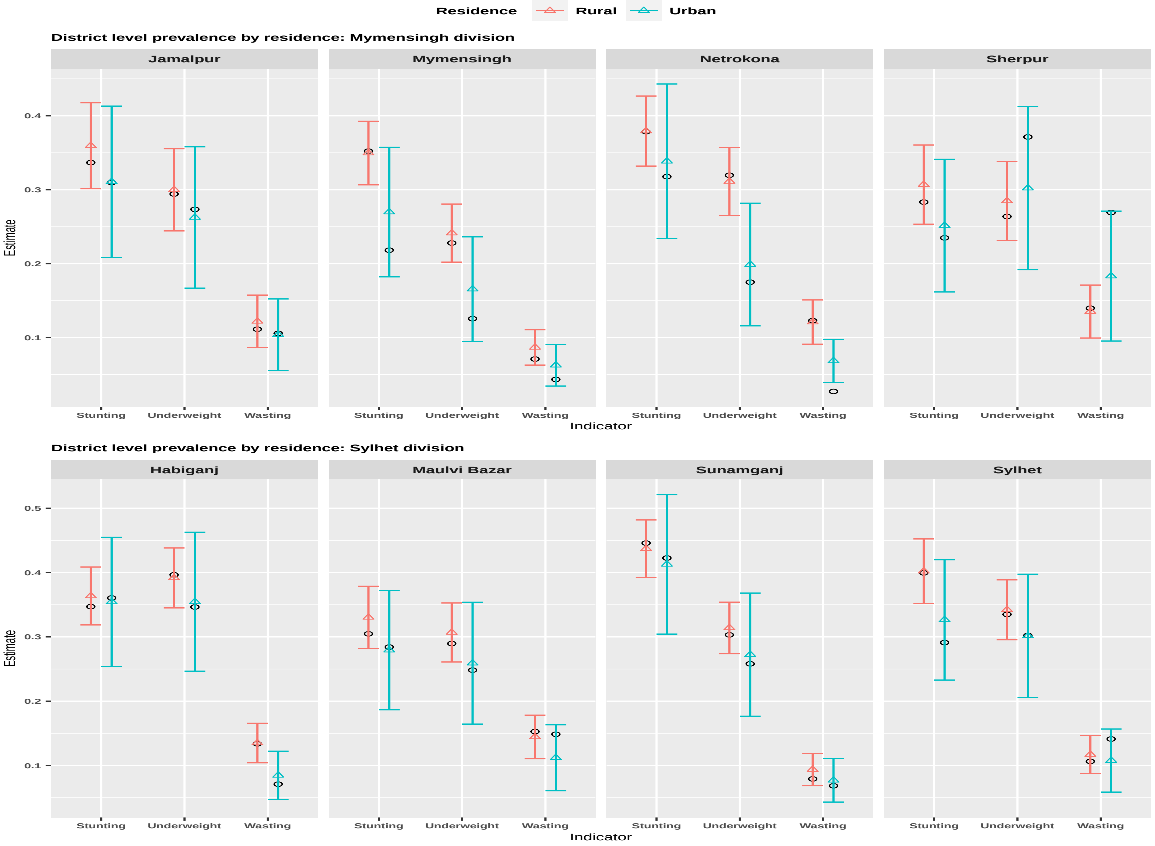

Fig. S.9 District level prevalence of stunting, wasting and underweight by place of residence estimated by design-based (circle dot) and model-based (triangle dot) estimators (with 95% CI) for the districts under Sylhet division.

Springer Nature 2021 LaTeX template

S.7 District level prevalence of child undernutrition by Age-groups

District level stunting, wasting and underweight by children age-groups estimated by model-based estimator are mapped in Figure S.10. Model-based estimates along with design-based direct estimates of district level prevalence of stunting, wasting and underweight by children age-groups are plotted in Figures S.11 to S.17 by their respective divisions.

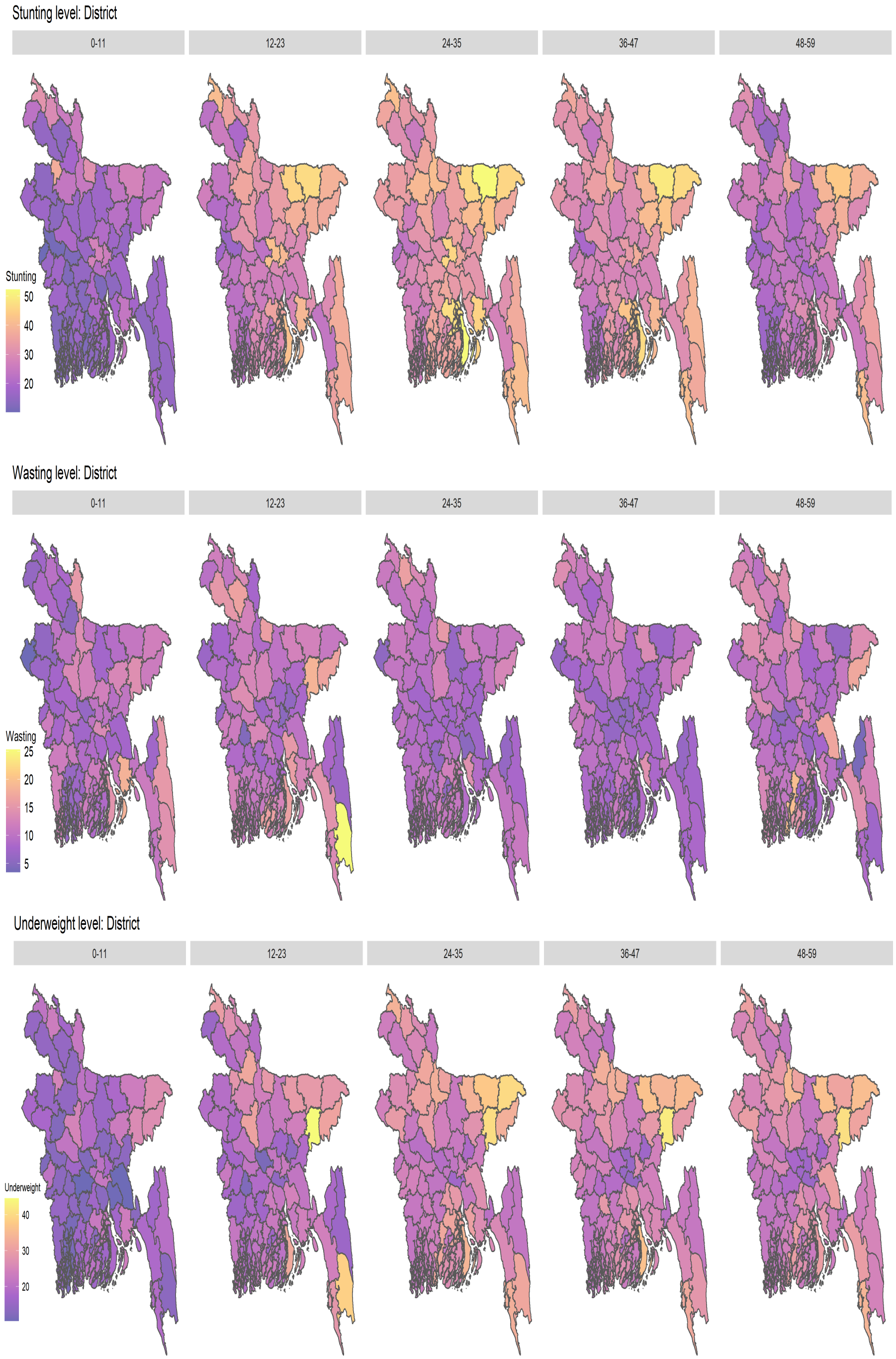

Fig. S.10 District level map for the prevalence of stunting, wasting and underweight by children age-groups.

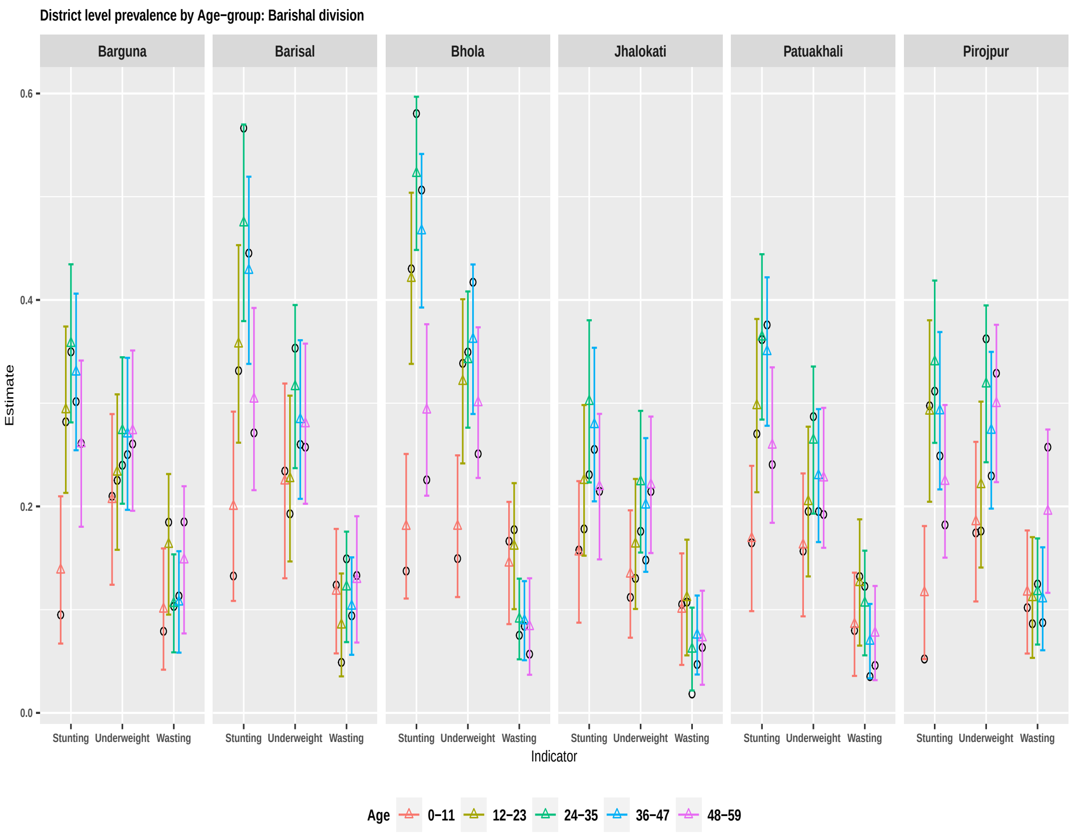

Fig. S.11 District level prevalence of stunting, wasting and underweight by children age-groups estimated by design-based (circle dot) and model-based (triangle dot) estimators (with 95% CI) for the districts under Barishal division.

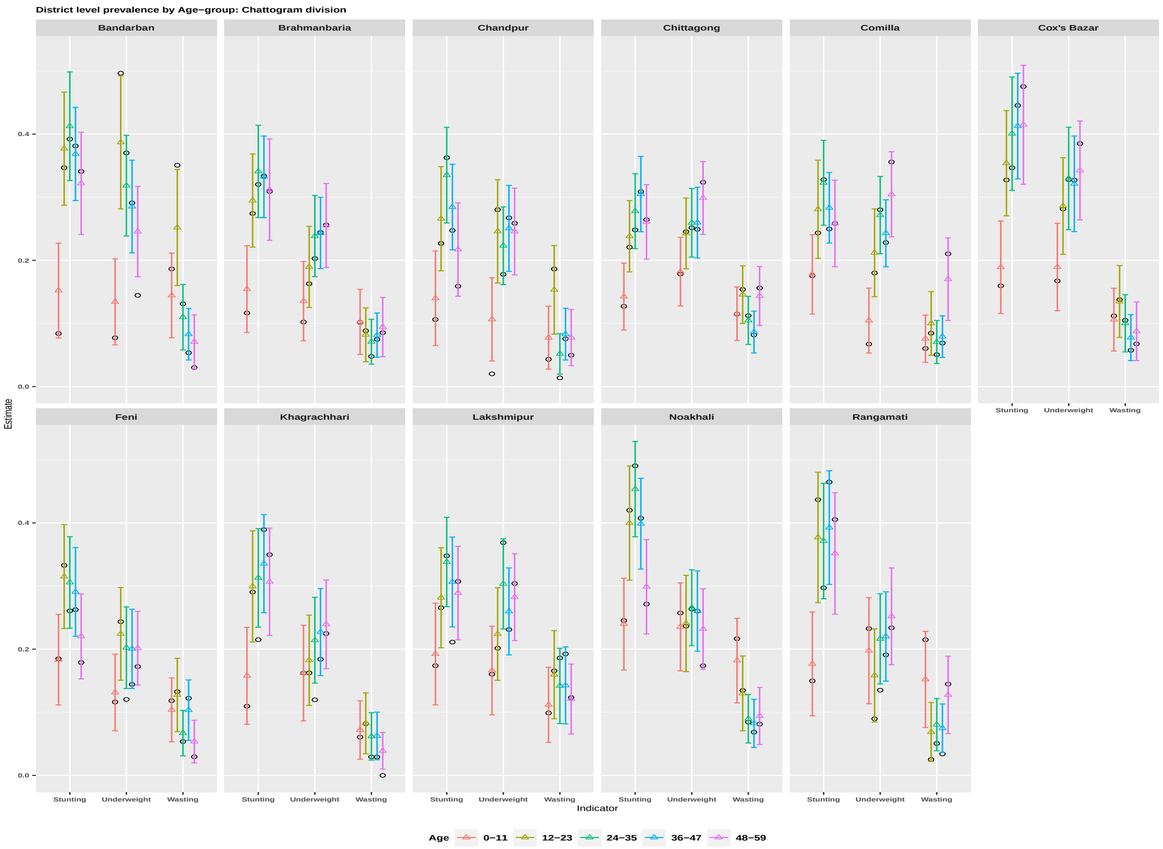

Fig. S.12 District level prevalence of stunting, wasting and underweight by children age-groups estimated by design-based (circle dot) and model-based (triangle dot) estimators (with 95% CI) for the districts under Chattogram division.

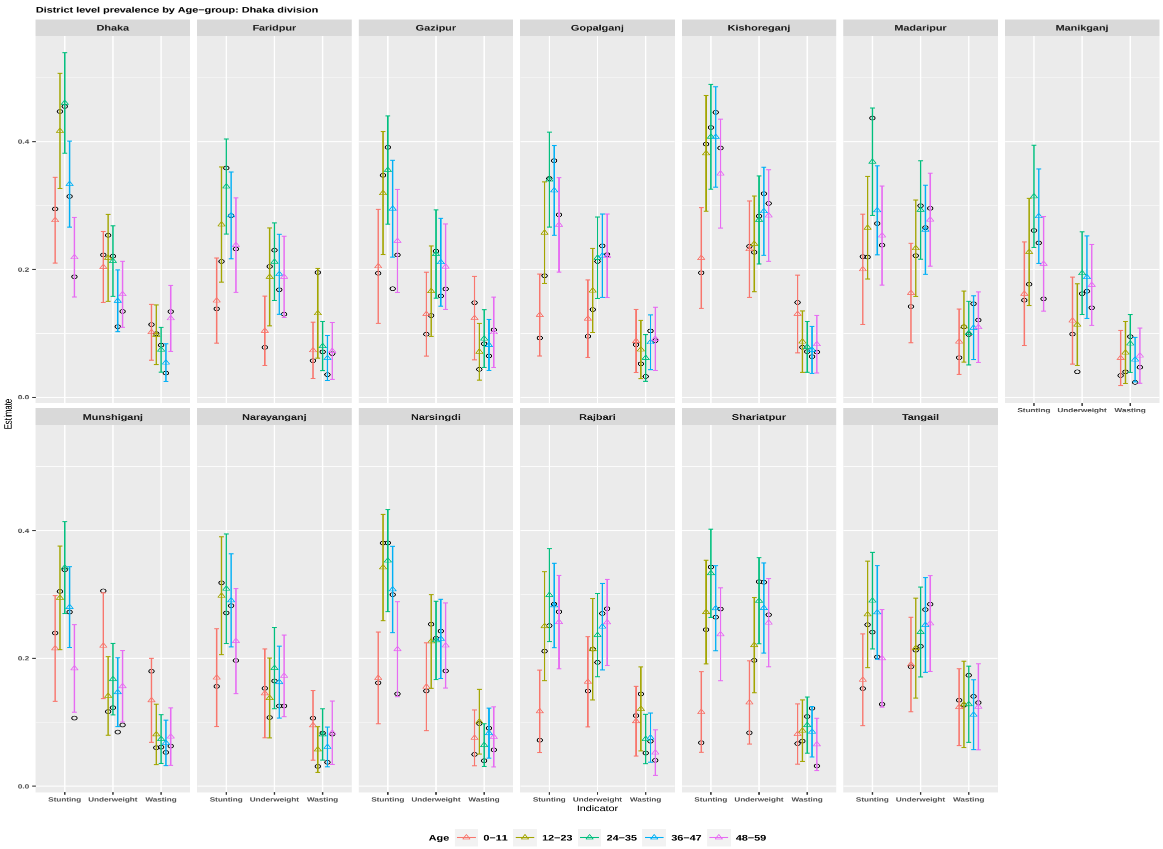

Fig. S.13 District level prevalence of stunting, wasting and underweight by children age-groups estimated by design-based (circle dot) and model-based (triangle dot) estimators (with 95% CI) for the districts under Dhaka division.

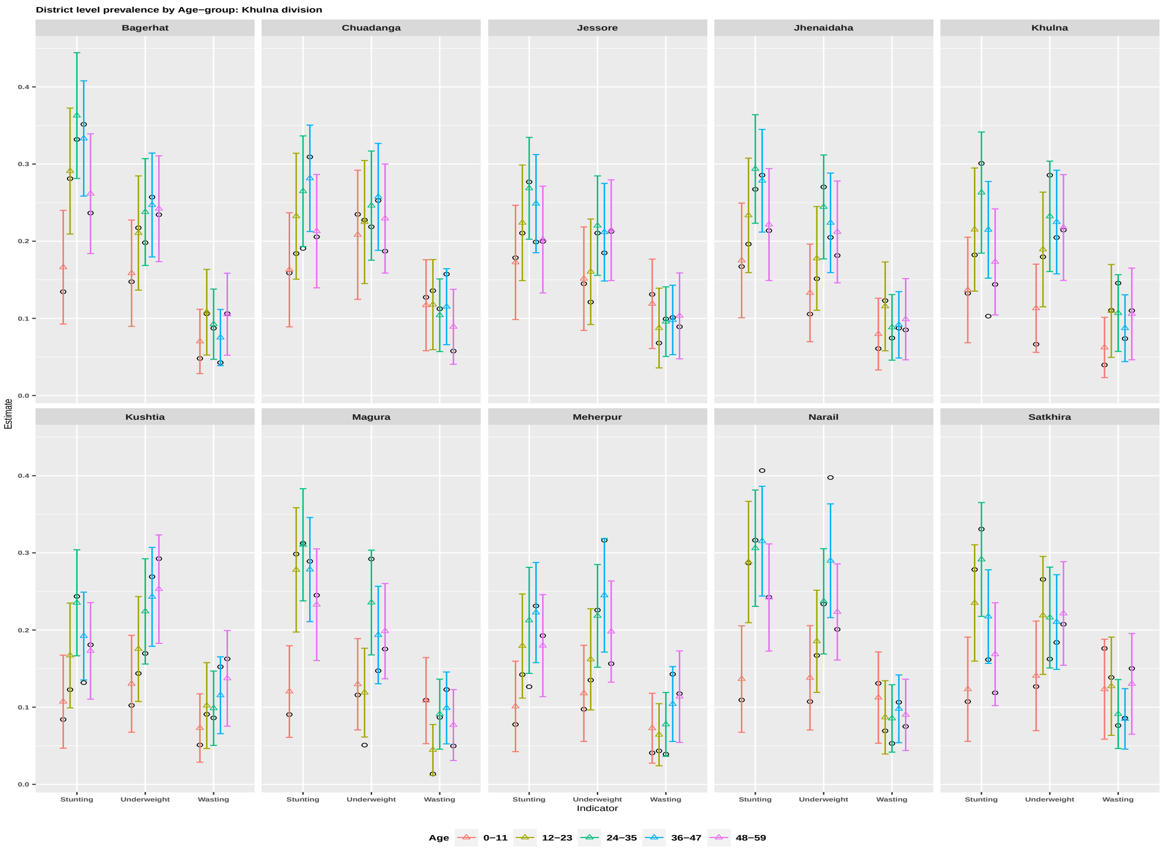

Fig. S.14 District level prevalence of stunting, wasting and underweight by children age-groups estimated by design-based (circle dot) and model-based (triangle dot) estimators (with 95% CI) for the districts under Khulna division.

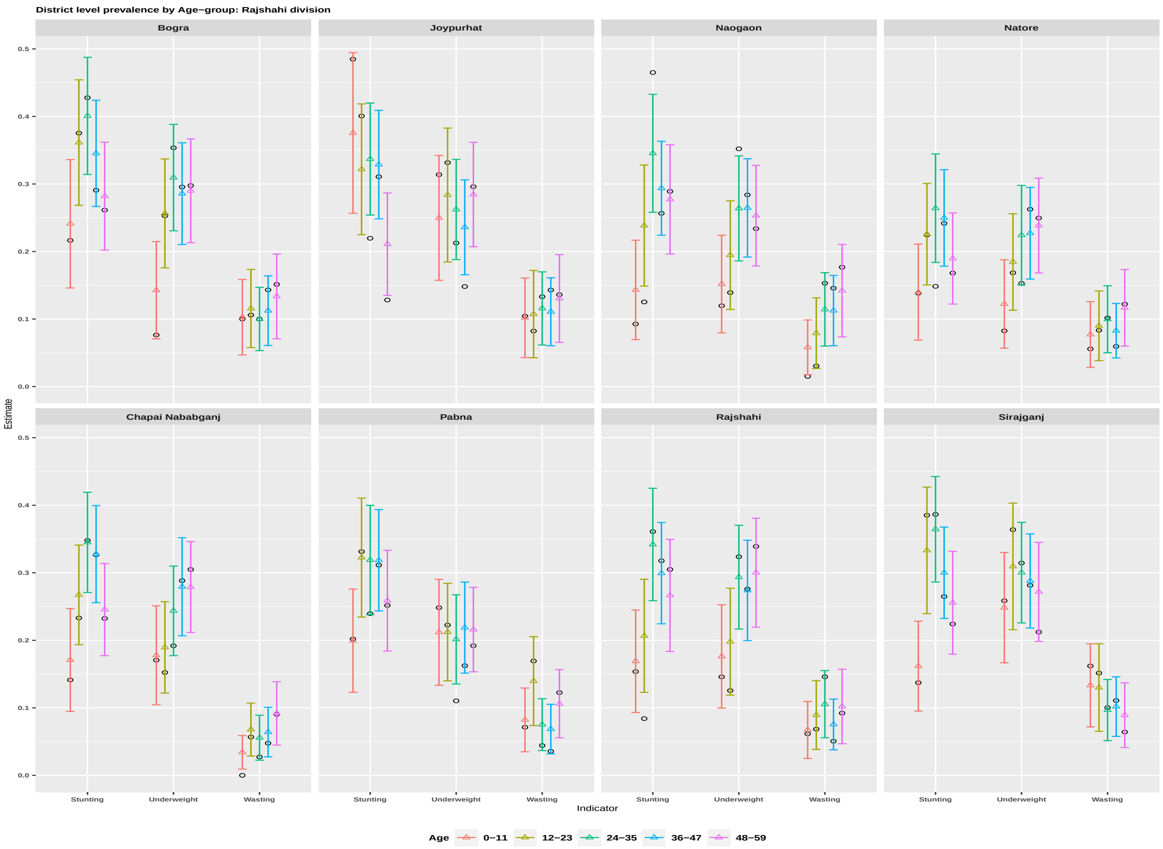

Fig. S.15 District level prevalence of stunting, wasting and underweight by children age-groups estimated by design-based (circle dot) and model-based (triangle dot) estimators (with 95% CI) for the districts under Rajshahi division.

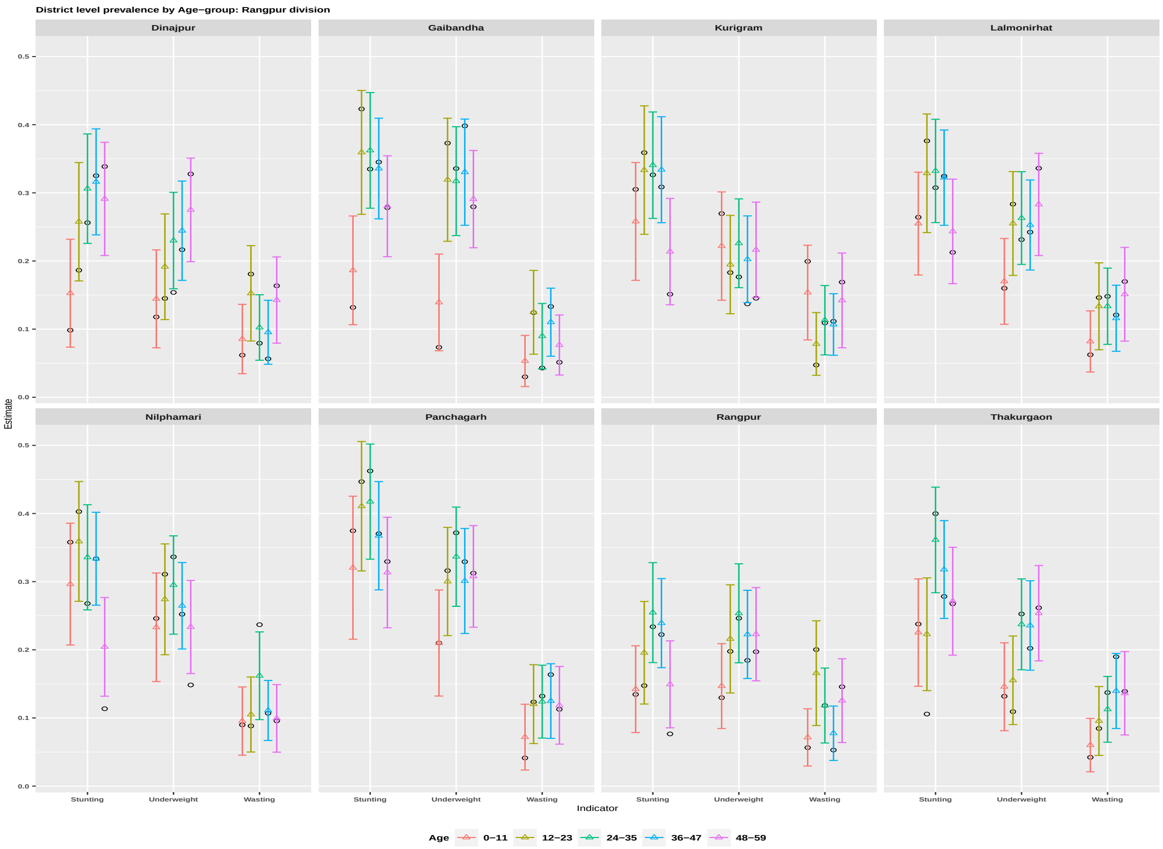

Fig. S.16 District level prevalence of stunting, wasting and underweight by children age-groups estimated by design-based (circle dot) and model-based (triangle dot) estimators (with 95% CI) for the districts under Rangpur division.

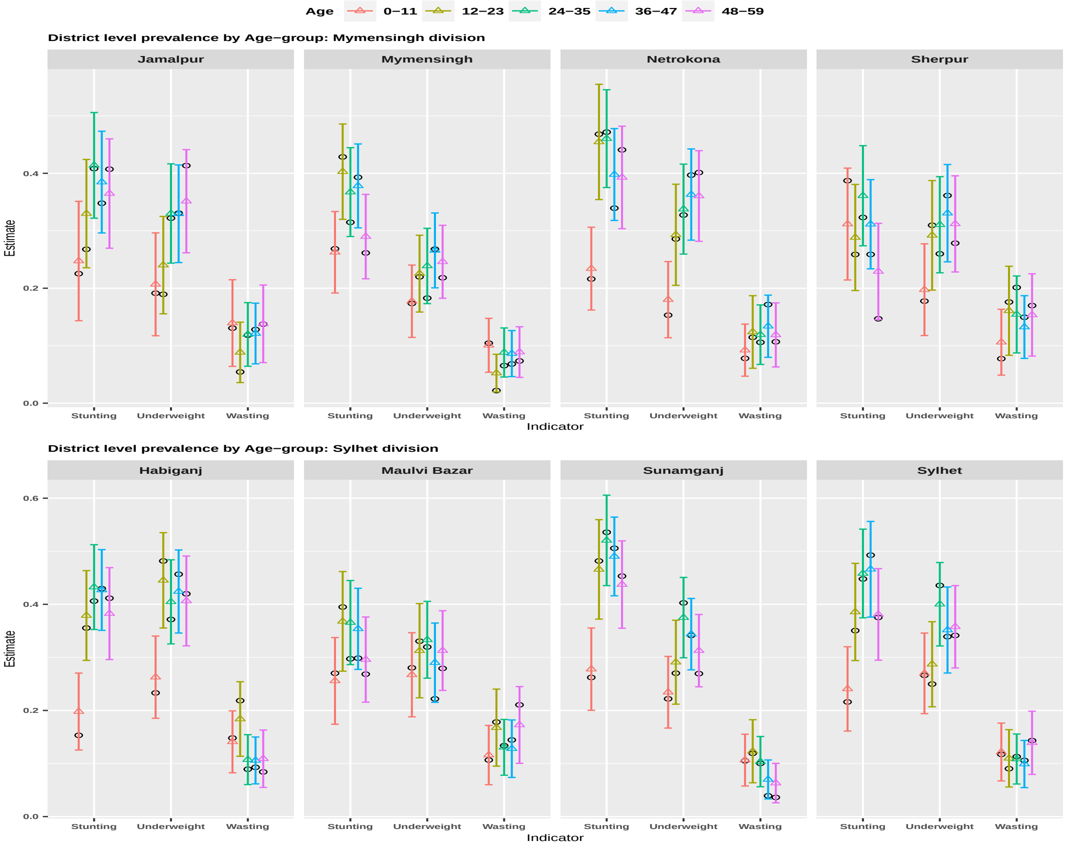

Fig. S.17 District level prevalence of stunting, wasting and underweight by children age-groups estimated by design-based (circle dot) and model-based (triangle dot) estimators (with 95% CI) for the districts under Mymensingh and Sylhet divisions.

S.8 District level prevalence of child undernutrition by age-groups and place of residence

District-age-residence level stunting, wasting and underweight estimated by model-based and design-based estimators are plotted in Figures S.18 to S.25 by their respective divisions.

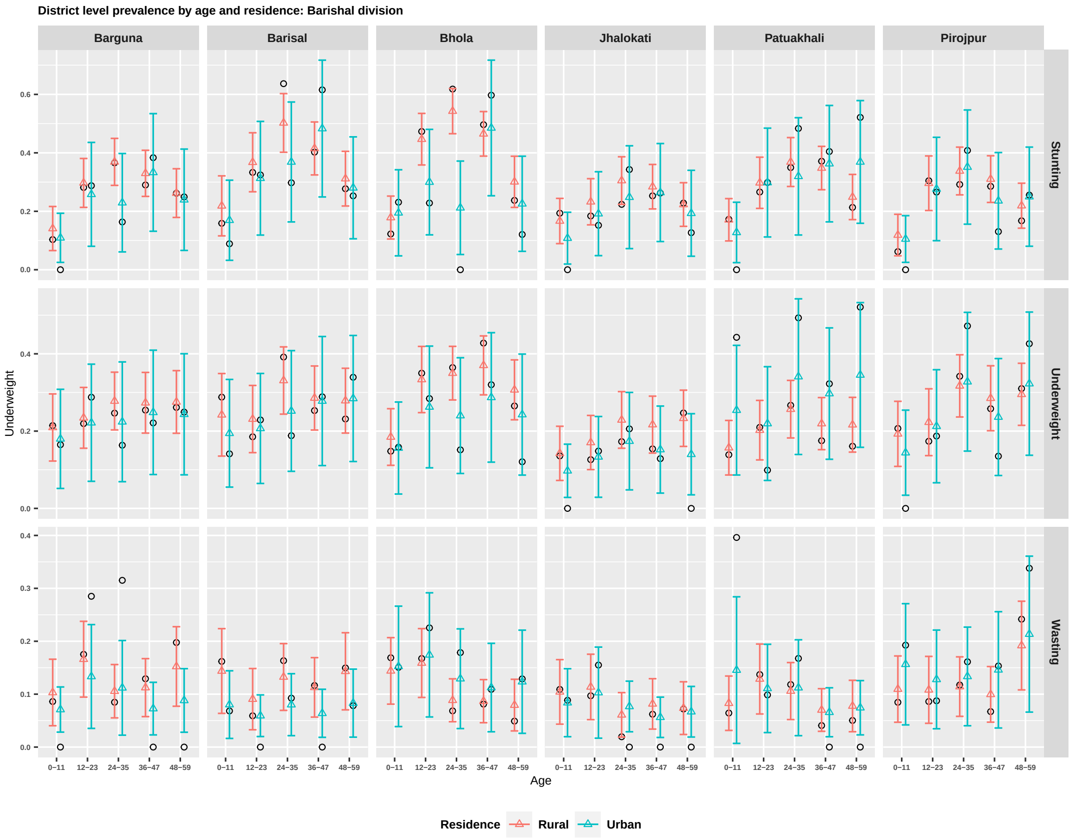

Fig. S.18 District level prevalence of stunting, wasting and underweight by children age-groups and place of residence estimated by design-based (circle dot) and model-based (triangle dot) estimators (with 95% CI) for the districts under Barishal division.

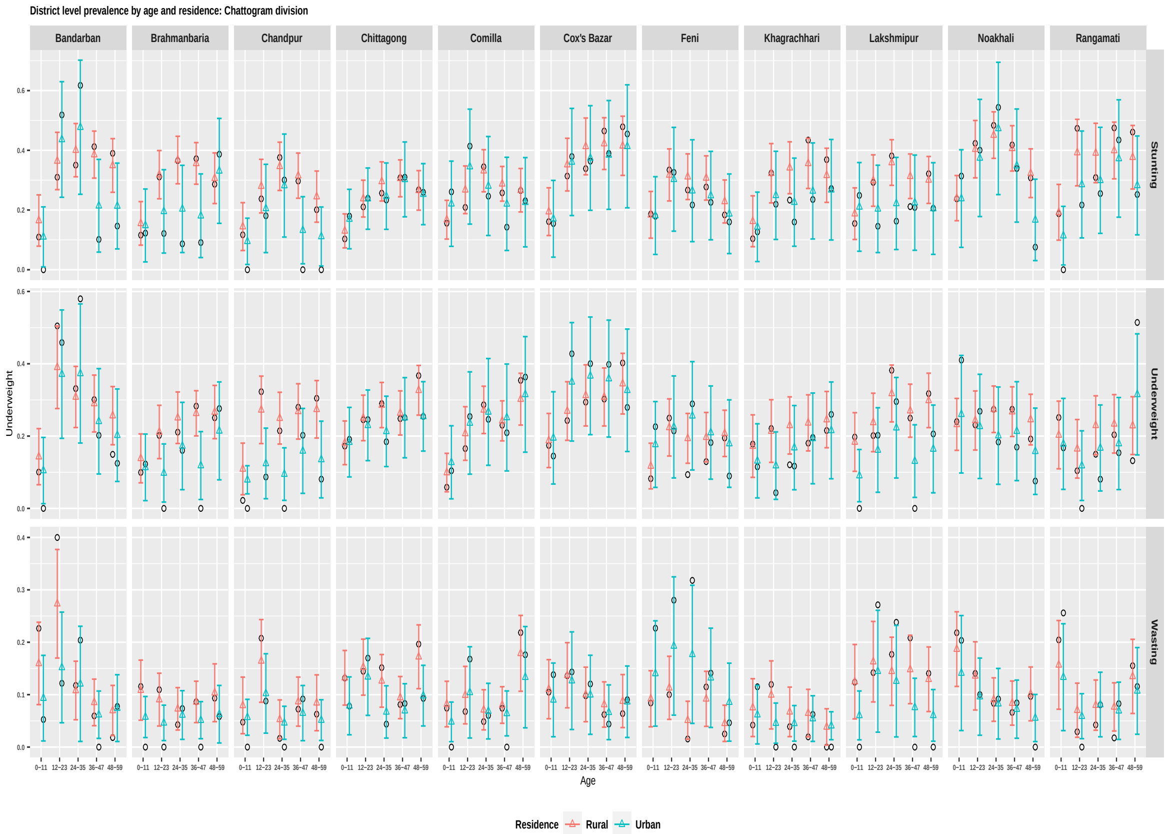

Fig. S.19 District level prevalence of stunting, wasting and underweight by children age-groups and place of residence estimated by design-based (circle dot) and model-based (triangle dot) estimators (with 95% CI) for the districts under Chattogram division.

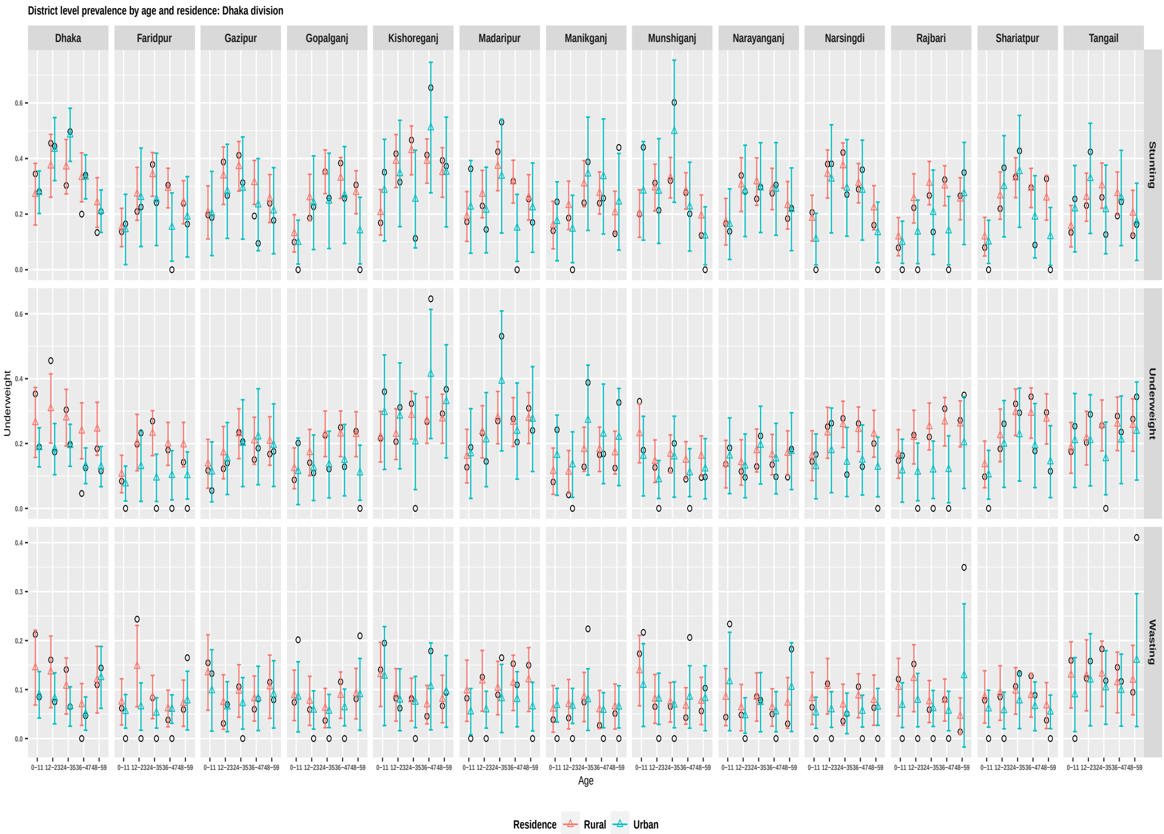

Fig. S.20 District level prevalence of stunting, wasting and underweight by children age-groups and place of residence estimated by design-based (circle dot) and model-based (triangle dot) estimators (with 95% CI) for the districts under Dhaka division.

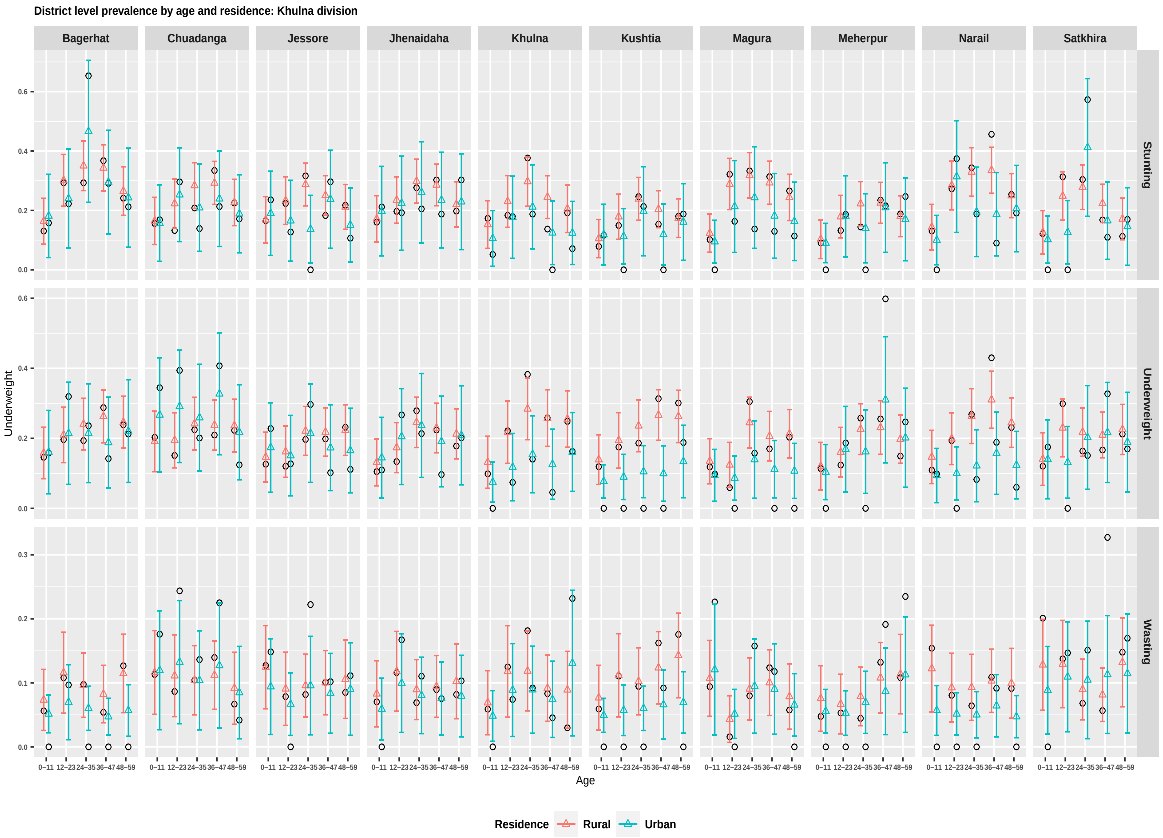

Fig. S.21 District level prevalence of stunting, wasting and underweight by children age-groups estimated by design-based (circle dot) and model-based (triangle dot) estimators (with 95% CI) for the districts under Khulna division.

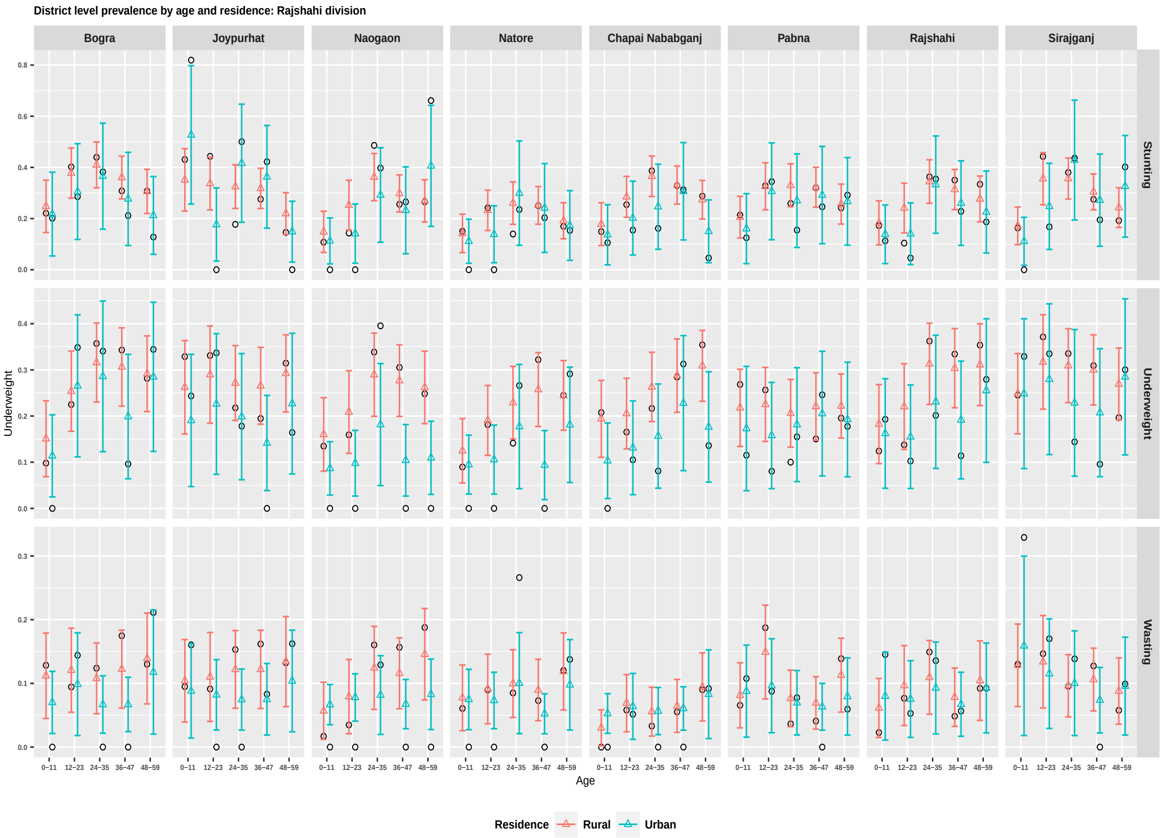

Fig. S.22 District level prevalence of stunting, wasting and underweight by children age-groups and place of residence estimated by design-based (circle dot) and model-based (triangle dot) estimators (with 95% CI) for the districts under Rajshahi division.

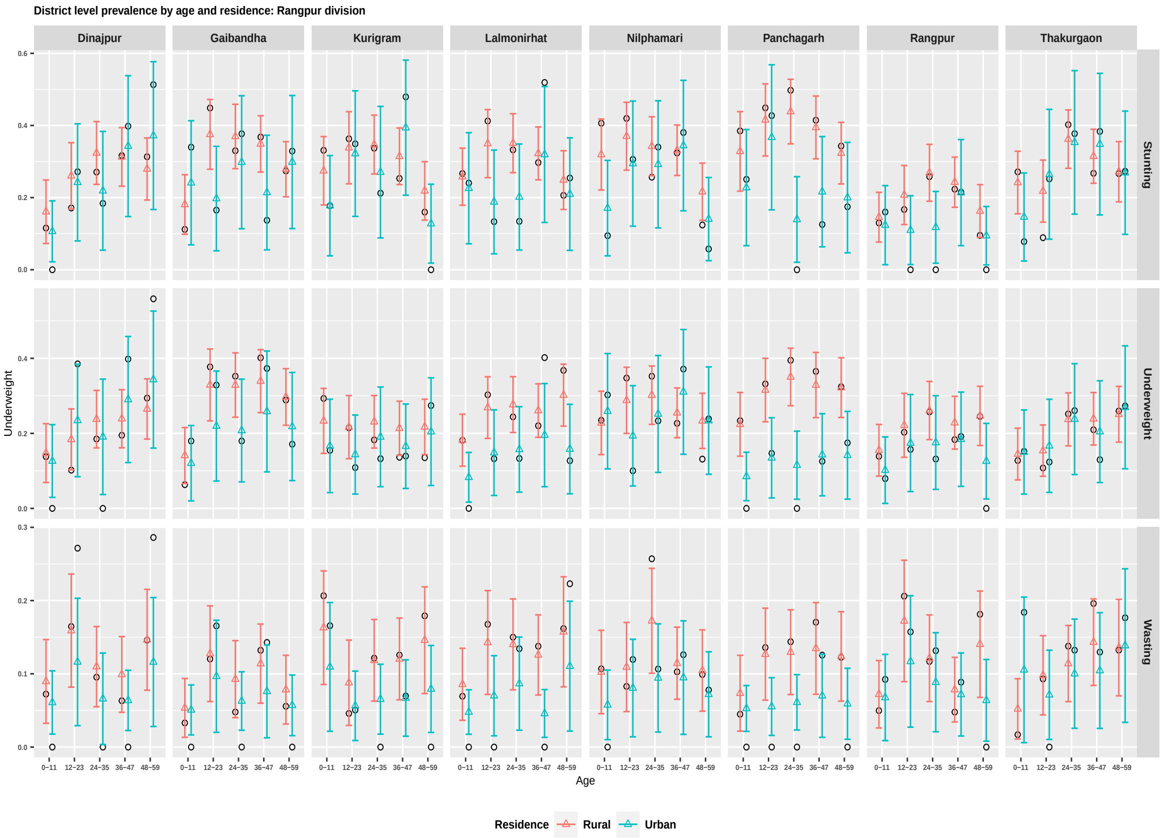

Fig. S.23 District level prevalence of stunting, wasting and underweight by children age-groups and place of residence estimated by design-based (circle dot) and model-based (triangle dot) estimators (with 95% CI) for the districts under Rangpur division.

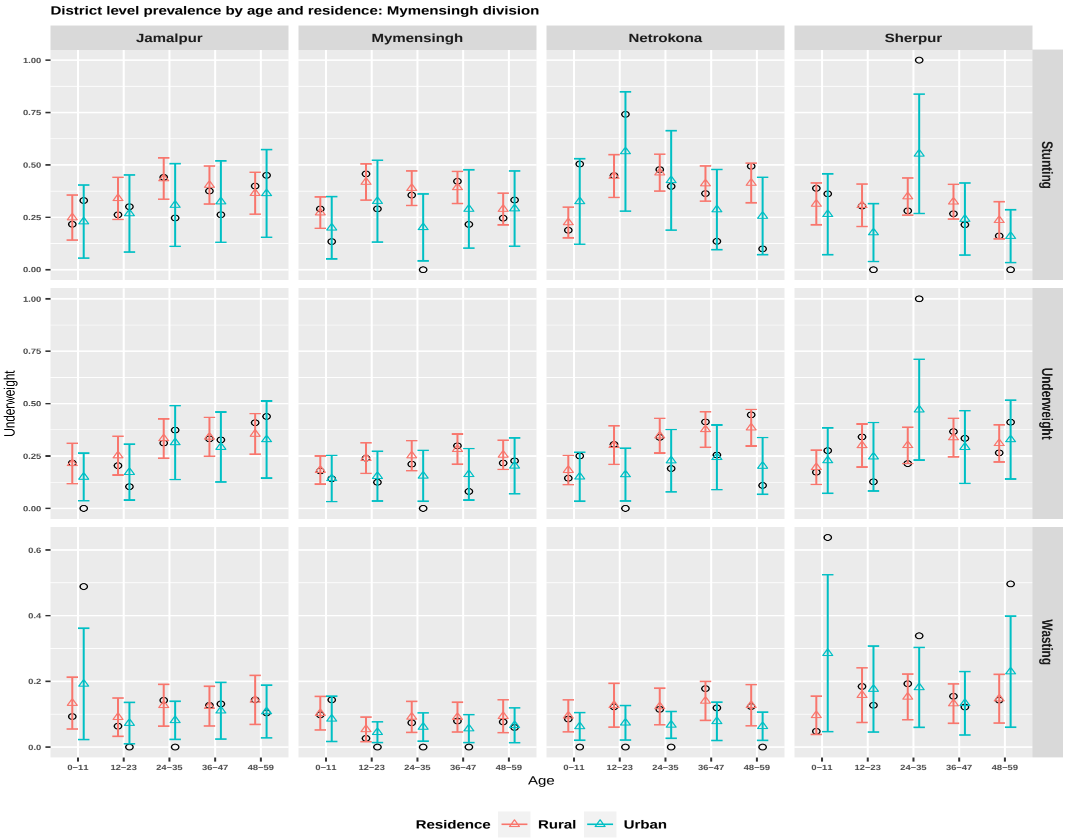

Fig. S.24 District level prevalence of stunting, wasting and underweight by children age-groups and place of residence estimated by design-based (circle dot) and model-based (triangle dot) estimators (with 95% CI) for the districts under Mymensingh division.

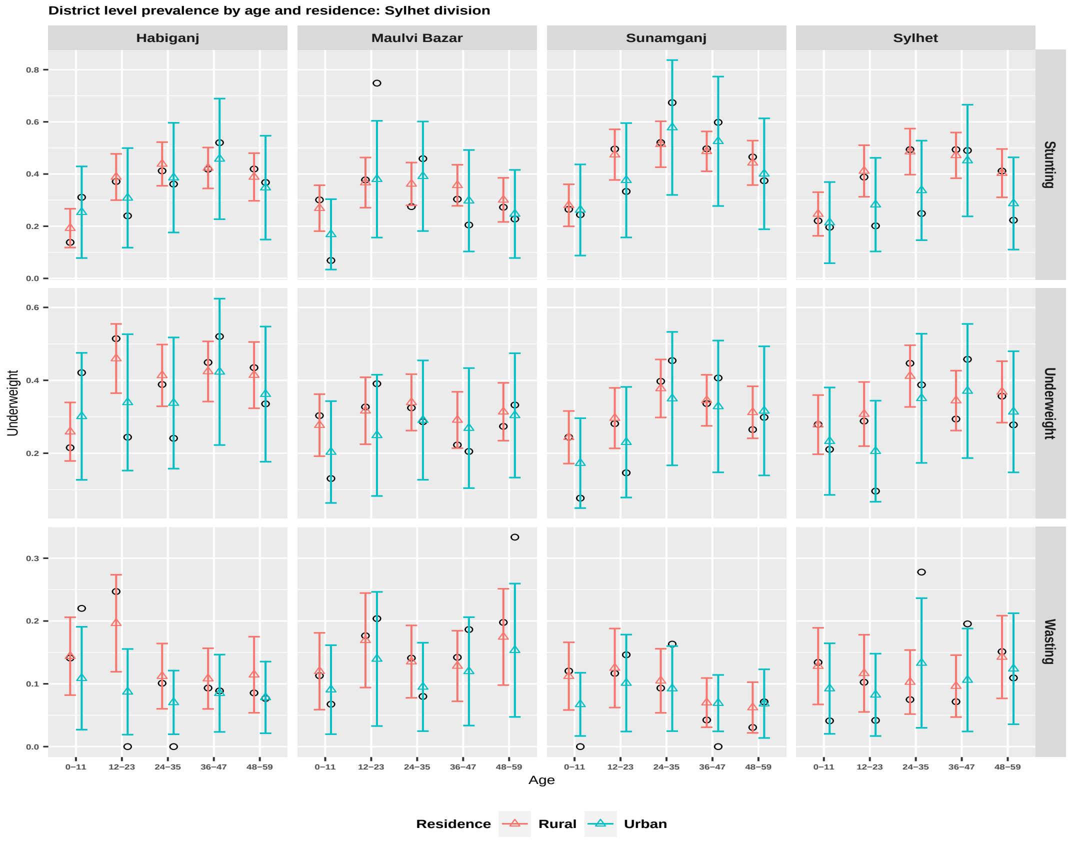

Fig. S.25 District level prevalence of stunting, wasting and underweight by children age-groups and place of residence estimated by design-based (circle dot) and model-based (triangle dot) estimators (with 95% CI) for the districts under Sylhet division.

Springer Nature 2021 LaTeX template

Springer Nature 2021 LaTeX template

S.9 Detailed level prevalence of Stunting

The detailed district-residence-age-sex level stunting estimated by SAE and DIR estimators are plotted in Figures S.26 to S.33 by their respective divisions.

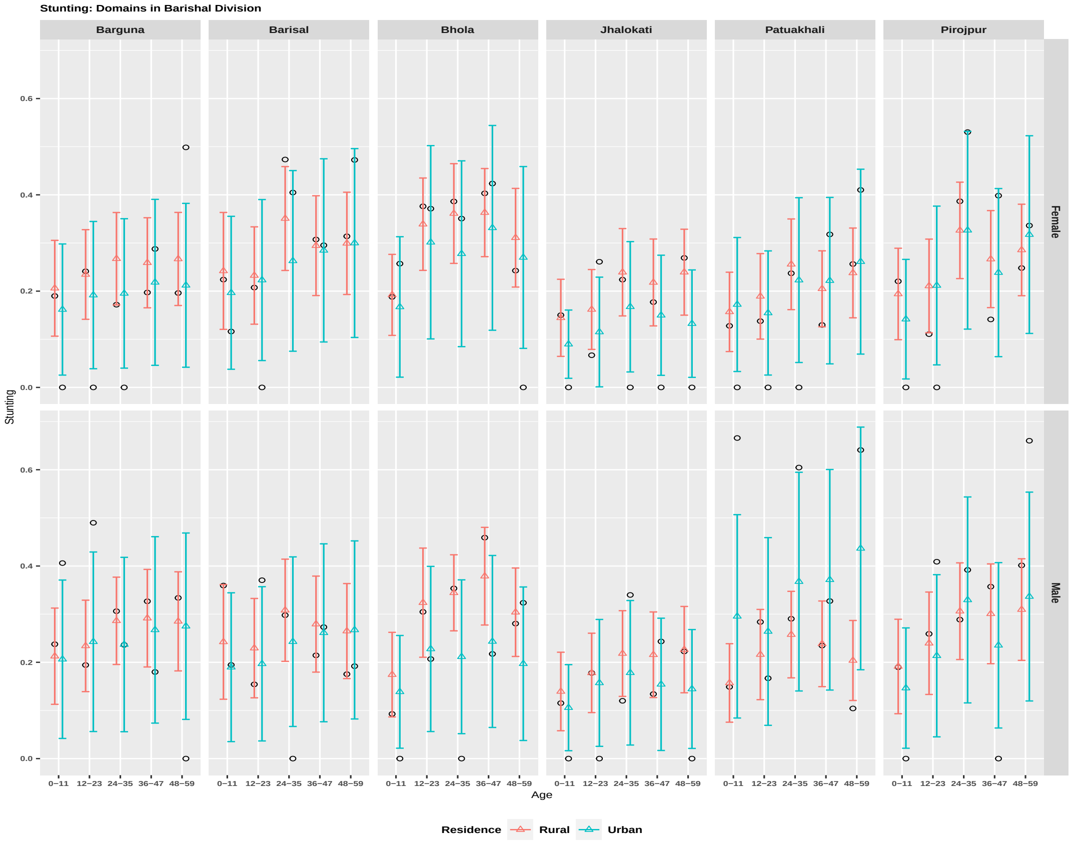

Fig. S.26 Prevalence of stunting for the cross-classified domains of district, residence, children age-group and sex belong to Barishal division estimated by design-based (circle dot) and model-based (triangle dot) estimators (with 95% CI) .

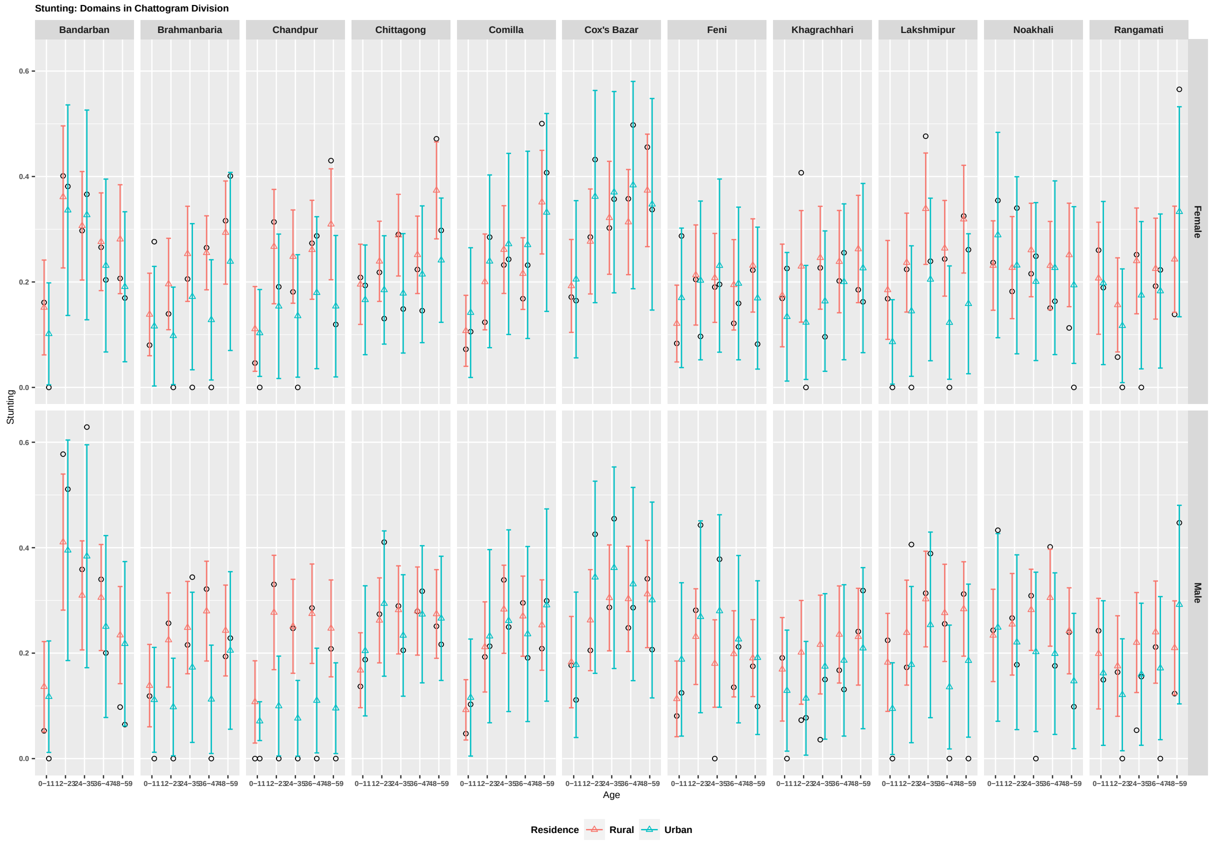

Fig. S.27 Prevalence of stunting for the cross-classified domains of district, residence, children age-group and sex belong to Chattogram division estimated by design-based (circle dot) and model-based (triangle dot) estimators (with 95% CI) .

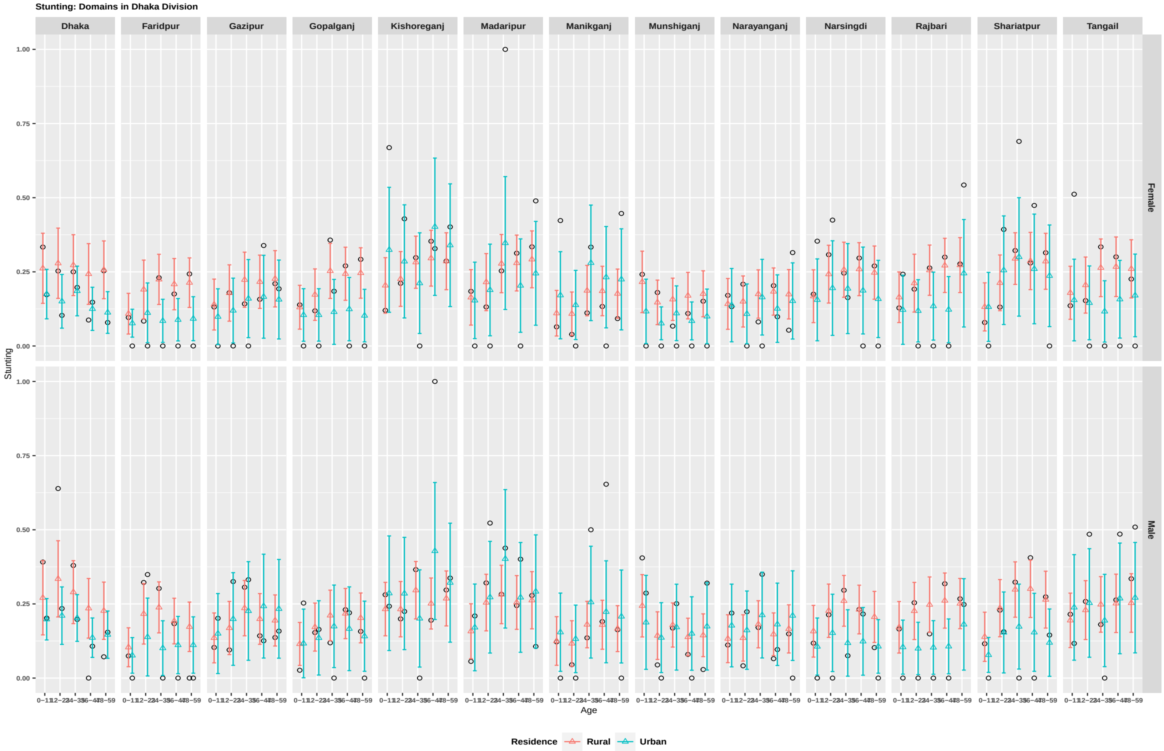

Fig. S.28 Prevalence of stunting for the cross-classified domains of district, residence, children age-group and sex belong to Dhaka division estimated by design-based (circle dot) and model-based (triangle dot) estimators (with 95% CI) .

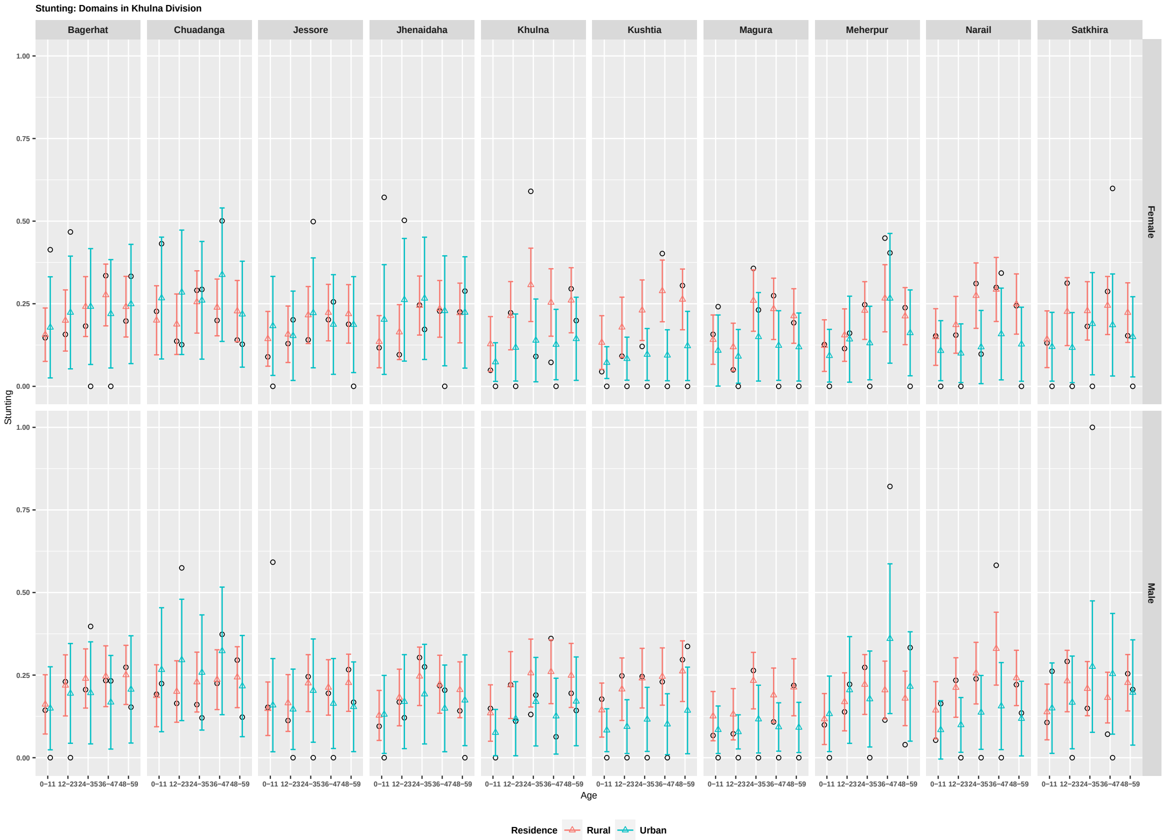

Fig. S.29 Prevalence of stunting for the cross-classified domains of district, residence, children age-group and sex belong to Khulna division estimated by design-based (circle dot) and model-based (triangle dot) estimators (with 95% CI) .

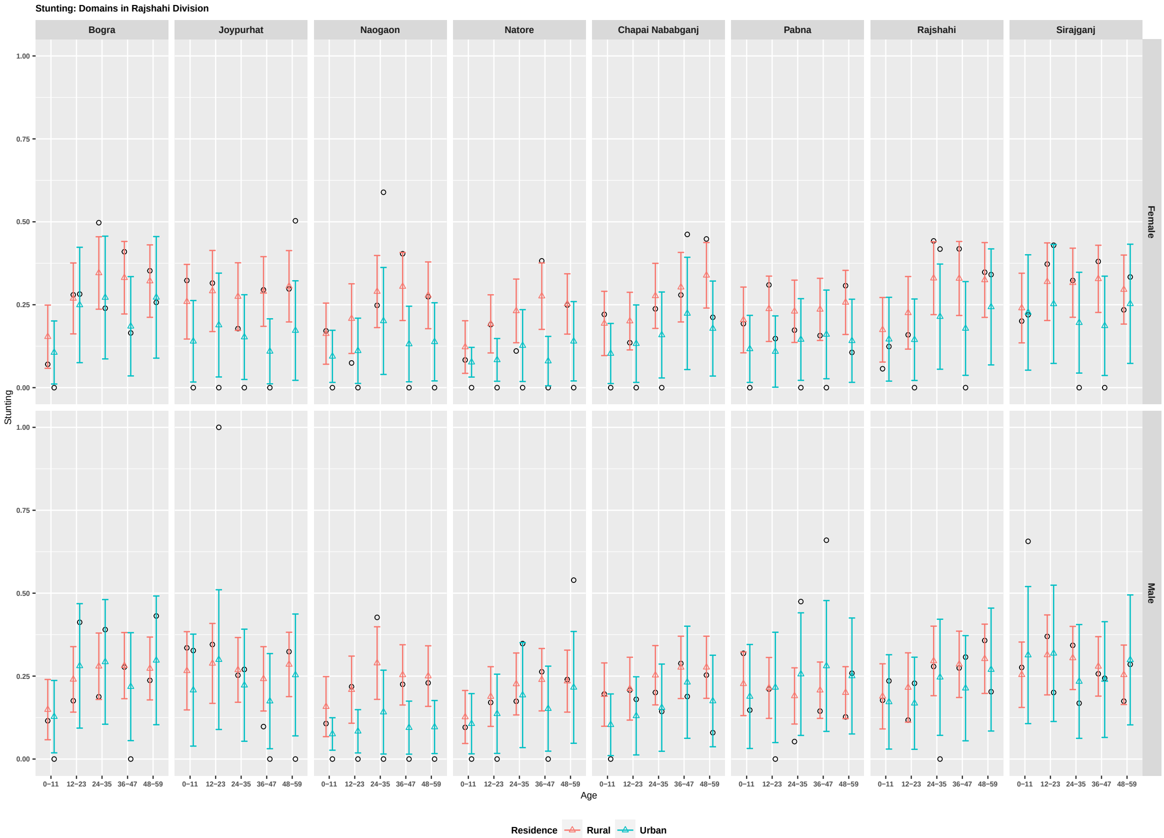

Fig. S.30 Prevalence of stunting for the cross-classified domains of district, residence, children age-group and sex belong to Rajshahi division estimated by design-based (circle dot) and model-based (triangle dot) estimators (with 95% CI) .

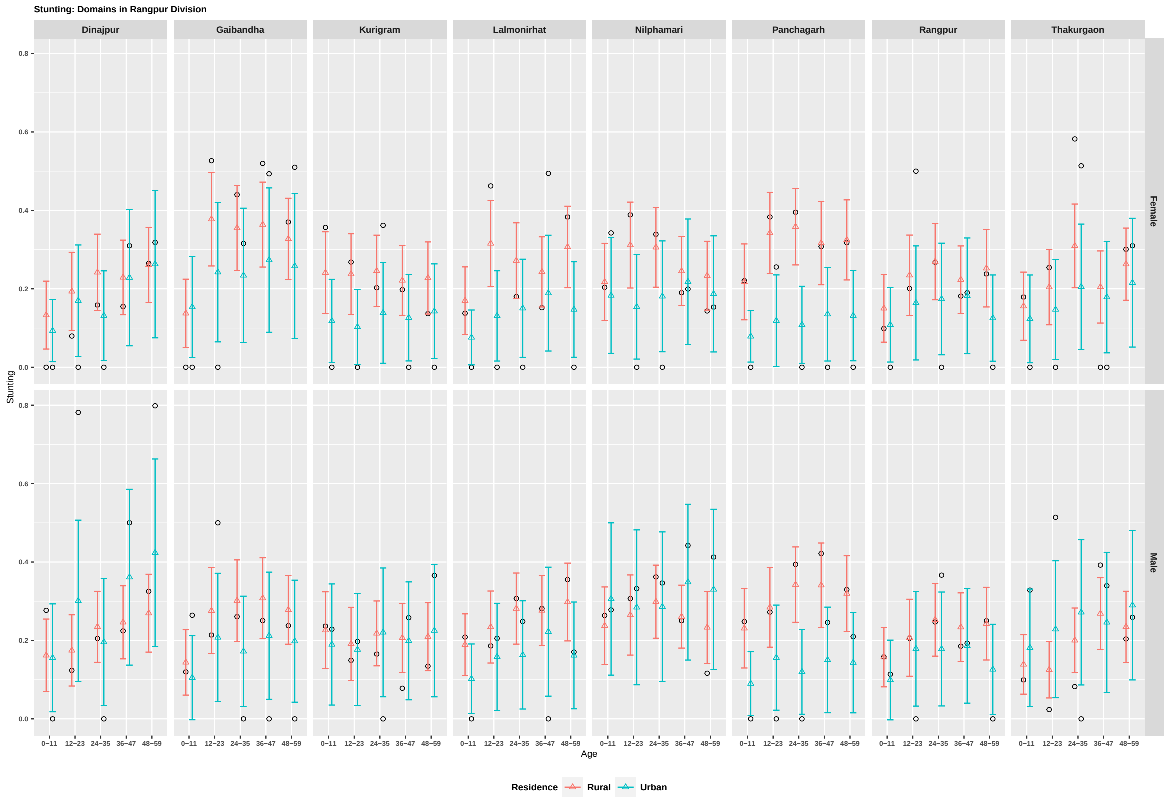

Fig. S.31 Prevalence of stunting for the cross-classified domains of district, residence, children age-group and sex belong to Rangpur division estimated by design-based (circle dot) and model-based (triangle dot) estimators (with 95% CI) .

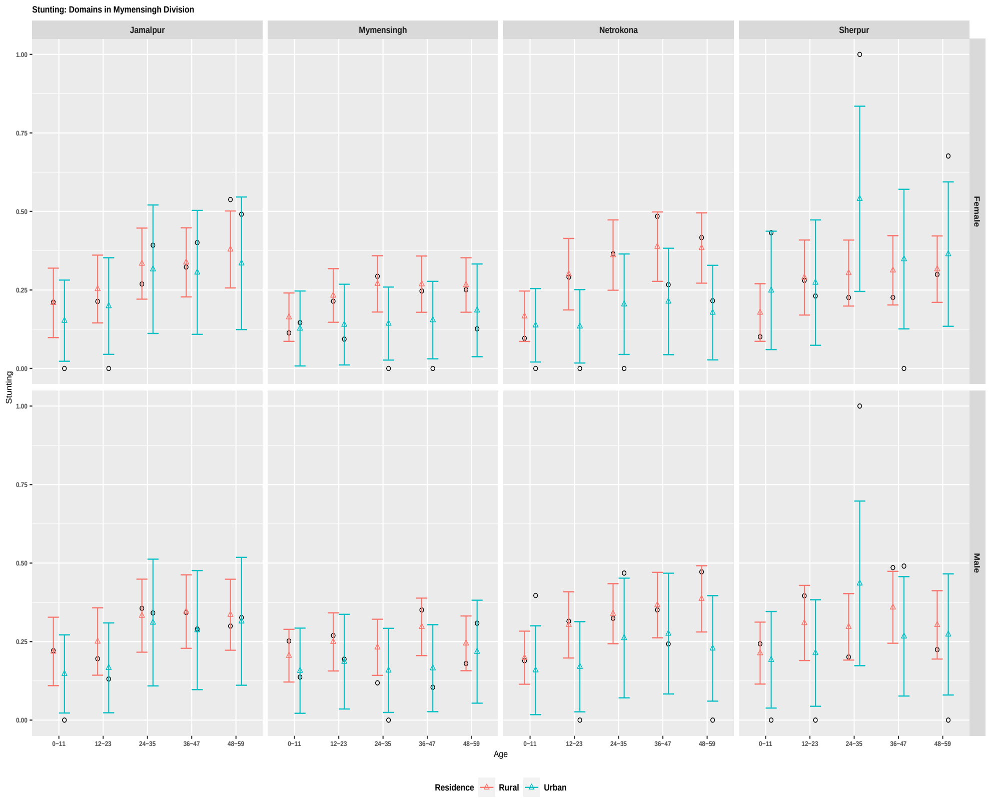

Fig. S.32 Prevalence of stunting for the cross-classified domains of district, residence, children age-group and sex belong to Mymensingh division estimated by design-based (circle dot) and model-based (triangle dot) estimators (with 95% CI) .

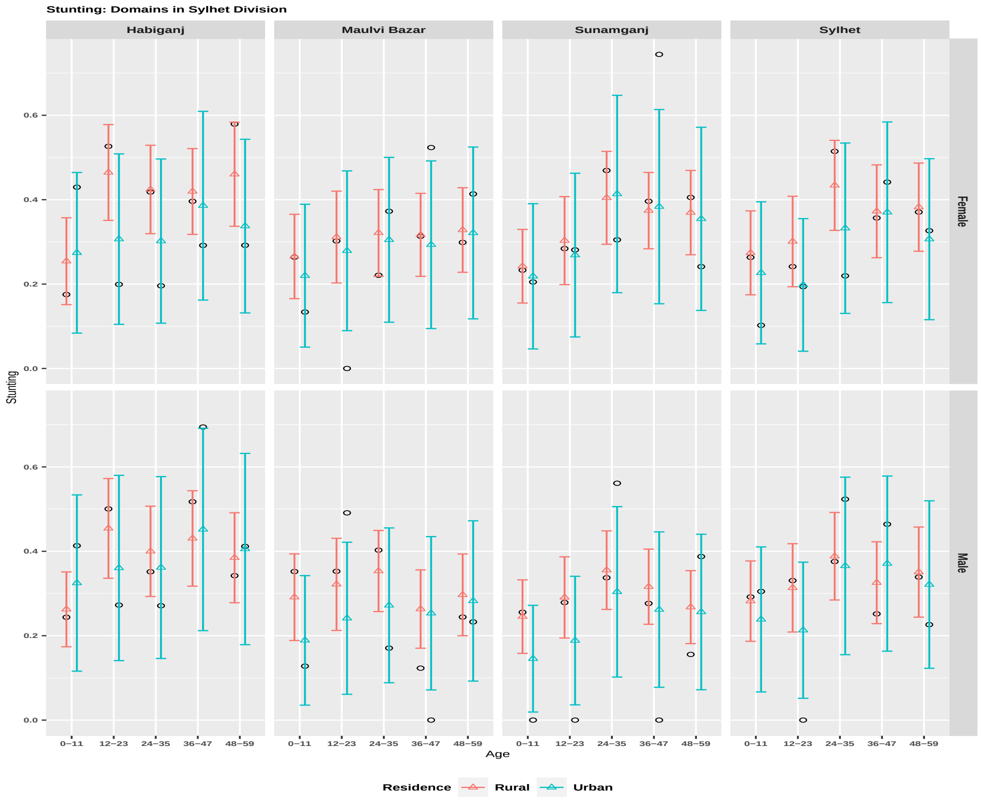

Fig. S.33 Prevalence of stunting for the cross-classified domains of district, residence, children age-group and sex belong to Sylhet division estimated by design-based (circle dot) and model-based (triangle dot) estimators (with 95% CI) .

S.10 Detailed level prevalence of Wasting

Figure S.34 shows comparison of model-based and design-based estimates of wasting at the detailed level for Barguna, Bandarban, Dhaka, Sherpur, Khulna, Sirajgonj, Dinajpur and Sunamganj districts as representatives of 8 divisions respectively. In addition, the detailed district-age-sex-residence level wasting estimated by model-based and design-based estimators are plotted in Figures S.35 to S.42 by their respective divisions.

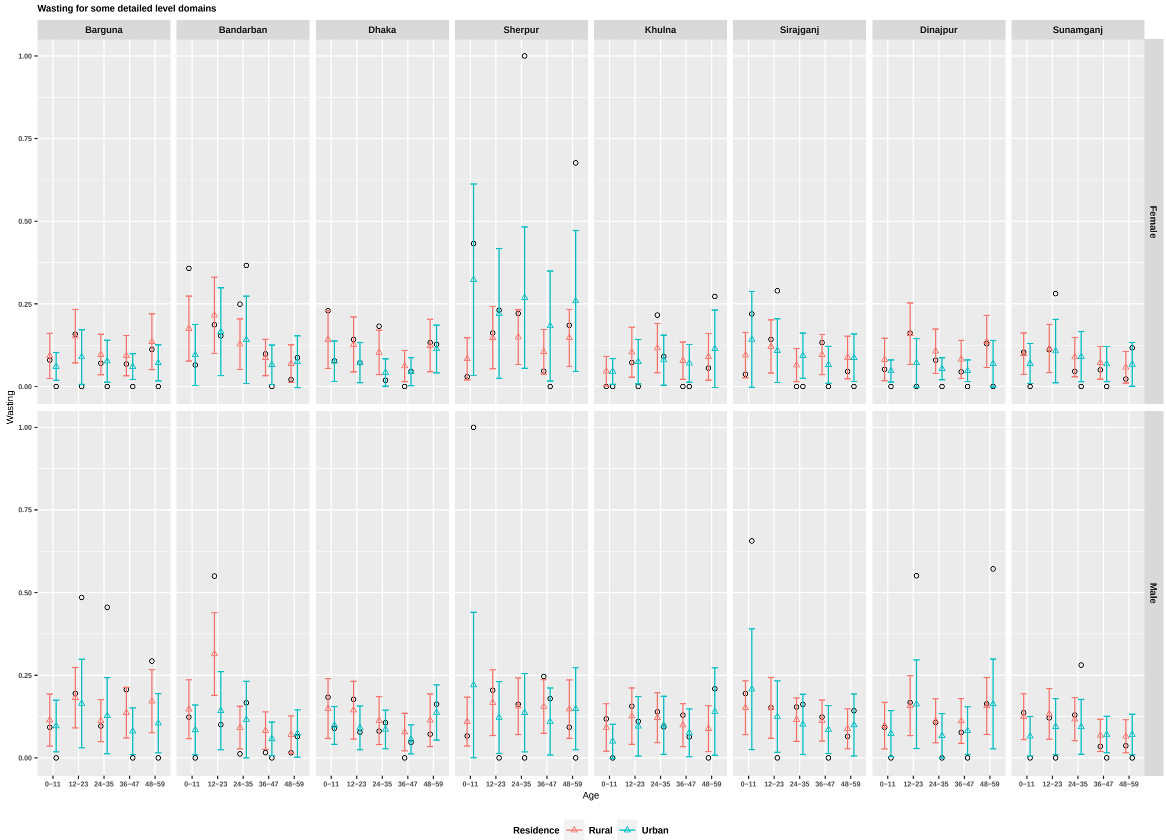

Fig. S.34 Prevalence of wasting for the detailed level domains of 8 districts as representative of 8 divisions estimated by design-based (black circle dot) and model-based (triangle coloured dots) estimators (with 95% CI) .

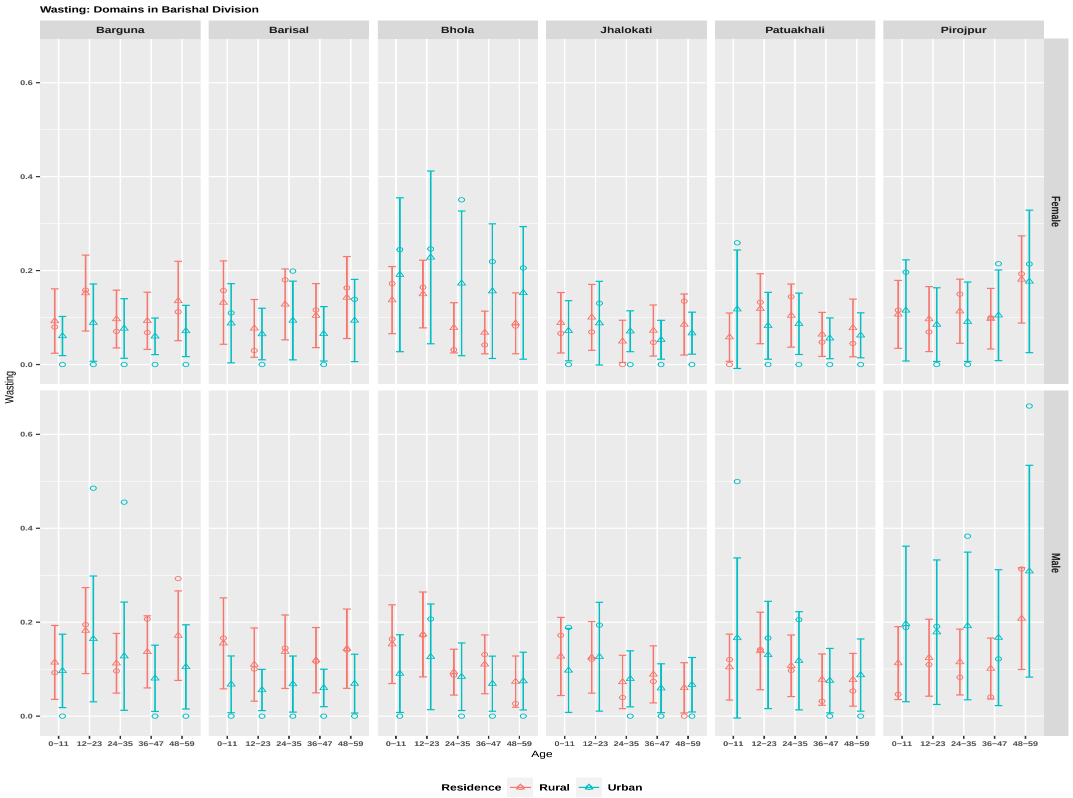

Fig. S.35 Prevalence of wasting for the cross-classified domains of district, residence, children age-group and sex belong to Barishal division estimated by design-based (circle dot) and model-based (triangle dot) estimators (with 95% CI) .

Springer Nature 2021 LaTeX template

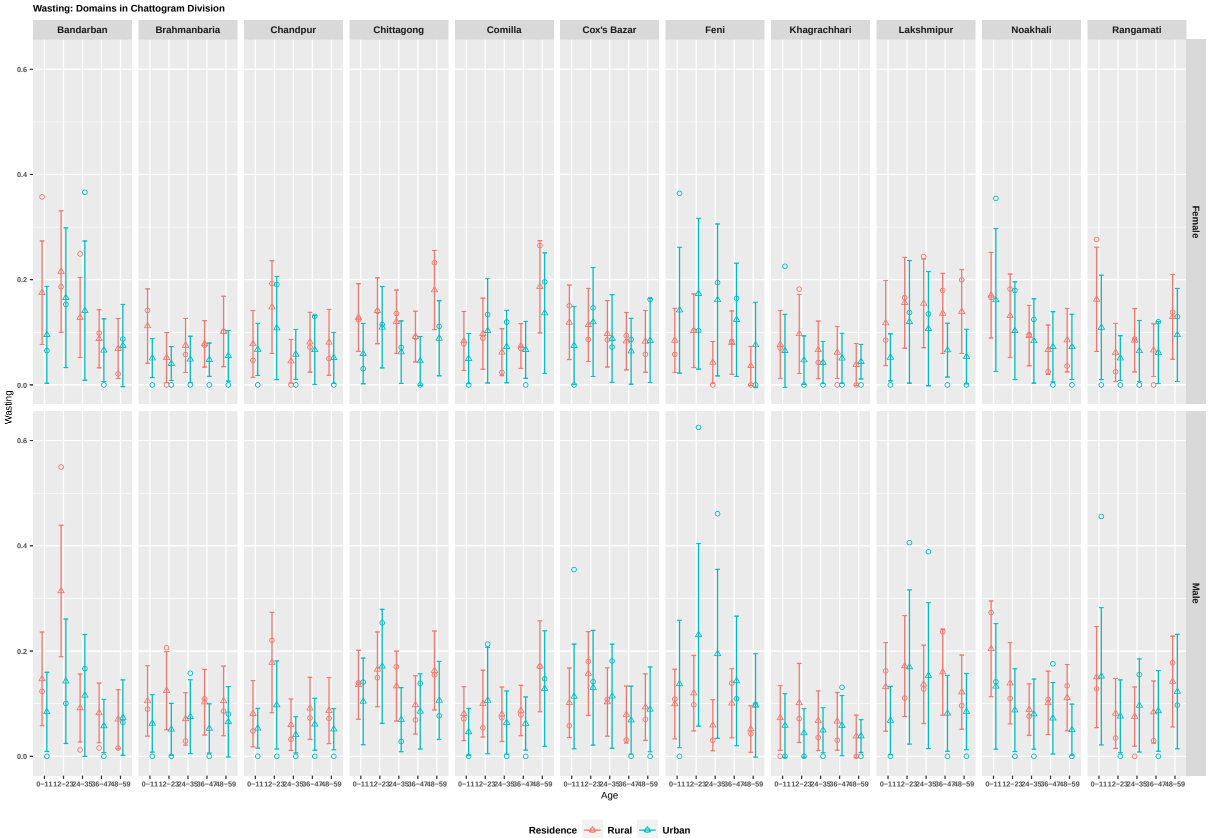

Fig. S.36 Prevalence of wasting for the cross-classified domains of district, residence, children age-group and sex belong to Chattogram division estimated by design-based (circle dot) and model-based (triangle dot) estimators (with 95% CI) .

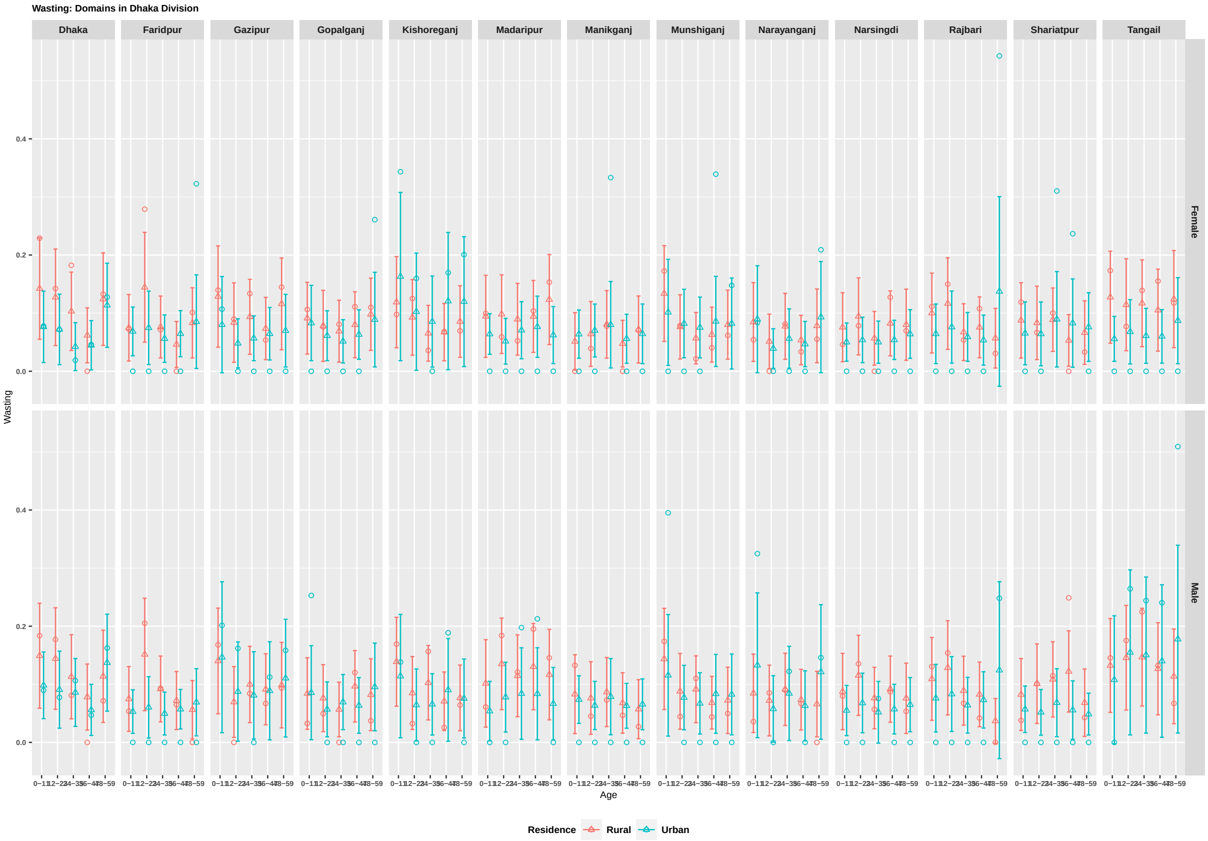

Fig. S.37 Prevalence of wasting for the cross-classified domains of district, residence, children age-group and sex belong to Dhaka division estimated by design-based (circle dot) and model-based (triangle dot) estimators (with 95% CI) .

Springer Nature 2021 LaTeX template

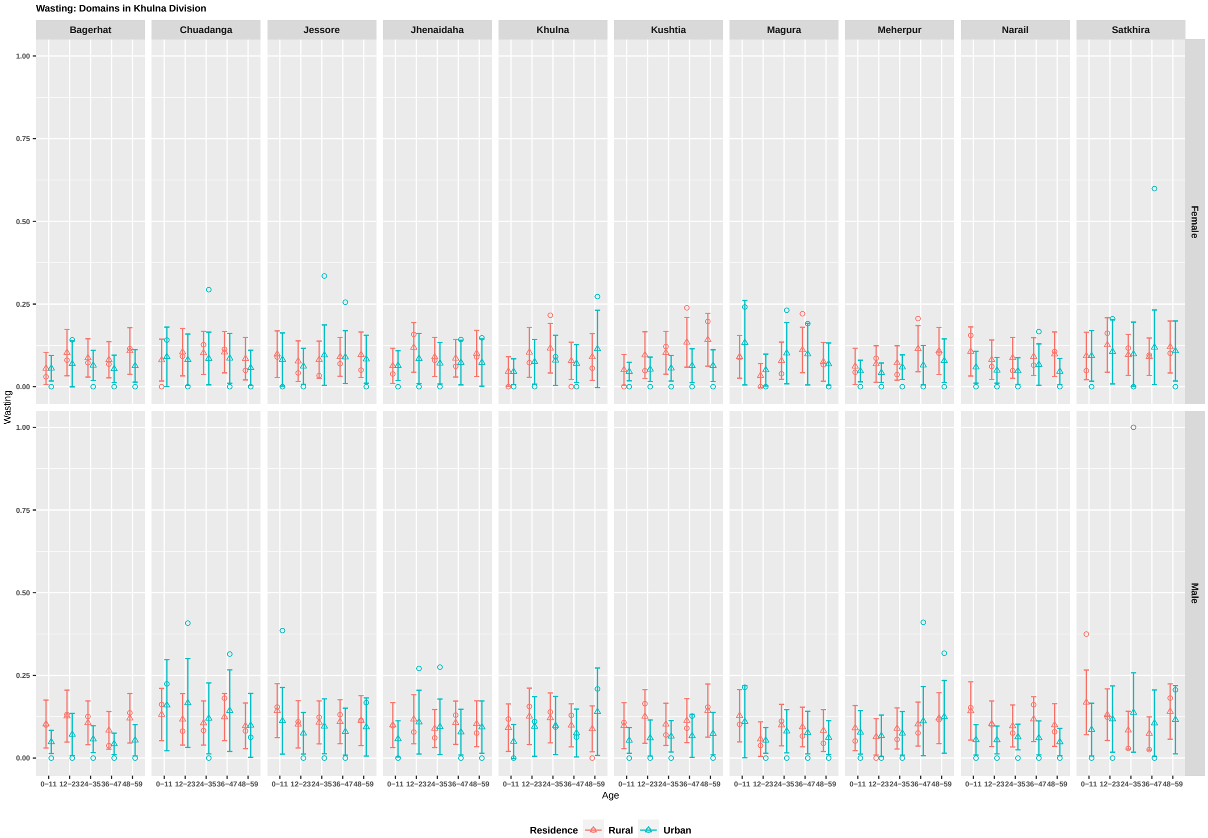

Fig. S.38 Prevalence of wasting for the cross-classified domains of district, residence, children age-group and sex belong to Khulna division estimated by design-based (circle dot) and model-based (triangle dot) estimators (with 95% CI) .

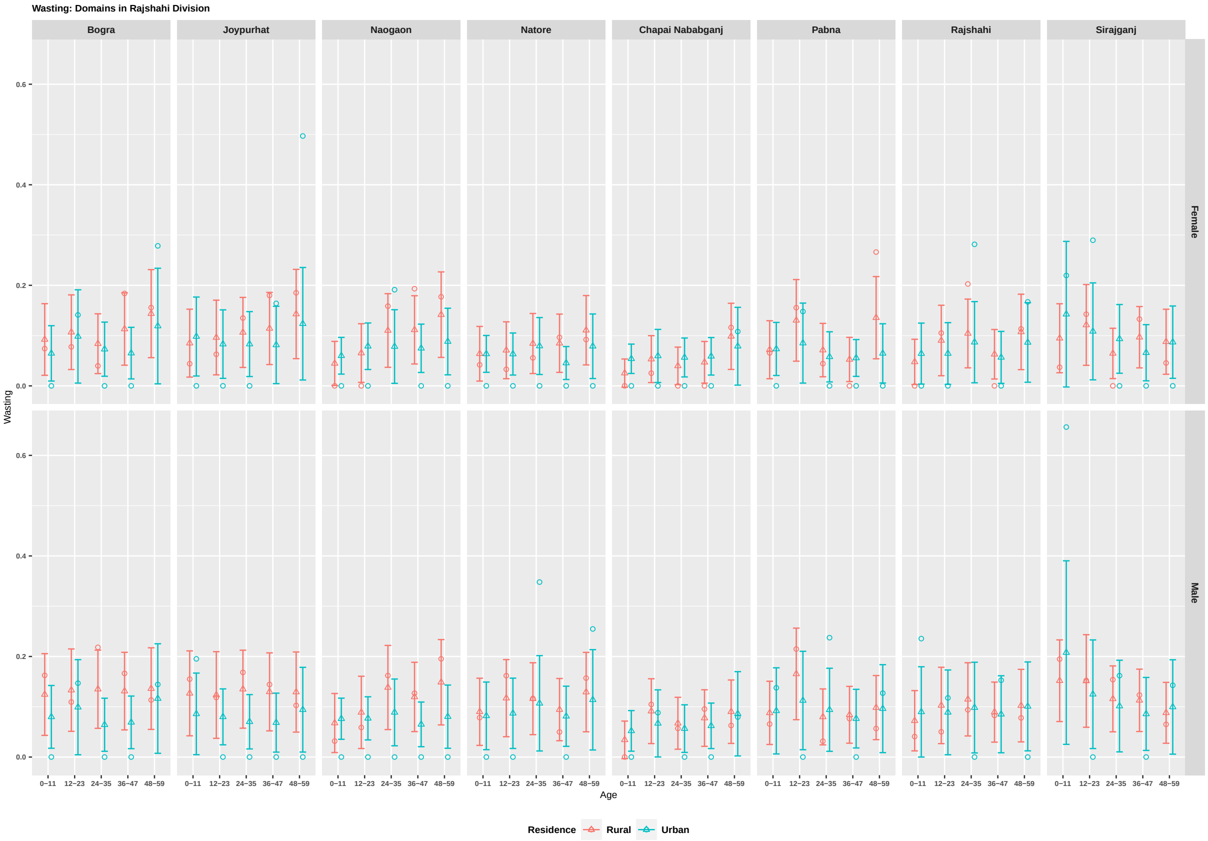

Fig. S.39 Prevalence of wasting for the cross-classified domains of district, residence, children age-group and sex belong to Rajshahi division estimated by design-based (circle dot) and model-based (triangle dot) estimators (with 95% CI) .

Springer Nature 2021 LaTeX template

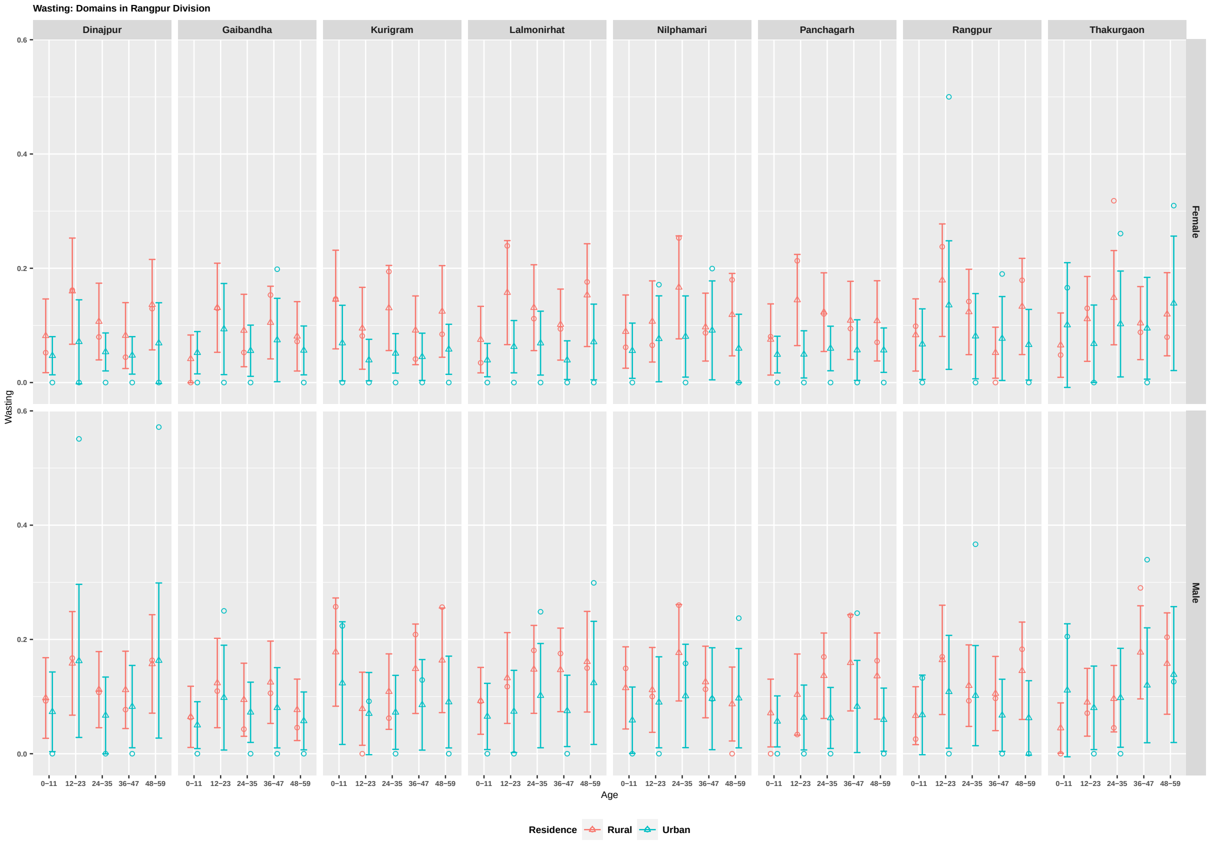

Fig. S.40 Prevalence of wasting for the cross-classified domains of district, residence, children age-group and sex belong to Rangpur division estimated by design-based (circle dot) and model-based (triangle dot) estimators (with 95% CI) .

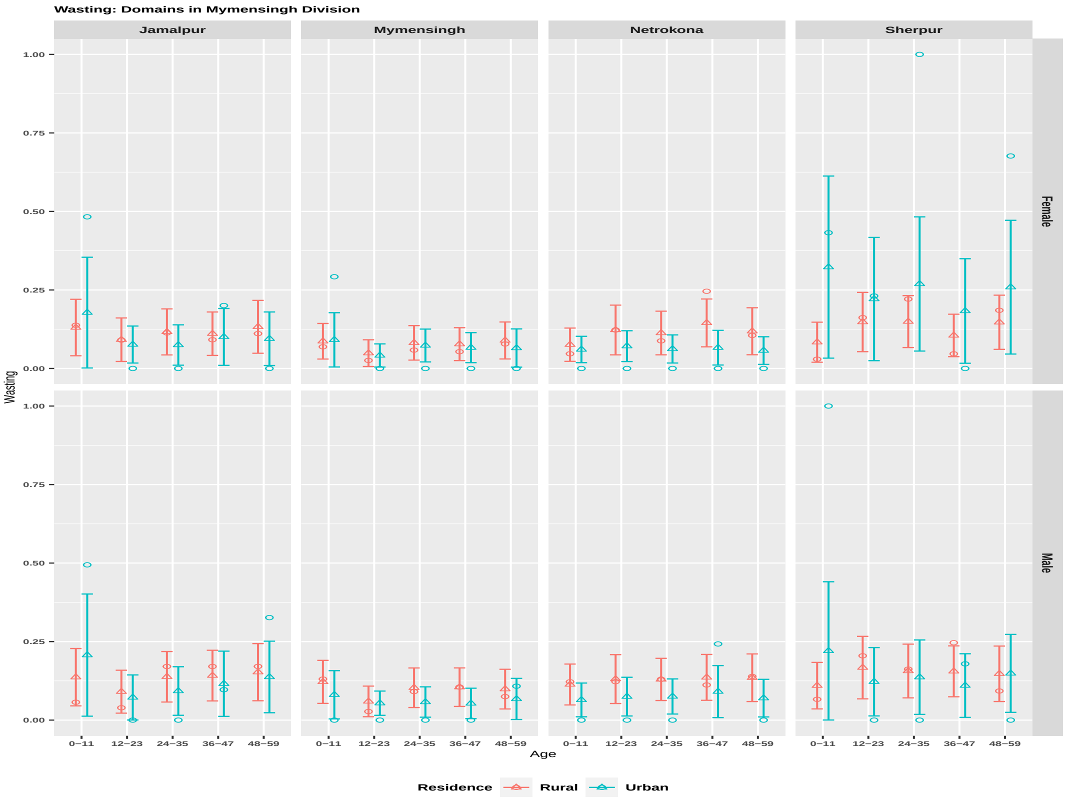

Fig. S.41 Prevalence of wasting for the cross-classified domains of district, residence, children age-group and sex belong to Mymensingh division estimated by design-based (circle dot) and model-based (triangle dot) estimators (with 95% CI) .

Springer Nature 2021 LaTeX template

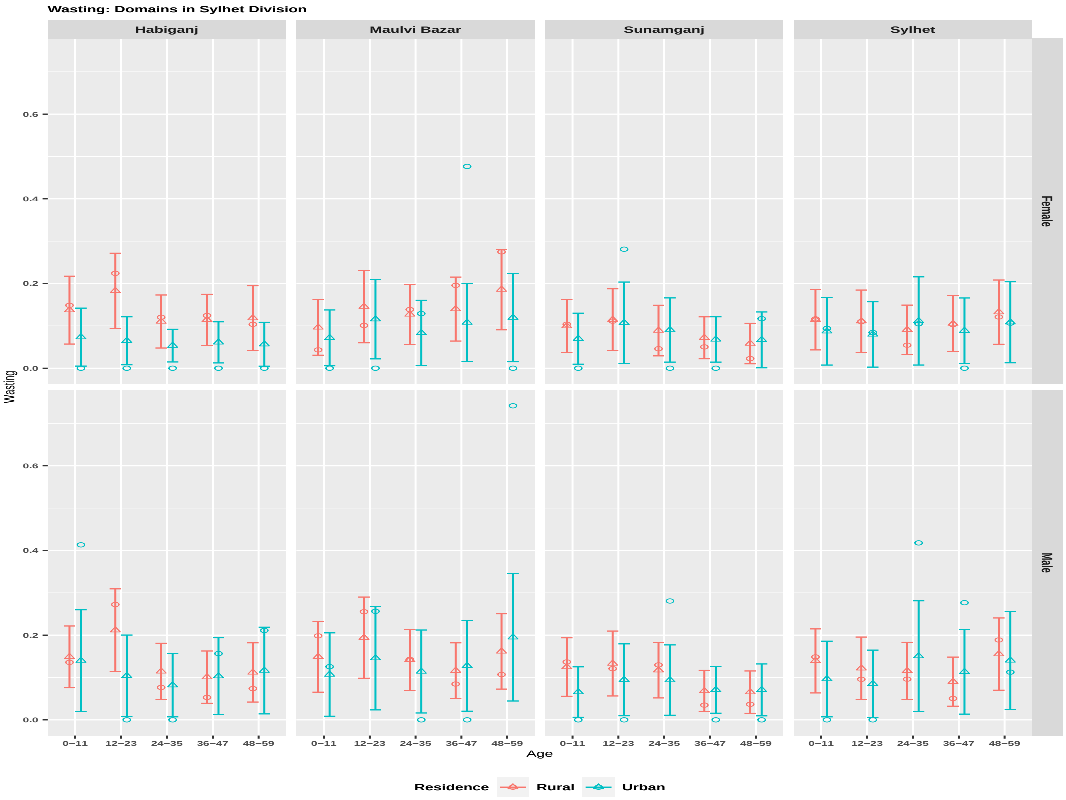

Fig. S.42 Prevalence of wasting for the cross-classified domains of district, residence, children age-group and sex belong to Sylhet division estimated by design-based (circle dot) and model-based (triangle dot) estimators (with 95% CI) .

S.11 Detailed level prevalence of Underweight

Figure S.43 shows comparison of model-based and design-based estimates of underweight at the detailed level for Barguna, Bandarban, Dhaka, Sherpur, Khulna, Sirajgonj, Dinajpur and Sunamganj districts as representatives of 8 divisions respectively. In addition, the detailed district-age-sex-residence level underweight estimated by model-based and design-based estimators are plotted in Figures S.44 to S.51 by their respective divisions.

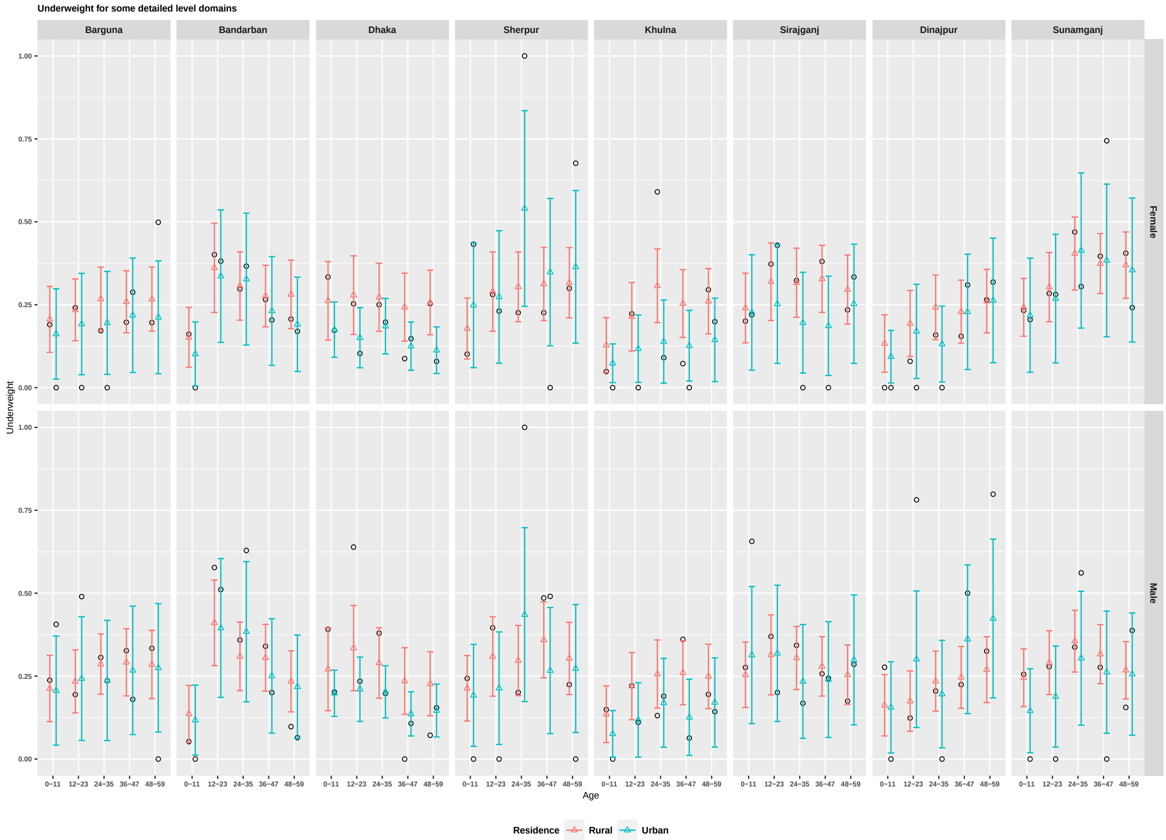

Fig. S.43 Prevalence of underweight for the detailed level domains of 8 districts as representative of 8 divisions estimated by design-based (black circle dot) and model-based (triangle coloured dots) estimators (with 95% CI) .

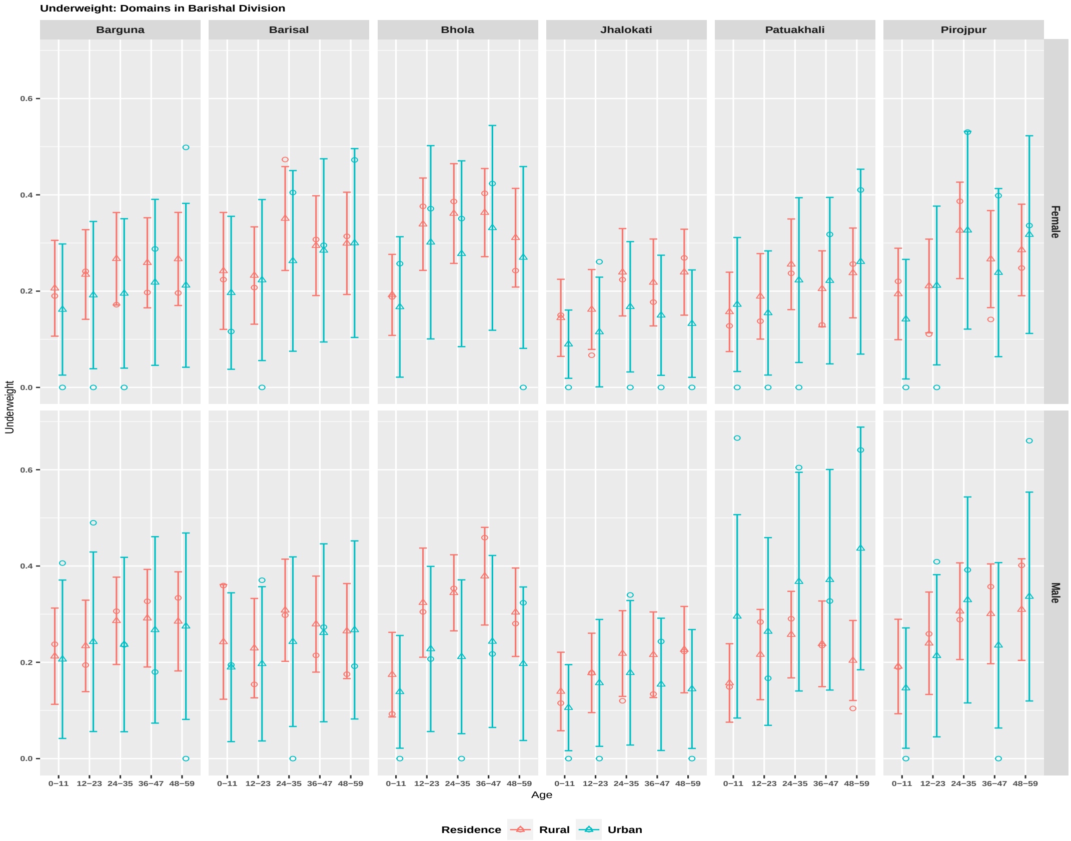

Fig. S.44 Prevalence of underweight for the cross-classified domains of district, residence, children age-group and sex belong to Barishal division estimated by design-based (circle dot) and model-based (triangle dot) estimators (with 95% CI) .

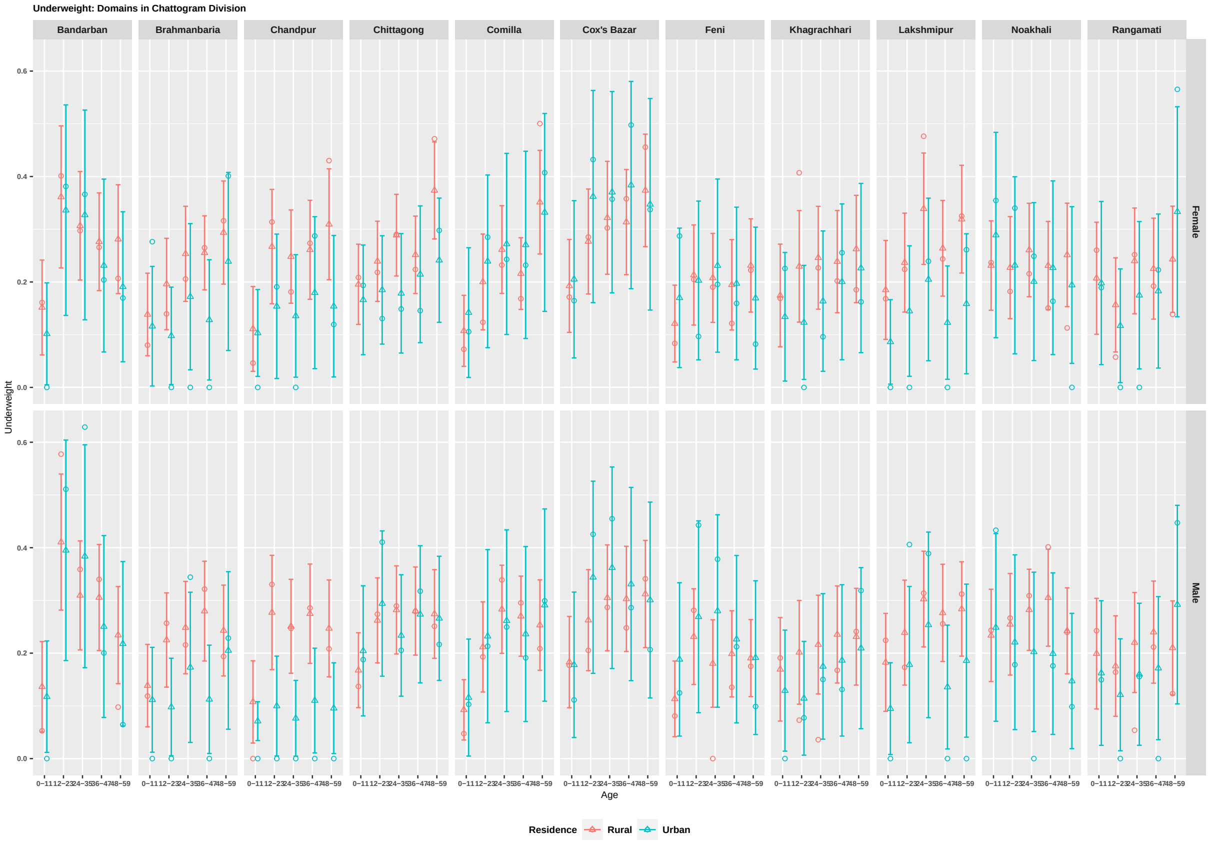

Fig. S.45 Prevalence of underweight for the cross-classified domains of district, residence, children age-group and sex belong to Chattogram division estimated by design-based (circle dot) and model-based (triangle dot) estimators (with 95% CI) .

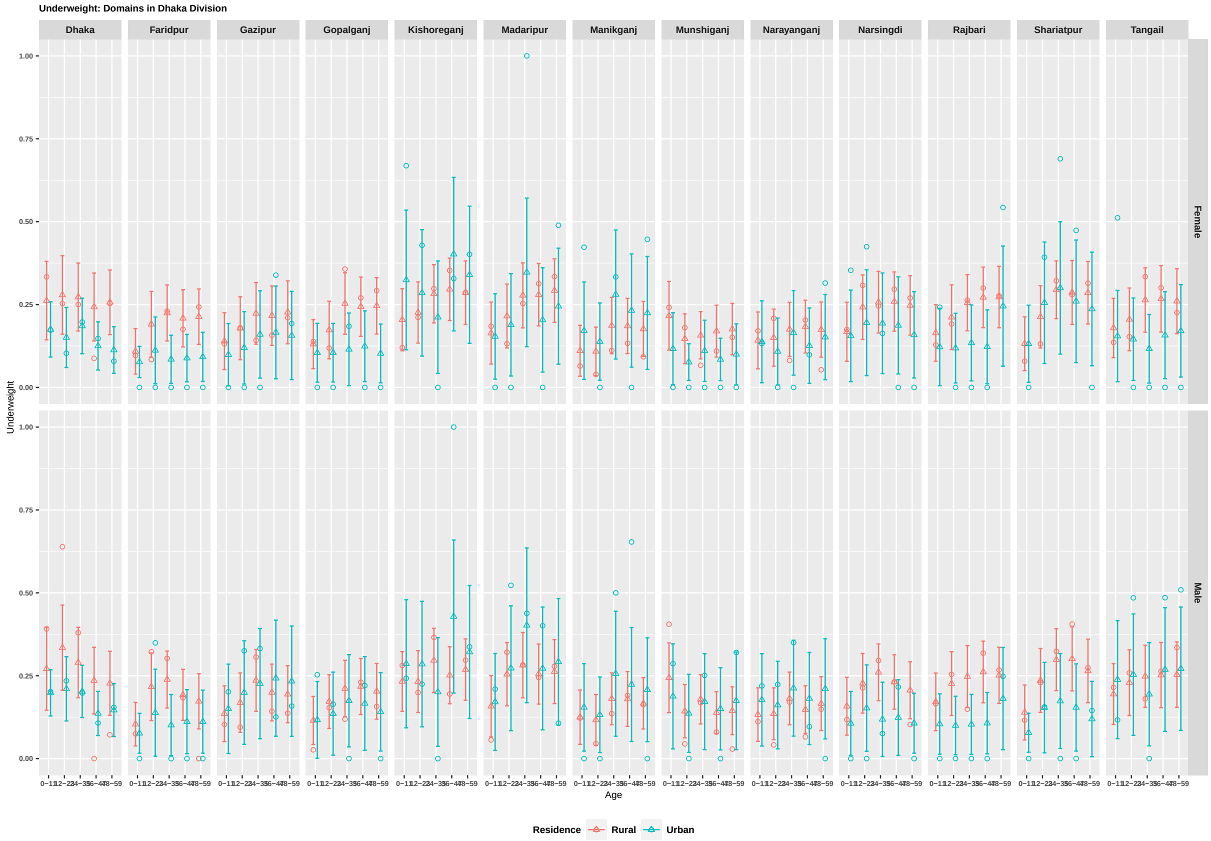

Fig. S.46 Prevalence of underweight for the cross-classified domains of district, residence, children age-group and sex belong to Dhaka division estimated by design-based (circle dot) and model-based (triangle dot) estimators (with 95% CI) .

Springer Nature 2021 LaTeX template

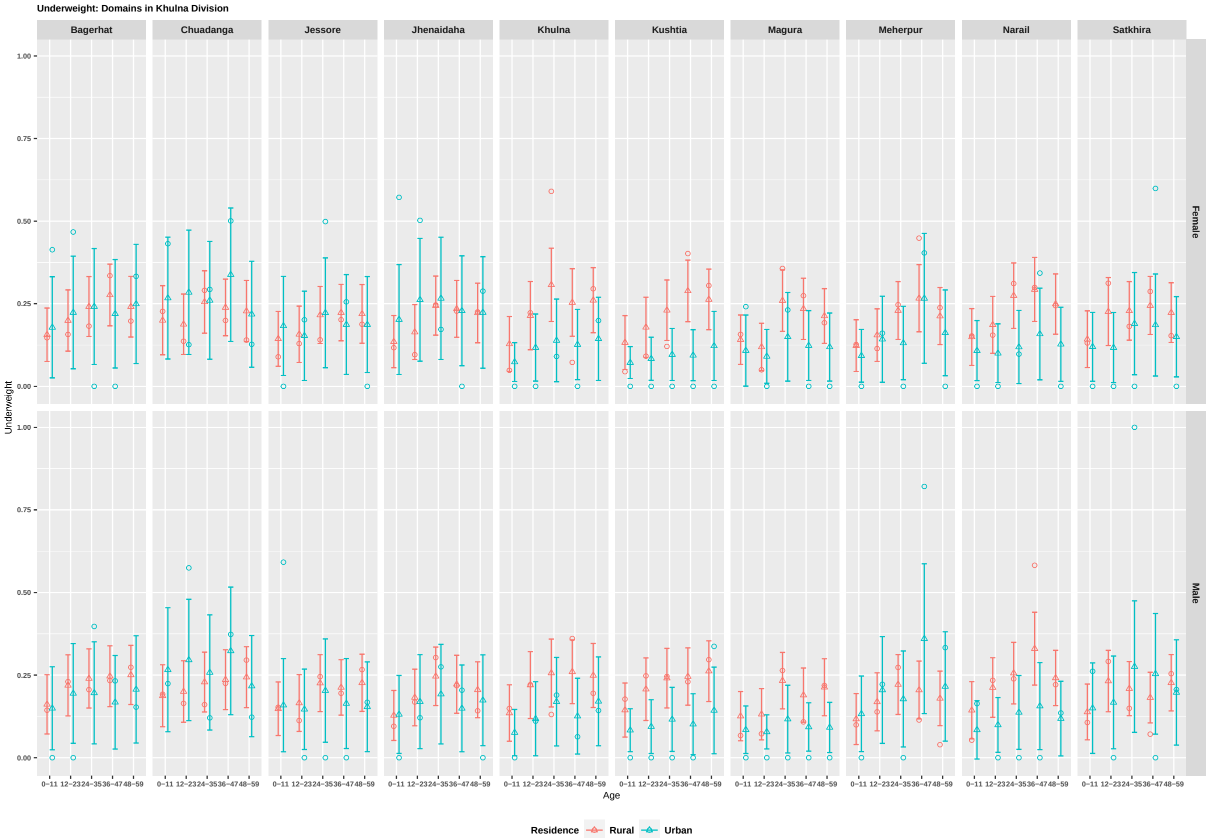

Fig. S.47 Prevalence of underweight for the cross-classified domains of district, residence, children age-group and sex belong to Khulna division estimated by design-based (circle dot) and model-based (triangle dot) estimators (with 95% CI) .

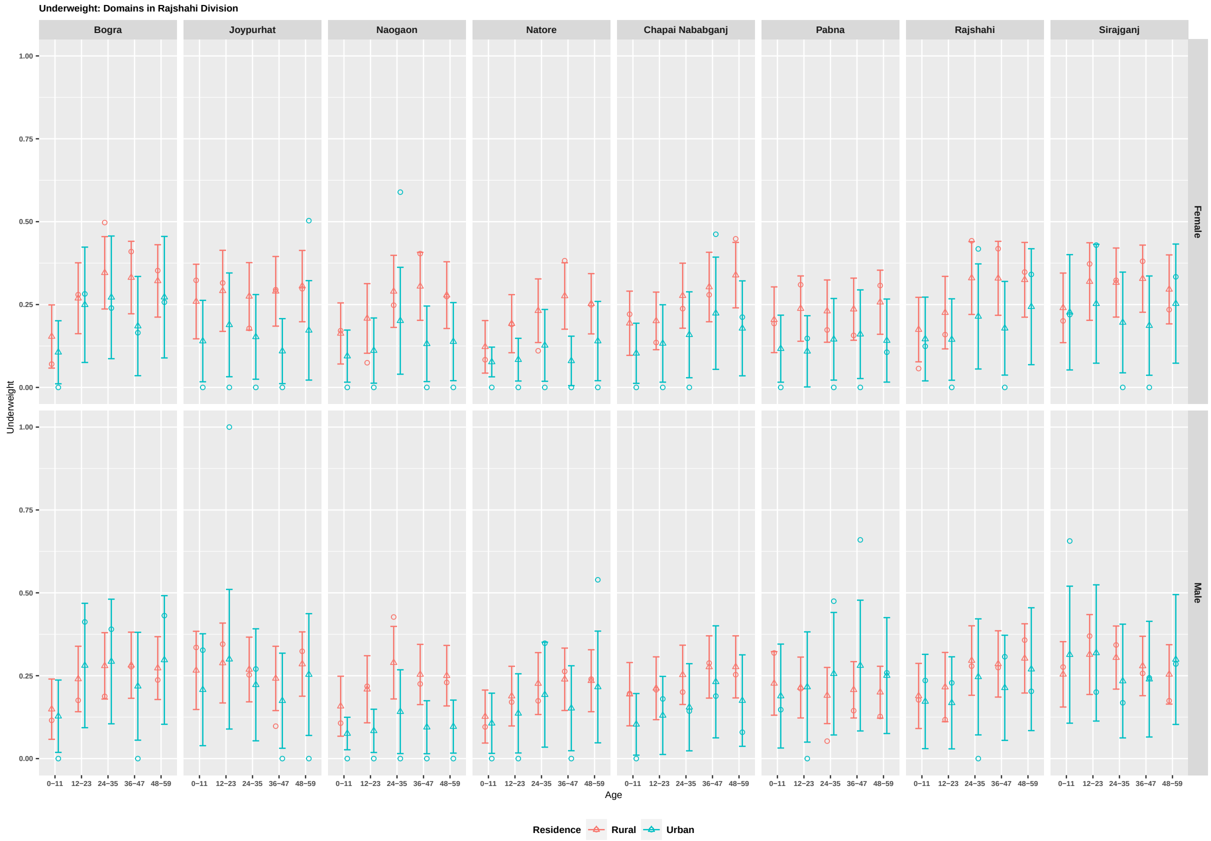

Fig. S.48 Prevalence of underweight for the cross-classified domains of district, residence, children age-group and sex belong to Rajshahi division estimated by design-based (circle dot) and model-based (triangle dot) estimators (with 95% CI) .

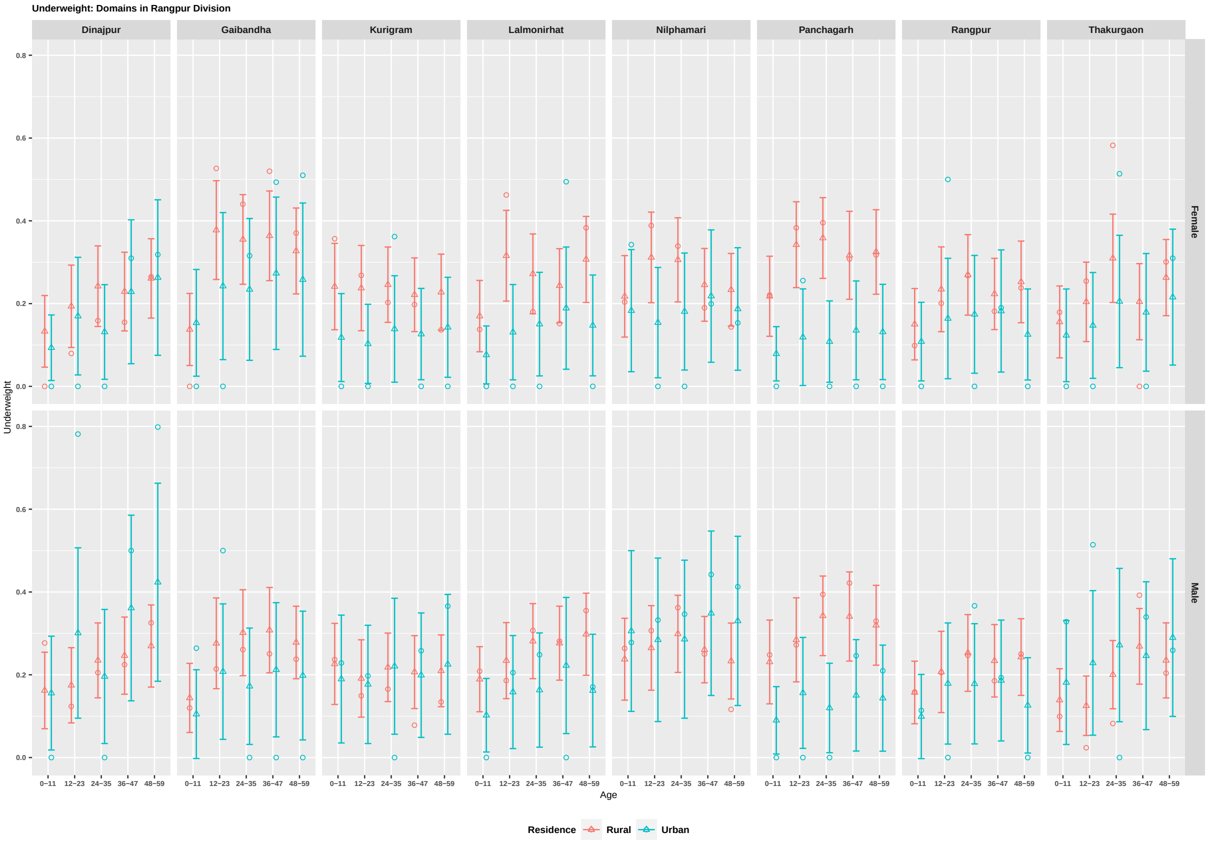

Fig. S.49 Prevalence of underweight for the cross-classified domains of district, residence, children age-group and sex belong to Rangpur division estimated by design-based (circle dot) and model-based (triangle dot) estimators (with 95% CI) .

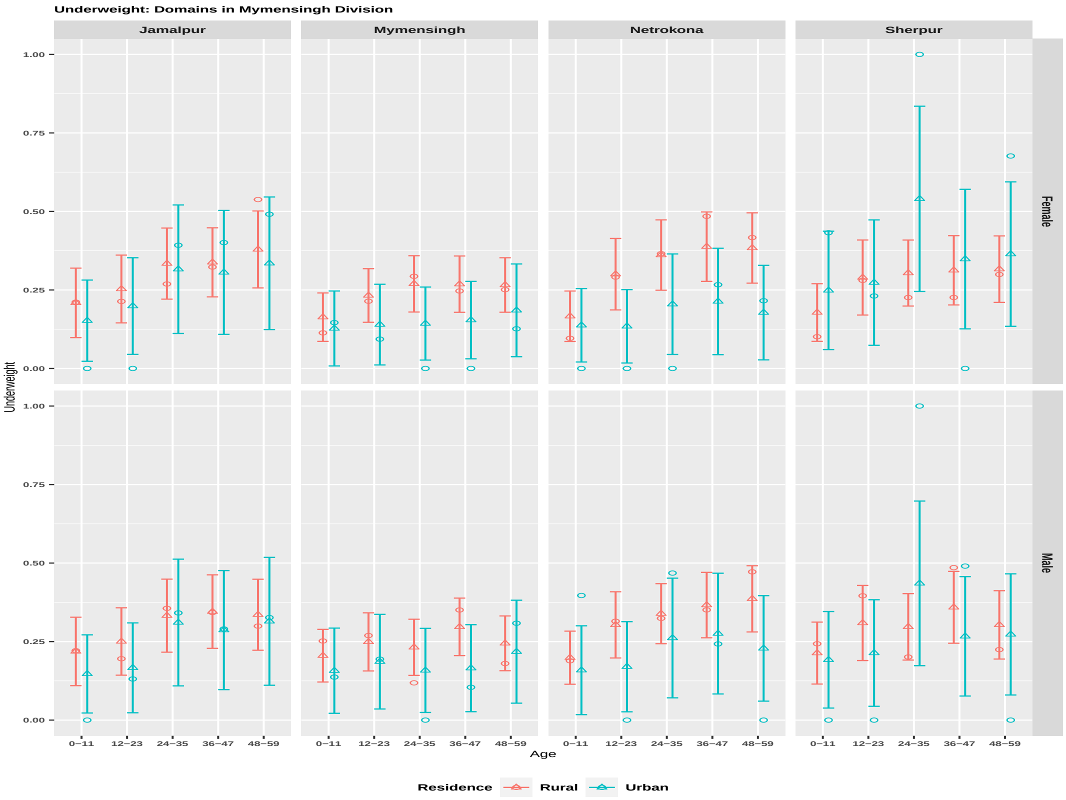

Fig. S.50 Prevalence of underweight for the cross-classified domains of district, residence, children age-group and sex belong to Mymensingh division estimated by design-based (circle dot) and model-based (triangle dot) estimators (with 95% CI) .

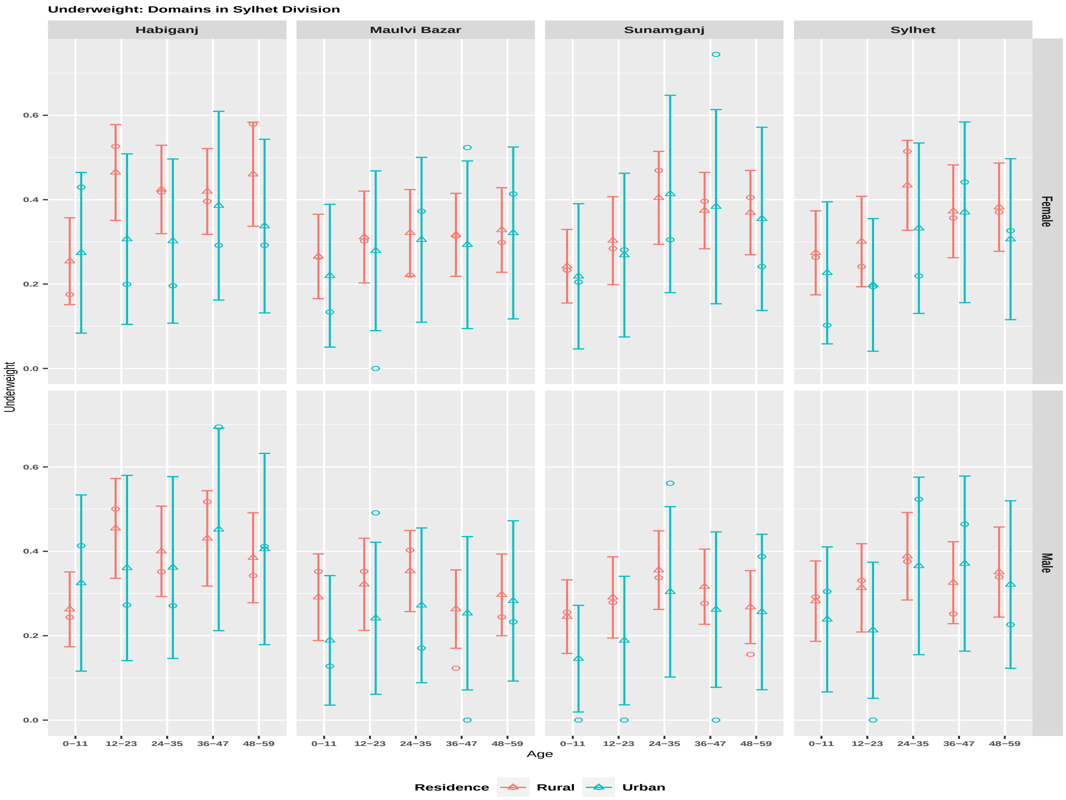

Fig. S.51 Prevalence of underweight for the cross-classified domains of district, residence, children age-group and sex belong to Sylhet division estimated by design-based (circle dot) and model-based (triangle dot) estimators (with 95% CI) .

References

[1] BBS, UNICEF. Progotir Pathey Bangladesh: Bangladesh Multiple Indicator Cluster Survey 2019. Bangladesh Bereau of Statistics (BBS); 2019.

[2] Gelman A, Hill J. Data analysis using regression and multilevel/hierarchical models. Cambridge University Press; 2007.

[3] Gelman A. Prior distributions for variance parameters in hierarchical models. Bayesian Analysis. 2006;1(3):515–533.

[4] R Core Team.: R: A Language and Environment for Statistical Computing. Vienna, Austria. Available from: http://www.R-project.org.

[5] Boonstra HJ.: mcmcsae: MCMC Small Area Estimation. R package version 0.7.0. Available from: https://CRAN.R-project.org/package=mcmcsae.

[6] Watanabe S. Asymptotic equivalence of Bayes cross validation and widely applicable information criterion in singular learning theory. Journal of Machine Learning Research. 2010;11:3571–3594.

[7] Watanabe S. A widely applicable Bayesian information criterion. Journal of Machine Learning Research. 2013;14:867–897.

[8] Spiegelhalter DJ, Best NG, Carlin BP, van der Linde A. Bayesian Measures of Model Complexity and Fit. Journal of the Royal Statistical Society B. 2002;64(4):583–639.

[9] Gelman A, Rubin DB. Inference from iterative simulation using multiple sequences. Statistical Science. 1992;7(4):457–472.
